# Multiscale physiologically-based model of age-dependent CD4+ T-lymphocyte homeostasis

**DOI:** 10.3389/fimmu.2026.1742817

**Published:** 2026-02-04

**Authors:** Victoria Kulesh, Kirill Peskov, Gabriel Helmlinger, Gennady Bocharov

**Affiliations:** 1 Research Center of Model-Informed Drug Development, Sechenov First Moscow State Medical University, Moscow, Russia; 2 Marchuk Institute of Numerical Mathematics of the Russian Academy of Sciences (INM RAS), Moscow, Russia; 3 Lomonosov Moscow State University, Moscow, Russia; 4 Modeling & Simulation Decisions FZ-LLC, Dubai, United Arab Emirates; 5 Quantitative Medicines Consulting, Boston, MA, United States; 6 Institute for Computer Science and Mathematical Modelling, Sechenov First Moscow State Medical University, Moscow, Russia; 7 Moscow Center of Fundamental and Applied Mathematics at INM RAS, Moscow, Russia

**Keywords:** age-dependent homeostasis, CD4+ T-lymphocytes, mechanistic modeling, thymectomy, thymus involution, aging

## Abstract

**Objective:**

To develop a mechanistic physiologically-based model describing CD4^+^ T-lymphocyte homeostasis across the human lifespan, incorporating maturation, differentiation, migration aspects and the impact of age on distinct cell subpopulations.

**Methods:**

A stepwise modeling approach was implemented by integrating published quantitative data on CD4^+^ T-cell concentration in blood and various tissues for narrowly defined age ranges, together with experimental kinetic parameters. The homeostatic CD4^+^ T-lymphocyte kinetics model was represented as a system of ordinary differential equations for four thymocyte subpopulations and six CD4^+^ T-lymphocyte subpopulations, incorporating five physiological compartments: the thymus, blood, lymphoid tissue, the gastro-intestinal tract, and lung tissue. A series of empirical functions was sequentially tested to describe age-related changes in homeostasis. Reciprocal cellular feedback functions were assessed for incorporation in the model, as an alternative to age-dependent functions. An extensive set of model evaluations was performed, including model validation on total and memory CD4^+^ T-cell concentrations, simulations of homeostasis perturbations following thymectomy, and global sensitivity analysis, to determine the processes most influential in shaping CD4^+^ T-cell homeostasis.

**Results:**

Age-related shifts in proliferation of naïve and activated cells, differentiation of memory subsets, survival of recent thymic emigrants (RTE) and migration aspects of CD4^+^ T-cells – together with reduced thymic output – were identified as key determinants of immune homeostasis. Sensitivity analyses showed that thymocyte and naïve cell homeostasis drives early differentiation stages, whereas clonal expansion dominates memory and effector cell maintenance, with the influence of all processes declining with age. Although increased naïve T-cell proliferation and reduced RTE death may partially compensate for thymic loss, these mechanisms are insufficient to restore long-term CD4^+^ T-cell counts after thymectomy.

**Conclusion:**

By unifying diverse clinical and experimental observations within a multiscale mechanistic quantitative framework, the proposed model offers a robust tool for predicting CD4^+^ T-cell dynamics and assessing the impact of physiological changes or interventions on immune homeostasis.

## 1 Introduction

CD4^+^ T-lymphocytes represent a key component of adaptive immunity, responsible for orchestrating and regulating human immune reactions. The mission of these cells extends beyond the assisting of cytotoxic CD8^+^ T-lymphocytes and B-lymphocytes in their functions, activation of antigen-presenting cells, induction of early innate inflammatory responses, immune memory maintenance and other protecting functions (1). T-lymphocytes are widely targeted by newly developed immunotherapy medications, especially in Oncology applications. Genetically modified T-lymphocytes by chimeric antigen receptor (CAR) are used as immunotherapeutic agents in the treatment of hematological oncological diseases and solid tumors (2). Other types of immunotherapies, such as immune checkpoint inhibitors and the emerging T-cell engagers also interact with CD4^+^ T-lymphocytes, by virtue of their targeting of malignant cells (3). Dysfunctions of these immune cells contribute to a potentially severe deterioration of human immunity. CD4^+^ T-cells are the main targets of the human immunodeficiency virus (HIV); their depletion and exhaustion lead to acquired immunodeficiency syndrome (AIDS). Moreover, these cells are highly involved in autoimmune reactions (4). Homeostatic properties of CD4^+^ T-lymphocytes, together with their regulation and quantification are of great interest to researchers in fundamental and clinical immunology, in their journey to both unravel unknown immune mechanisms and develop novel targeted pharmacological treatments and vaccines (5).

T-cell development begins in the thymus gland and continues through peripheral maturation with sequential differentiation from recent thymic emigrants (RTE) to naïve T-cells (6). The first encounter with a foreign antigen leads to the activation of specific naïve cells coupled with clonal expansion and formation of both effector and memory phenotypes (7). Memory CD4^+^ T-cells comprise a heterogeneous subpopulation of cells, including stem cell-like-, central-, effector-, and resident-memory subsets, and provide rapid and robust responses upon re-exposure to antigens (8). Cell migration relies on a differentiation stage of cell surface and expression of homing markers, i.e., CCR7^+^ and CD62L^+^ (9).

Next to differentiation and transition aspects, another homeostatic property of CD4^+^ T-lymphocytes and the immune system in general pertains to age-dependent alterations. Aging of the immune system is a complex and multifactorial physiological process that unfolds across various biological scales, from molecular signaling pathways to cellular populations and entire tissues. It encompasses a wide range of changes, including a gradual thymus involution reducing mature T-lymphocytes output, systemic inflammation and immunosenescence, revealing functional impairment of immune cells (10). These changes contribute to a progressive decline in immune system function, affecting its ability to eliminate infectious agents or malignant cells.

The investigation of immune T-cells homeostasis, their kinetics and distribution aspects as well as age-related changes continues to receive high attention from experimental and theoretical immunologists. A large variety of experimental *in vitro* and *in vivo* data, obtained by flow cytometry, BrdU of deuterium labeling methods, signal joint TCR excision circles (TRECs) content measurement and others, have been used to explore and quantify T-cell homeostasis (5). Computational modeling is one quantitative tool to describe kinetics of immune cells; among other features, it has provided well-established estimates of cellular turnover rates (11, 12). However, in order to further sort through intricate mutual influences of immune cells in the context of homeostasis, it is necessary to consider aspects of cell differentiation and migration throughout the organism.

In the study of age-dependent homeostasis of T-lymphocytes, several questions related to the nature of compensatory mechanisms on the age-related decrease in thymic output, as well as mechanisms of T-cell memory longevity remain unresolved (13, 14). Most of the experimental research in the field is based on “young” vs. “old” group comparisons, which hampers the extrapolation of results across multiple age groups spanning the human lifespan (15). To derive further detailed insights on the mechanisms of age-related changes and the most critical determinants of T-cell homeostasis, a thorough curation of the available quantitative information on T-lymphocyte homeostasis, with respect to the characteristics of the performed experiments (particularly in terms of the age of the studied subjects) is required. There have been several attempts to evaluate the age dynamics of immune cell counts, based on large amounts of quantitative data (16–18). In the recent work of Schröter et al., age-related changes of lymphocyte concentrations were mathematically described, using a modified exponential decay function with a delay, based on an extensive dataset of specific data on various cell subpopulations (17). Schröter and colleagues were among the first to quantitatively identify the age dynamics of cells, in the first years of human life. We recently performed a systematic review and meta-analysis, gathering all available quantitative information from multiple sources, on concentrations of multiple lymphocyte subpopulations in blood and peripheral organs (18). As a result, we obtained generalized estimates of cell concentrations across narrow age groups. While a further standalone description of distinct immune cell subpopulations may provide an accurate quantitative capture of cell homeostasis, it may not allow to decipher specific mechanisms of age-related changes and their regulation.

The primary objective of the present study was 1) to quantitatively characterize age-related homeostasis of CD4^+^ T-lymphocyte subpopulations, by curating information and data on cellular kinetics and age-dependent dynamics and 2) to integrate these into a quantitative, physiologically-based mathematical model. The homeostatic model was developed with respect to the biology of differentiation, proliferation and migration of multiple subpopulations of CD4^+^ T-lymphocytes. Combining multi-level data into a mechanistic model enabled the investigation of changes in cellular homeostasis in response to perturbations, to test the hypothesis on the mechanisms of age-related alterations in homeostasis and to determine which homeostatic processes are most influenced by age-related changes.

## 2 Materials and methods

### 2.1 Data

#### 2.1.1 Clinical data on T-lymphocytes homeostasis

Clinical data on T-lymphocyte counts for several cell subpopulations spanning diverse age groups were required for homeostatic model development. Results from a previously performed systematic review and meta-analysis on age-dependent human T-lymphocyte homeostasis were used as a main source of quantitative data (18). The weighted averages as generalized estimates calculated within prespecified age intervals for each CD4^+^ T-lymphocyte subpopulation in each organ were used for parameter estimation and model validation. Quantitative data on specific subpopulations of CD4^+^ T-lymphocytes, such as RTE, naïve, activated, central-memory, effector-memory and effector cells in blood, lymphoid tissue, the gastro-intestinal tract and lungs were used for calibration purposes, while the total subpopulations were used for model validation. Subsequent gating strategies and phenotypes of these cell subpopulations were fully described in the previous work (18). To evaluate the ability of the model to predict the overall immune status with age, model validation was performed against concentrations of total CD4^+^ T-lymphocytes and of total memory (CD45RO^+^) CD4^+^ T-lymphocytes in blood and lymphoid tissue.

In addition to cell concentration data, quantitative estimates on the total blood volume for different age groups and thymus wet weight were collected (19–26). Total blood volume data were used for parameterization of its dependency with age, to obtain the model-predicted values on cell concentrations in blood (19–24). An additional thymus wet weight data source (26), with data for the first years of life was exploited to inform the previously developed model of thymocytes homeostasis in healthy subjects (27). Since this sub-model was used as one of the mechanistic model building blocks, the data on thymus wet weight were used to parameterize the age dependency of wet weight (25, 26). Moreover, to evaluate the model’s ability to capture cell dynamics under cellular homeostasis perturbations, individual data from patients who underwent complete thymectomy in their childhood, from early infancy to 6 years of age, were additionally collected (28, 29).

Detailed information on the clinical data used in the analysis, including age group ranges and evaluation procedure, is presented in the **Supplementary Table 1**.

#### 2.1.2 Experimental estimates of homeostatic processes

To obtain quantitative information on CD4^+^ T-lymphocyte subpopulation kinetics, various sources of experimental *in vitro* and *in vivo* mouse data, as well as clinical data from deuterium labeled T-cell kinetic studies and modeling results were examined (15, 30–49). Death rates for CD4^+^ T-lymphocyte subpopulations were calculated according to the half-life estimates obtained mostly from deuterium labeling kinetic data modeling (30, 32, 35–37, 46). Allometric scaling was used for the calculation of kinetic rates, from mouse data (50, 51). Transition rates of distinct T-lymphocytes across tissues were assessed following the Ganusov and Tomura analysis (48). Moreover, point estimates of mechanistic quantitative systems pharmacology (QSP) models were also used to obtain physiologically meaningful intervals for model parameters (38, 42). The proposed ranges were used to inform initial parameter values for calibration. The values of experimental ranges, as well as a detailed description of the proposed assumptions are summarized in **Supplementary Tables 2**, **3**, respectively.

### 2.2 Modeling workflow

**Figure 1:**
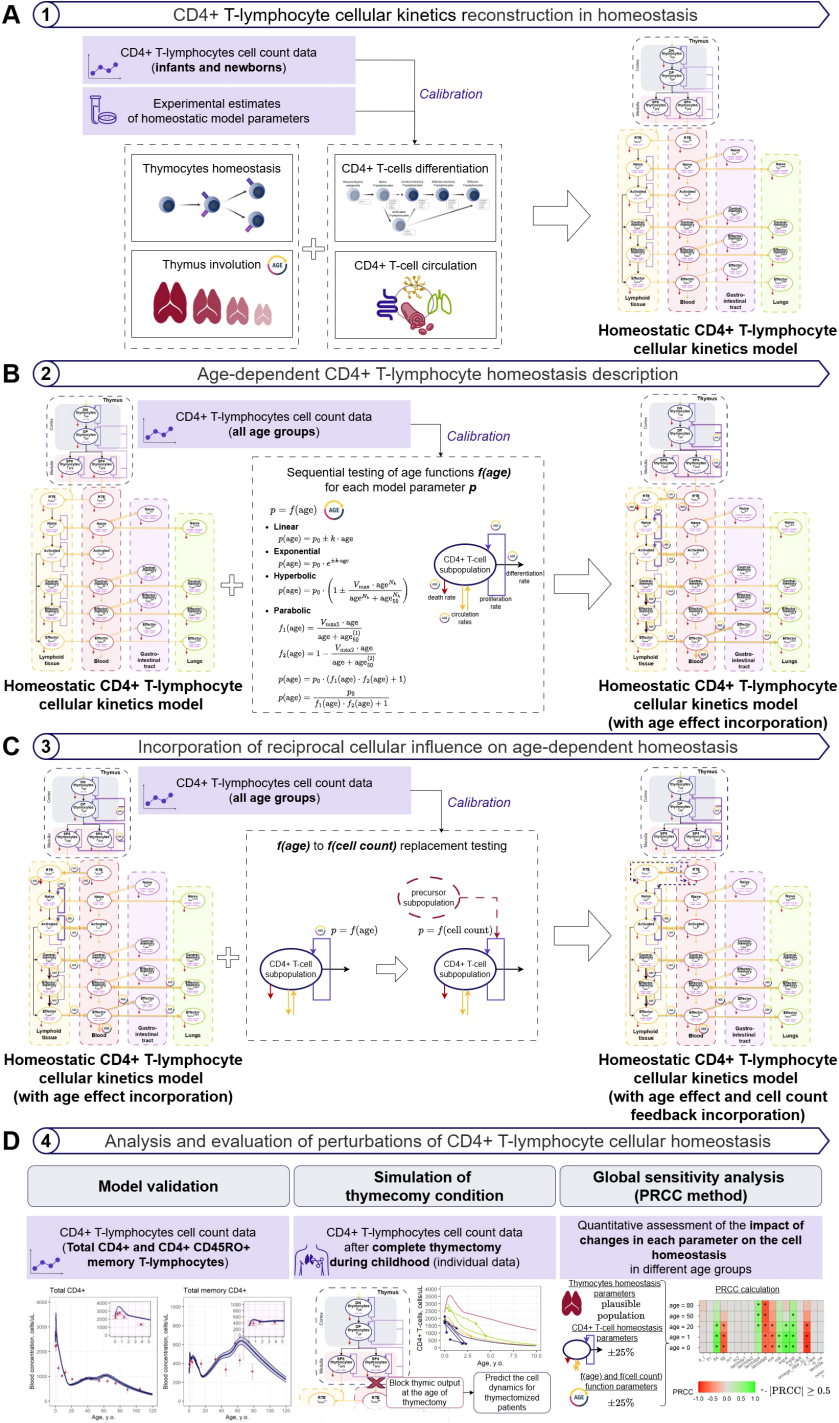
Scheme for the model-based description and analysis of age-dependent CD4^+^ T-lymphocyte homeostasis (stage 1 **(A)** – model-based description of CD4^+^ T-lymphocytes cellular kinetics in homeostasis following a multi-compartmental framework; stage 2 **(B)** – parameterization of age-dependent changes in homeostasis; stage 3 **(C)** – incorporation of intercellular regulation and its influence on age-dependent homeostasis; stage 4 **(D)** – analysis and evaluation of the effect of various perturbations on cellular age-dependent homeostasis).

A sequential modeling approach was applied to formulate, calibrate and validate the model of CD4^+^ T-lymphocytes age homeostasis. The proposed data-based workflow includes four stages (**Figure 1**):

1. Model-based description of CD4^+^ T-lymphocytes cellular kinetics in homeostasis following a multi-compartmental framework (**Figure 1A**)
2. Parameterization of age-dependent changes in homeostasis (**Figure 1B**)
3. Incorporation of intercellular regulation and its influence on age-dependent homeostasis (**Figure 1C**)
4. Analysis and evaluation of the effect of various perturbations on cellular age-dependent homeostasis (**Figure 1D**)

#### 2.2.1 Mathematical biology of CD4^+^ T-lymphocytes cellular kinetics

##### 2.2.1.1 Model structure: multiple organs and cell compartments

**Figure 2:**
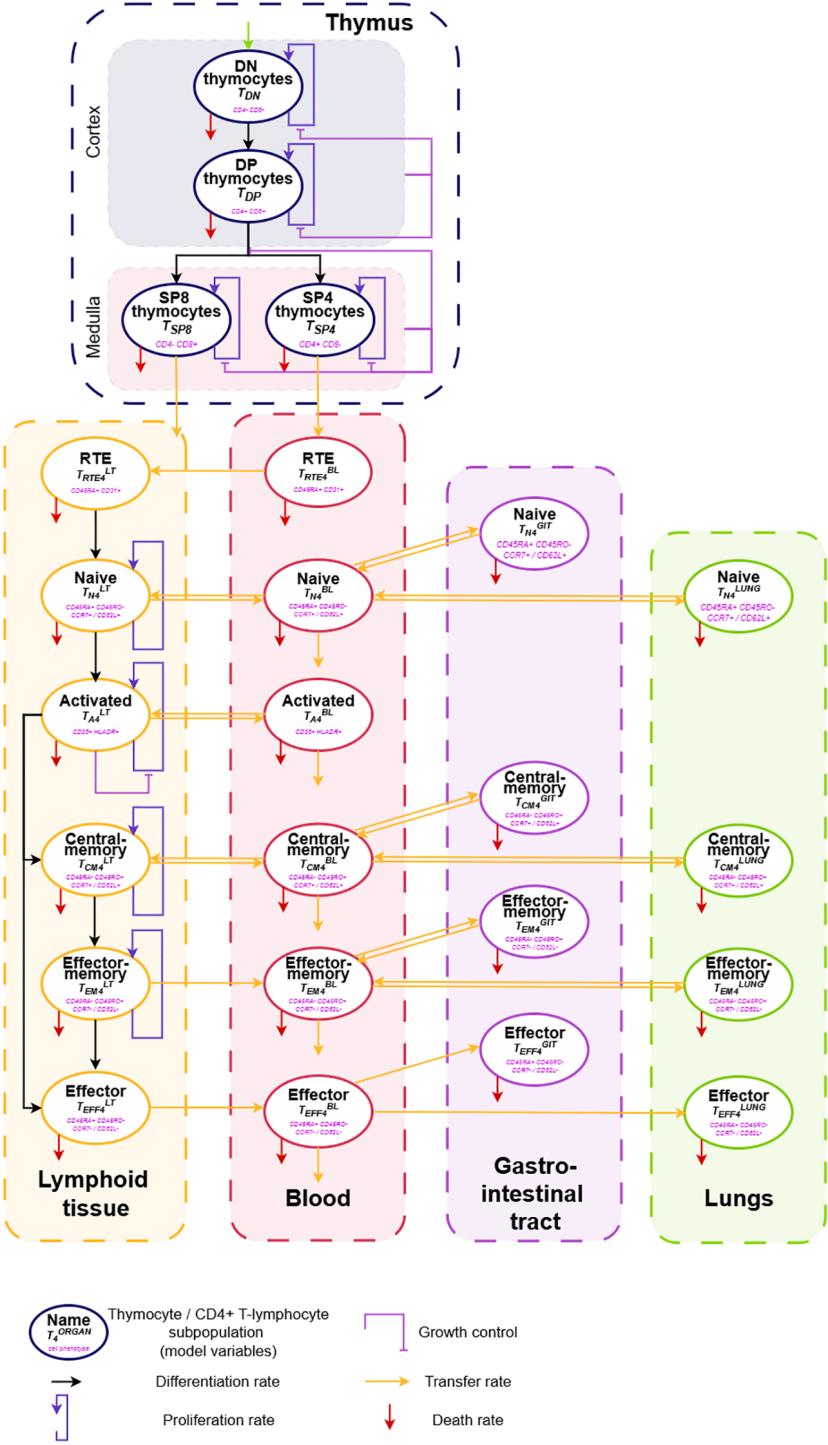
Scheme of the homeostatic CD4^+^ T-lymphocyte cellular kinetics model.

To capture T-lymphocyte homeostasis and kinetics, several key aspects needed to be accounted for, such as T-lymphocyte maturation and development, circulation capacity, distinct subpopulation differentiation and expansion properties. The complete mechanistic homeostatic CD4^+^ T-lymphocyte cellular kinetics mathematical model was formulated with a system of 24 ordinary differential equations (ODEs), with 59 parameters presented in **Supplementary Table 4**. The schematic structure of the mathematical model is shown in **Figure 2**.

To account for T-lymphocyte maturation processes, we used our previously developed mechanistic model of thymocytes homeostasis as one of the building blocks of the cellular kinetics model (27). This model described the kinetics of the main thymocyte subpopulations – double-negative (DN, CD4^–^CD8^–^), double-positive (DP, CD4^+^CD8^+^) and single-positive CD4^+^ (SP4) and CD8^+^ (SP8) cells (**Supplementary Table 4**, **Equations 6**–**9**). Moreover, the model, by virtue of incorporating thymus involution (**Supplementary Table 4**, **Equations 1**–**5**), captured age-related changes in thymocyte subpopulation counts as well as the number of egressed T-lymphocytes. As the thymus is the main source of mature T-cells, it was crucial to consider this physiological compartment within the cellular kinetics model.

Upon reaching the periphery, T-lymphocytes undergo further differentiation as a result of interactions with antigen-presentation and self-renewal mechanisms. We proposed six CD4^+^ T-lymphocytes subpopulations to be included in the cellular kinetic model: recent thymic emigrants (RTE, $T_{RTE4}$, CD31^+^ CD45RA^+^), naïve (N, $T_{N4}$, CD45RA^+^ CD45RO^–^ CCR7^+^/CD62L^+^), activated (A, $T_{A4}$, HLA-DR^+^ CD38^+^), central-memory (CM, $T_{CM4}$, CD45RA^–^ CD45RO^+^ CCR7^+^/CD62L^+^), effector-memory (EM, $T_{EM4}$, CD45RA^–^ CD45RO^+^ CCR7^–^/CD62L^–^), and effector (EFF, $T_{EFF4}$, CD45RA^+^ CD45RO^–^ CCR7^–^/CD62L^–^) cells (**Supplementary Table 4**, **Equations 16**–**35**). CD4^+^ T-lymphocytes enter the blood circulation as a distinct subpopulation of naïve T-cells – CD31^+^ RTE T-cells ( $T_{RTE4}$) which differentiate into mature naïve cells ( $T_{N4}$) in secondary lymphoid organs. Homeostatic proliferation of RTE cells ( $T_{RTE4}$) was neglected in the model, due to evidence of a lower level of proliferation, as compared to naïve cells ( $T_{N4}$) (31). Upon specific antigen encounter, naïve T-cells ( $T_{N4}$) become activated and undergo clonal expansion, which prompted us to include a distinct subpopulation of activated T-cells ( $T_{A4}$) in the model. The expansion of activated T-cells ( $T_{A4}$) was described by a logistic growth equation, to limit excessive and non-physiological cell proliferation (**Supplementary Table 4**, **Equation 22**). The subsequent differentiation of T-lymphocytes was described with respect to bifurcation theory, which favors a simultaneous formation of effector ( $T_{EFF4}$) and memory ( $T_{CM4}$ and $T_{EM4}$) T-cells (52, 53). The diversity of memory T-cell subsets was accounted for in the model, by including central-memory ( $T_{CM4}$) and effector-memory ( $T_{EM4}$) subpopulations with respect to their longevity and self-renewal mechanisms, as well as the progressive differentiation into effector cells ( $T_{EFF4}$) upon antigen re-exposure. Effector T-helper cells ( $T_{EFF4}$), as a short-lived subpopulation with immediate immune response function, were described as a terminal non-proliferating subpopulation.

Beyond the thymus, four physiological compartments – blood, lymphoid tissue, gastro-intestinal tract and lungs – were included in the model, to describe T-lymphocyte trafficking. While T-lymphocyte differentiation originates in lymphoid tissue, subsequent distribution of cells throughout the body occurs in blood. Circulatory aspects of distinct T-lymphocyte subpopulations were considered in the model structure. T-lymphocyte subsets ( $T_{N4}$ and $T_{CM4}$) that express lymph-homing markers – the adhesion molecule CD62L and the chemokine receptor CCR7 – were described as subpopulations which can migrate to other organs and recirculate to lymphatic tissue (8). In contrast, for T-lymphocyte subsets ( $T_{EM4}$ and $T_{EFF4}$) that lack expression of CCR7 and CD62L, recirculation to lymphatic tissue was not included in the model. Moreover, for effector cells ( $T_{EFF4}$), emigration to an infection site only was considered.

Several assumptions were made to reduce the dimensionality of the model parameter space. Firstly, the death rate for each of the proposed T-lymphocyte subsets was assumed to be the same for each physiological compartment, except for blood, where the first-order disappearance rate represents not only cell apoptosis but also distribution to other organs. Secondly, the migration rates from blood to/from the gastro-intestinal tract and to/from the lungs were assumed to be the same within each T-cell subpopulation. This assumption is supported by the similar cell percentage in these organs during the first year of life (18). The validity of the proposed assumptions was tested in a sensitivity analysis.

##### 2.2.1.2 Model calibration via data-driven parameterization

To calibrate CD4^+^ T-lymphocyte cellular kinetics parameters, cell concentration data from the youngest age group for each cell subpopulation within each physiological compartment were applied. The youngest age group was chosen for calibration, to initially omit any age-related processes which may influence cellular homeostasis. The proposed dataset of 20 datapoints, which included weighted averages of cell concentrations (cells/μL for blood and % for other compartments) for the specific age interval were used as steady-state values for each model variable (18). The age of the “youngest” interval for each subpopulation was set to 0 and considered as a predictor variable (regressor) in the model, to calculate maximal cortex and medulla capacity in the thymocyte homeostasis sub-model, and to subsequently account for the extent of T-cells egressed from the thymus (27).

Several structural model adaptations were made accordingly, during the model calibration phase. Firstly, as model variables represented numbers of cells in distinct compartments (**Supplementary Table 4**, **Equations 16**–**35**), a calculation of cell concentrations was performed. For example, in lymphatic tissue, the gastro-intestinal tract and the lung compartments, percentages of cell subpopulations were calculated based on the sums of the model variable values within the compartments (**Supplementary Table 4**, **Equations 50**–**63**). To convert numbers of cells in blood to measured cell concentrations (cells/μL) (**Supplementary Table 4**, **Equations 36**–**41**), an age-dependent function of blood volume was used (**Supplementary Table 4**, **Equation 15**). The parameterization of this dependency was based on a mathematical model of body weight vs age (**Supplementary Table 4**, **Equations 10**–**14**) (54). The blood volume was scaled relative to body weight using individual and aggregated data on blood volume (see Section 2.1.1 and **Supplementary Table 1**), with the assumption that these physiological parameters feature the same dynamics across age groups. Secondly, as the cellular kinetics model was calibrated on data from the first years of life with respect to the observed increases in cell concentrations during early childhood (18), the thymus involution part of the previously developed thymocyte homeostasis sub-model required adaptation (27). To capture changes in thymus function during the first two years of age, thymus wet weight vs. age dependency (**Supplementary Table 4**, **Equation 1**) was taken into account using additional data (see Section 2.1.1 and **Supplementary Table 1**).

As thymus involution and blood volume changes were the only age-dependent processes incorporated in the homeostatic cellular kinetics model, the ability of the developed model to describe age-related changes in cell concentrations was additionally evaluated.

#### 2.2.2 Description of age-dependent changes in homeostasis processes

To capture age-related changes in CD4^+^ T-lymphocyte homeostasis, various functional forms of the parameter age dependency were evaluated. A sequential stepwise approach with forward selection and backward elimination was applied, to identify all relevant age dependencies and build the full age-dependent CD4^+^ T-lymphocyte homeostasis model. The calibration dataset featured cell concentrations vs. age data for 20 model variables (see Section 2.1.1 and **Supplementary Table 1**), which were treated as steady-state values. Age was incorporated in the model as a regressor. Cellular kinetics model parameter values were fixed based on results from the homeostatic model developed in the previous step (see Section 2.2.1). In the forward selection step, four types of empirical functional age dependencies – linear, exponential, hyperbolic and quadratic (**Figure 1B**) – were tested sequentially, on each model parameter. As a result of the iterative forward selection step, the full model with all consistent age functions was built. In the backward elimination step, the selected age dependencies were sequentially removed from the full model, to test whether an age-dependent function would not significantly contribute to data description, once other age dependencies were included in the model. Model selection at each step was based on the identifiability of age-dependent parameters and lower Akaike information criterion (AIC) values. All selected age dependencies were also evaluated in terms of physiological relevance.

#### 2.2.3 Quantitative assessment of CD4^+^ T-lymphocyte homeostasis regulation

To better capture dynamical changes in CD4^+^ T-lymphocyte kinetics and homeostasis in response to specific perturbations (e.g., thymectomy and/or acute infection), empirical age-dependent functions in the model were evaluated and replaced with more physiologically-justified mechanistic dependencies (**Figure 1C**). The hyperbolic dependencies of model parameters vs. cell counts and concentrations were explored and tested sequentially with respect to their physiological meaning. Parameters from non-replaced age dependencies from the previously developed model were estimated alongside with the tested functions. The final age-dependent CD4^+^ T-lymphocyte homeostasis model was selected according to the identifiability of parameters and AIC comparisons vs. the model developed in the previous step (see Section 2.2.2).

### 2.3 Methodology of model development, performance evaluation and validation

Model development and evaluation were conducted according to the current standards of quantitative system pharmacology and physiologically-based mechanistic modeling (55–59). A sequential modular approach was used, both to distinguish homeostatic and age-dependent processes of T-lymphocytes functioning and to reduce the parameter space per optimization procedure. Several parameter values were fixed based on prior knowledge on homeostasis processes (see 2.1.2 Section) (55–59). For the fixed parameter values with non-informative priors, a likelihood function profiling was applied to obtain the best estimates of the model objective function and to evaluate uncertainty. A plausible uncertainty range (95% confidence interval) for each parameter was obtained via profile the likelihood method, by fixing the parameter across a grid of values, re-optimizing the remaining parameters, and retaining values that yielded near-minimal negative log-likelihood while demonstrating practical identifiability. A proportional residual error model was chosen for all model variables with fixed residual error parameter values, on the respective coefficients of variation (CV) of dependent variables, calculated using the calibration dataset. Identifiability of the estimated parameters was assessed via parameter relative standard error (RSE) values, where parameters with RSE greater than 51% were considered unidentifiable (55, 59). Model comparison and selection on the 2^nd^ and 3^rd^ steps during age-dependent model development were based on identifiability analyses, as well as an AIC-based assessment of models; the model featuring the lowest AIC was then selected. To ensure the computed best-fit solution was at a non-improvable minimum, a multi-start parameter optimization procedure was applied, with 15 iterations.

The description of age-related changes in CD4^+^ T-lymphocyte concentrations was assessed via simulations of steady-state values for each dependent variable, at a given age point. According to the sequential model development approach, model predictions accounted for uncertainty only in parameters calibrated at the respective modeling step. Uncertainty in CD4^+^ T-lymphocyte homeostasis or age- or cell count-dependent parameters were included in model simulations, with 1000 sampled parameter sets for simulation. Predicted means with 95% confidence intervals (CIs) were plotted against the respective observed means and 95% CIs of the data. Visual model diagnostics were also performed, by comparing predicted values against observed data and by presenting weighted model residuals against predicted values and respective ages.

Model evaluation and analysis steps included a validation process of age-related changes in cell count representation, simulation of homeostasis perturbations (e.g., thymectomy), and a sensitivity analysis (**Figure 1D**). Along with the representation of age-related changes for specific cell subpopulations, the ability of the model to predict overall immune status with age was assessed using concentrations of total CD4^+^ T-lymphocytes and total memory (CD45RO^+^) CD4^+^ T-lymphocytes in blood and lymphoid tissue (18) as well as absolute counts of total CD4^+^ T-lymphocytes in blood and peripheral organs (60). The accuracy of independent data description was assessed by comparing the observed and predicted 95% CIs for each total CD4^+^ T-lymphocyte population. For each age group (0–1 year of age; 1–18 year of age; 18–40 year of age; >40 year of age), the percentage of datapoints for which the observed and predicted 95% CIs overlapped, or for which the difference between the CI limits did not exceed 30% of the observed values was calculated. Moreover, a full thymectomy condition was simulated, to assess the ability of the model to capture dynamics in T-lymphocyte homeostasis. To simulate such a condition, the number of SP4 thymocytes (**Supplementary Table 4**,**Equation 16**) was set to zero at the time of thymectomy and thereafter. For the dynamic simulation, initial values of the model variables were set to the subsequent steady-state values at the time of thymectomy, per each individual. If, for a given individual, data on T-cell counts prior to thymectomy were available, initial baseline values were corrected by the fraction of the difference between model-predicted cell count for a typical healthy subject and the observed data.

Sensitivity analyses were performed to quantify how a change in model parameter would impact homeostasis of CD4^+^ T-lymphocytes. The partial rank correlation coefficient (PRCC) was used as a measure of global sensitivity (61). Global sensitivity analysis was performed for each dependent variable at select age points (0, 1, 20, 50, 80 years of age), representative of the chosen age groups. Three categories of parameters were used for sensitivity analysis: thymocyte homeostasis; CD4^+^ T-lymphocyte homeostasis; and age- and cell count-dependent parameters (70 parameters in total). Thymocyte homeostasis parameters were sampled from physiologically plausible ranges, as defined in (27), whereas CD4^+^ T-lymphocyte homeostasis and age- and cell count-dependent parameters were sampled from a uniform distribution with ± 25% value ranges, using a latin hypercube sampling method, to conduct the global sensitivity analysis (61). The sample size (i.e., the number of simulation runs) was set to 10,000; sensitivity analysis results were checked for consistency by increasing the sample size. Prior to PRCC calculation, the monotonic relationship between variables and parameter values was assessed. The influence of parameters for which PRCC was greater than or equal to 0.5 in absolute value was considered significant for the corresponding model outputs. To assess pairwise parameter interactions affecting the model-predicted number of CD4^+^ T-lymphocytes, two-parameter iso-line contour plots were computed for selected parameter pairs at fixed ages. For each pair, parameters were sampled from a uniform distribution with ± 25% value ranges on a 2D grid while all remaining parameters were held at their nominal values, and iso-output contour plots were generated to visualize compensation and nonlinear interaction patterns.

### 2.4 Software

Model calibration was performed using the Monolix software (version 2020R1; Lixoft; https://www.simulations-plus.com/software/monolix/monolix/). Model evaluation, validation and sensitivity analyses were performed in the R statistics software (version 4.2.3; packages: rxode2, sensitivity, tidyverse; www.r-project.org). Data digitization was conducted in PlotDigitizer (https://plotdigitizer.com/). Model code is available in the **Supplementary Materials**.

## 3 Results

### 3.1 Model-based description of homeostatic CD4^+^ T-lymphocyte kinetics

The homeostatic CD4^+^ T-lymphocyte cellular kinetics model (**Figure 1A**) calibration results are presented in **Table 1**. A total of 26 parameter values were fixed, and 19 parameters were estimated using meta-analytical generalized estimates on cell concentration data for the first years of life. Age as a regressor in the model was fixed to 0, influencing the age-related changes in thymic function ( $T_{cort}^{max}\left(age\right)$ and $T_{med}^{max}\left(age\right)$) and blood volume ( BV(age)), which captures cell homeostasis for newborns. Each one of the estimated parameters was assessed as identifiable (RSE< 51%) (**Table 1**). For the fixed parameters, values were obtained either from derived physiological experimental estimates (see **Supplementary Table 2** for subsequent derivations) or based on sensitivity and likelihood profiling analyses.

**Table 1:** Homeostatic CD4^+^ T-lymphocyte cellular kinetics model calibration results.

Parameter, units	Description	Parameter Value	RSE, %	Source *
$\mu_{RTE4}$, d^-1^	Death rate of RTE CD4^+^ T-cells	0.0076		*Fixed based on physiological range* *(*30, 31)
$\omega_{RTE4_{bl\_lt}}$, d^-1^	Transition rate of RTE CD4^+^ T-cells from blood to lymphatic tissue	0.217	6.21	Estimated
$\varphi_{RTE4}$, d^-1^	Differentiation rate of RTE to naïve CD4^+^ T-cells	0.0012		*Fixed based on physiological range* *(*30, 32)
$\mu_{N4}$, d^-1^	Death rate of naïve CD4^+^ T-cells	0.000457		*Fixed based on physiological range* *(*32–39)
$\lambda_{N4}$, d^-1^	Proliferation rate of naïve CD4^+^ T-cells	0.0007		*Fixed based on physiological range* *(*12, 32, 35, 40)
$\varphi_{N4}$, d^-1^	Differentiation rate of naïve to activated CD4^+^ T-cells	0.0012	1.01	Estimated
$\omega_{N4_{lt-bl}}$, d^-1^	Transition rate of naïve CD4^+^ T-cells from lymphatic tissue to blood	1.64	6.13	Estimated
$\omega_{N4_{bl-lt}}$, d^-1^	Transition rate of naïve CD4^+^ T-cells from blood to lymphatic tissue	40		*Fixed based on physiological range* *(*43–45)
$\omega_{N4_{bl-git}}$, d^-1^ $\omega_{N4_{bl-lung}}$, d^-1^ $\omega_{N4_{bl-tis}}$, d^-1^ $\omega_{A4_{bl-tis}}$, d^-1^	Transition rate of naïve CD4^+^ T-cells from blood to gastro-intestinal tract/lungs/other peripheral tissues and activated CD4^+^ T-cells from blood to peripheral tissues	0.0003		*Fixed based on sensitivity and likelihood profiling analysis*
$\omega_{N4_{git-bl}}$, d^-1^ $\omega_{N4_{lung-bl}}$, d^-1^	Transition rate of naïve CD4^+^ T-cells from the gastro-intestinal tract/lungs to blood	0.00075	18.63	Estimated
$\lambda_{A4}$, d^-1^	Proliferation rate of activated CD4^+^ T-cells	1.725		*Fixed based on physiological range* *(*41, 85)
$\varphi_{A4}$, d^-1^	Differentiation rate of activated CD4^+^ T-cells	1.727	0.12	Estimated
$\mu_{A4}$, d^-1^	Death rate of activated CD4^+^ T-cells	0.04		*Fixed based on physiological range* *(*46, 47)
$\omega_{A4_{lt-bl}}$, d^-1^	Transition rate of activated CD4^+^ T-cells from lymphatic tissue to blood	4.06	10.17	Estimated
$\omega_{A4_{bl-lt}}$, d^-1^	Transition rate of activated CD4^+^ T-cells from blood to lymphatic tissue	40		*Fixed based on physiological range* *(*43–45)
$f_{4}$, -	Fraction of activated CD4^+^ T-cells, differentiated to central-memory CD4^+^ T-cells	0.4		*Fixed based on physiological range* *(*15)
$T_{A4_{max}}$, cells	Maximal carrying capacity of activated CD4^+^ T-cells proliferation	10^13^		*Fixed based on physiological range* *(*18, 60, 85)
$\lambda_{CM4}$, d^-1^	Proliferation rate of central-memory CD4^+^ T-cells	0.0391		*Fixed based on physiological range* *(*15, 35, 36, 40)
$\mu_{CM4}$, d^-1^	Death rate of central-memory CD4^+^ T-cells	0.041		*Fixed based on physiological range* *(*33, 35, 36, 38, 42)
$\varphi_{CM4}$, d^-1^	Differentiation rate of central-memory to effector-memory CD4^+^ T-cells	0.111		*Fixed based on physiological range* *(*15, 38)
$\omega_{CM4_{lt-bl}}$, d^-1^	Transition rate of central-memory CD4^+^ T-cells from lymphatic tissue to blood	0.334	9.39	Estimated
$\omega_{CM4_{bl-lt}}$, d^-1^	Transition rate of central-memory CD4^+^ T-cells from blood to lymphatic tissue	10		*Fixed based on physiological range* *(*43, 44)
$\omega_{CM4_{bl-git}}$, d^-1^ $\omega_{CM4_{bl-lung}}$, d^-1^ $\omega_{CM4_{bl-tis}}$, d^-1^	Transition rate of central-memory CD4^+^ T-cells from blood to the gastro-intestinal tract/lungs/other peripheral tissues	0.12		*Fixed based on sensitivity and likelihood profiling analysis*
$\omega_{CM4_{git-bl}}$, d^-1^ $\omega_{CM4_{lung-bl}}$, d^-1^	Transition rate of central-memory CD4^+^ T-cells from the gastro-intestinal tract to blood	0.09	19.44	Estimated
$\lambda_{EM4}$, d^-1^	Proliferation rate of effector-memory CD4^+^ T-cells	0.042		*Fixed based on physiological range* *(*15, 35, 36, 40)
$\mu_{EM4}$, d^-1^	Death rate of effector-memory CD4^+^ T-cells	0.11		*Fixed based on physiological range* *(*33, 35, 36, 38, 42)
$\varphi_{EM4}$, d^-1^	Differentiation rate of effector-memory to effector CD4^+^ T-cells	0.035	33.02	Estimated
$\omega_{EM4_{lt-bl}}$, d^-1^	Transition rate of effector-memory CD4^+^ T-cells from lymphatic tissue to blood	0.035		*Fixed based on physiological range* *(*43, 44, 48, 49)
$\omega_{EM4_{bl-git}}$, d^-1^ $\omega_{EM4_{bl-lung}}$, d^-1^ $\omega_{EM4_{bl-tis}}$, d^-1^	Transition rate of effector-memory CD4^+^ T-cells from blood to the gastro-intestinal tract/lungs/other peripheral tissues	0.882	11.95	Estimated
$\omega_{EM4_{git-bl}}$, d^-1^ $\omega_{EM4_{lung-bl}}$, d^-1^	Transition rate of effector-memory CD4^+^ T-cells from the gastro-intestinal tract/lungs to blood	0.04		*Fixed based on physiological range* *(*48, 86)
$\mu_{EFF4}$, d^-1^	Death rate of effector CD4^+^ T-cells	0.87	12.16	Estimated
$\omega_{EFF4_{lt-bl}}$, d^-1^	Transition rate of effector CD4^+^ T-cells from lymphatic tissue to blood	0.253	23.05	Estimated
$\omega_{EFF4_{bl-git}}$, d^-1^ $\omega_{EFF4_{bl-lung}}$, d^-^ $\omega_{EFF4_{bl-tis}}$, d^-1^	Transition rate of effector CD4^+^ T-cells from blood to the gastro-intestinal tract/lungs/other peripheral tissues	0.087	23.32	Estimated

* For fixed parameters see **Supplementary Table 2** for the respective physiological range

The observed and predicted steady-state mean values with respective CIs are presented in **Supplementary Figure 1**. Since cell concentration data from the youngest age group were used for calibration purposes, the corresponding age range varied from the [0; 0.25] years for naïve, central-memory and effector-memory cell blood concentrations, to [30; 50] years for the percentage of activated cells in lymphoid tissue. The age ranges used for model calibration are also depicted in **Supplementary Figure 1**. The model adequately described the data for nearly all CD4^+^ T-lymphocyte subpopulations, despite its limited ability to capture the % of activated cells in lymphoid tissue. Since data were only available for the [30; 50] age group for this variable, and while the model was considered to reflect cellular homeostasis for newborns and the first years of life, the overall results of the model were considered reliable. Model parameters for the thymus wet weight and blood volume age dependencies calibrated separately are presented in **Supplementary Table 5**. To validate model results, concentrations of total T-lymphocyte subsets were simulated. The predicted means with 95% CIs of total CD4^+^ and total memory CD4^+^ CD45RO^+^ T-lymphocyte blood cell concentration were 2402 [2176; 2651] and 300 [263; 334] cells/μL, respectively. These values map well to the observed values for the 0-0.25 age group: 2215.23 [2023.43; 2407.03] and 393.33 [316.85; 469.82] cells/μL, respectively.

The model description of age-related dynamics is presented in **Supplementary Figure 2**. Model diagnostic plots are shown in **Supplementary Figure 3**. In general, the model was not able to fully capture long-term age-related changes in CD4^+^ T-lymphocyte concentrations. Blood concentrations of less differentiated cell subsets (RTE, naïve, activated T-cells) and effector cells decline with age - which is adequately captured in the homeostatic cellular kinetics model (**Supplementary Figure 2A**). Nevertheless, the model does not reproduce age-related changes for memory subsets (central-memory and effector-memory) since, for these subpopulations, blood concentrations do not decrease with age (**Supplementary Figure 2A**). Steady-state cell percentages in organs do not change over age, as no age-dependent variations in cellular kinetic processes were incorporated in the model (**Supplementary Figures 2B–D**). However, the developed cellular kinetics model described the increase in blood cell concentrations for the first year of life (**Supplementary Figures 2A**). Since the model included age dependencies only at the level of the thymus and blood volume, it can be stated that the initial increase in T-lymphocytes and their expansion are primarily due to thymus production.

### 3.2 Accounting for the influence of age on CD4^+^ T-lymphocyte homeostasis

Age-dependent changes in CD4^+^ T-lymphocyte homeostasis processes were described via 13 empirical age-functions (**Figure 1B**). Naïve ( $\lambda_{N4}\left(age\right)$) and activated ( $\lambda_{A4}\left(age\right)$) T-cell proliferation rate, effector-memory T-cell transfer from lungs to blood ( $\omega_{EM4_{lung-bl}}\left(age\right)$), and effector cell transfer from blood to peripheral organs ( $\omega_{EFF4_{bl-git}}\left(age\right)$, $\omega_{EFF4_{bl-lung}}\left(age\right)$, $\omega_{EFF4_{bl-tis}}\left(age\right)$) were found to increase with age. RTE T-cell death rate ( $\mu_{RTE4}\left(age\right)$) and transfer rate from blood to lymphoid tissue ( $\omega_{RTE4_{bl-lt}}\left(age\right)$), differentiation rates of central-memory to effector-memory ( $\varphi_{CM4}\left(age\right)$) and effector-memory to effector ( $\varphi_{EM4}\left(age\right)$) T-cells, and transfer rates from lymphoid tissues to blood of activated ( $\omega_{A4_{lt-bl}}\left(age\right)$), central-memory ( $\omega_{CM4_{lt-bl}}\left(age\right)$) and effector ( $\omega_{EFF4_{lt-bl}}\left(age\right)$) T-cells exhibited decreasing patterns with age. Hyperbolic functions were found to be the most adequate way to capture age-related changes. The schematic structure of the mathematical model with highlighted age-dependent processes is shown in **Supplementary Figure 4**.

The respective formulations and parameter estimates are presented in **Supplementary Tables 6** and **7**, respectively. Overall, 15 and 8 parameter values were estimated and fixed, respectively, during the model calibration process. In the corresponding hyperbolic functions, the Hill coefficients for naïve CD4^+^ T-cell proliferation rate ( $\lambda_{N4}\left(age\right)$) and central-memory CD4^+^ T-cell differentiation rate ( $\varphi_{CM4}\left(age\right)$) age dependencies were fixed to 10 and 3, respectively, to describe the shift in age-related changes in these processes (**Supplementary Table 7**, **Equations 2**, **5** in **Supplementary Table 6**). For decreasing age-functions, such as $\omega_{RTE4_{bl-lt}}\left(age\right)$, $\varphi_{CM4}\left(age\right)$, $\varphi_{EM4}\left(age\right)$, $\omega_{A4_{lt-bl}}\left(age\right)$, $\omega_{CM4_{lt-bl}}\left(age\right)$ and $\omega_{EFF4_{lt-bl}}\left(age\right)$ (**Equations 4**–**6**, **11**–**13** in **Supplementary Table 6**, respectively), parameter values representing the maximal relative decreases in the respective homeostatic processes were set to 1 (100% decrease), to reduce the dimensionality of the model parameter space. The validity of the proposed assumption was tested by sensitivity analysis, assessing the impact of variation in fixed parameter values on model outputs. Age-related changes of the effector T-cell migration rate were assumed to have the same dynamics across different organs ( $\omega_{EFF4_{bl-git}}\left(age\right)$, $\omega_{EFF4_{bl-lung}}\left(age\right)$, $\omega_{EFF4_{bl-tis}}\left(age\right)$; **Equations 8**–**10** in **Supplementary Table 6**). Also, it was assumed that age-related changes in the homeostasis of effector-memory ( $\varphi_{EM4}\left(age\right)$, $\omega_{EM4_{lung-bl}}\left(age\right)$) and effector ( $\omega_{EFF4_{bl-git}}\left(age\right)$, $\omega_{EFF4_{bl-lung}}\left(age\right)$, $\omega_{EFF4_{bl-tis}}\left(age\right)$) T-cells begin around the same age, which is reflected by fitting one parameter corresponding to an age of 50% increase in these processes ( $age_{50}$; **Equations 6**–**10** in **Supplementary Table 6**). An evaluation of separate parameters for these dependencies led to non-identifiability issues for these parameters, due to a larger parameter space. A graphical representation of each age function can be found in **Supplementary Figure 5**. The model selection process for the backward elimination step is captured in **Supplementary Table 8**.

**Figure 3:**
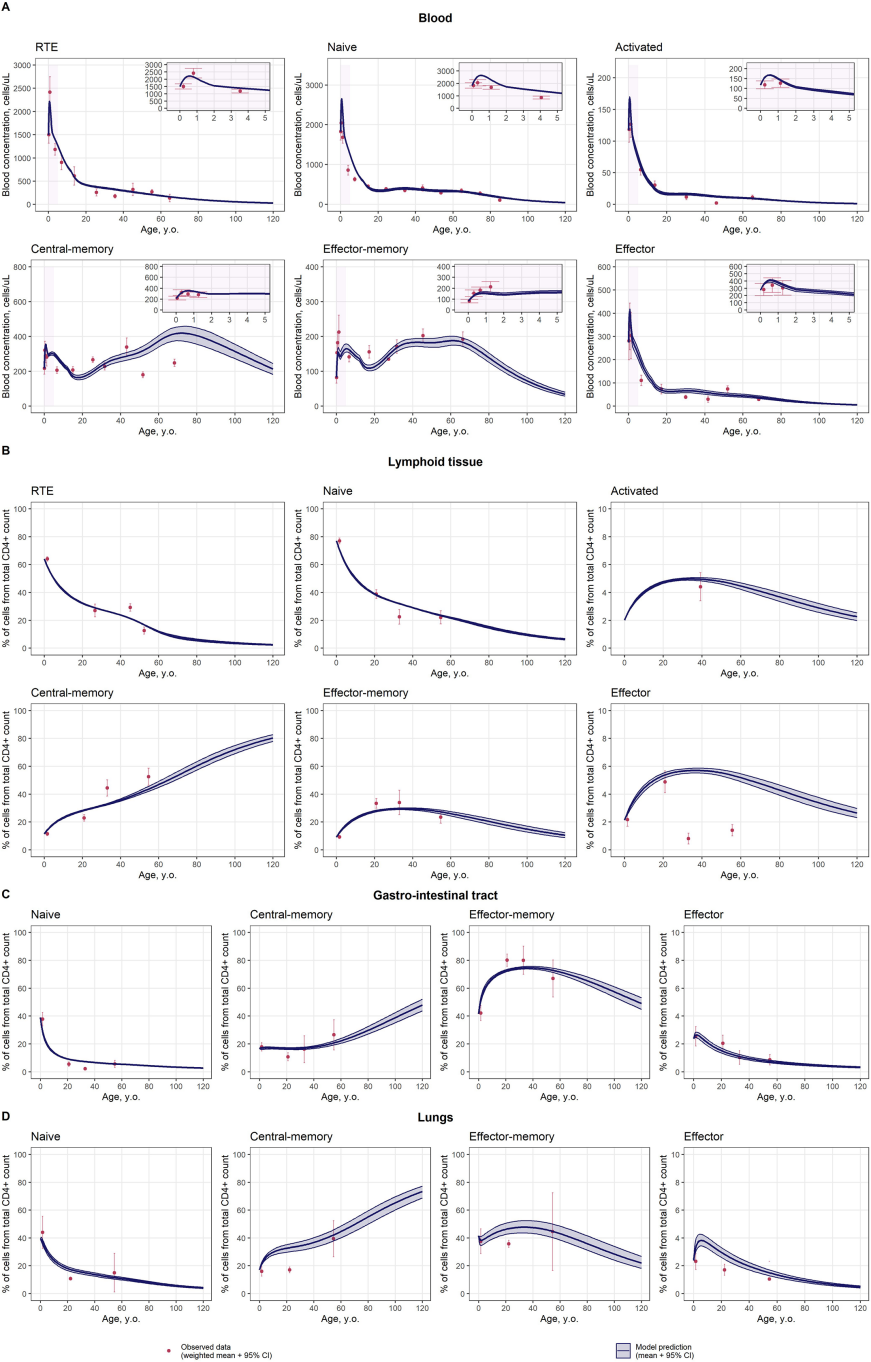
Description of age-related cell dynamics, based on the homeostatic CD4^+^ T-lymphocyte cellular kinetics model with age effects included: Blood **(A)**, Lymphoid tissue **(B)**, Gastro-intestinal tract **(C)**, and Lungs **(D)**. Red dots represent observed data as meta-analytical weighted averages with 95% CIs; blue solid lines with shaded area represent predicted means with 95% CIs; purple shaded areas represent data description for neonates, infants and toddlers (0 to 5 years of age). For improved visualization, the percentage of effector cells is presented for the 0-10% range only, while the 0-100% range is shown for other cell subpopulations.

A description of age-related dynamics, based on the age-dependent CD4^+^ T-lymphocyte homeostasis model is presented in **Figure 3**. The complex nature of changes in cell concentrations in blood and organs with age were well captured, as compared to the homeostatic cellular kinetics model without the incorporation of such age effects, indicating the necessity of the proposed age functions. Diagnostic plots presented in **Supplementary Figure 6** also indicated an accurate age dynamics description, with a good correspondence between observed and predicted values.

Each of the proposed age functions carries a plausible, biological explanation. The increase in activated T-cell proliferation $\lambda_{A4}\left(age\right)$ (**Supplementary Figure 5A**) features a saturation process, indicative of the evolution of clonal expansion in response to antigen stimulation in adulthood and its maintenance for the rest of human life. Homeostasis was most sensitive to the presence of this age dependence, according to the difference in likelihood values for the data described by model, with and without the $\lambda_{A4}\left(age\right)$ function (**Supplementary Table 8**). The increase in clonal expansion efficiency was essential to ensure a sufficient supply of memory cells, with their subsequent accumulation throughout human life (**Figure 3**; central-memory and effector-memory cells). For naïve T-cells, the observed change in the proliferation rate $\lambda_{N4}\left(age\right)$ (**Supplementary Figure 5B**) ~50 years of age was consistent with the adaptation of naïve T-cells to a decreased thymic output. The same reasoning may apply to explain the increase in the survival rate of RTE cells, $\mu_{RTE4}\left(age\right)$ (**Supplementary Figure 5C**).

It is known that age-related changes from lesser to more differentiated T-lymphocytes are observed (10), which is consistent with cell concentrations simulated in both blood and other organs (**Figure 3**) (18). One possible mechanism for this phenomenon may relate to age-dependent changes of differentiation rates of memory cells, which would correspond to immune response activation upon antigen re-exposure (**Supplementary Figures 5E, F**). Since the developed model included distinct subpopulations of human memory T-cells (i.e., central-memory and effector-memory), it enabled the examination of heterogeneity in age-related changes across memory subsets. Differences in age dynamics were found among memory cell subpopulations: the percentage of effector-memory T-cells in lymphoid tissue tended to decrease after 40 years of life, while the dynamics of central-memory T-cells exhibited the opposite pattern (**Figure 3B**). The same differences were observed for cell percentages in the gastro-intestinal tract and lungs (**Figures 3C, D**). Such dynamics can be explained and described by an age-related decrease in the differentiation rate of central-memory to effector-memory cells $\varphi_{CM4}\left(age\right)$ (**Supplementary Figure 5E**), which may lead to an accumulation of the former and a decline of the latter. A decrease in further differentiation from effector-memory to effector CD4^+^ T-cells ( $\varphi_{EM4}\left(age\right)$) was also observed (**Supplementary Figure 5F**), indicative of age-related changes in the immune system ability to respond to a secondary antigen exposure.

Age-related changes were also found with respect to transition rates between organs. Since the transfer rates to and from the gastro-intestinal tract were assumed to be equal to the respective rates for lungs (cell percentages for each of the cell subpopulations were comparable in newborns between these organs), a different age-related cell dynamics pattern was observed for the effector-memory subset. In particular, the decrease in the percentage of effector-memory T-cells, which was observed in the gastro-intestinal tract, was not observed in the lungs (**Figures 3C, D**). Hence, an increasing saturation function for the transfer rate from lungs to blood, $\omega_{EM4_{lung-bl}}\left(age\right)$ (**Supplementary Figure 5G**), was proposed for effector-memory cells, to describe this difference. An opposing age function was introduced for activated, central-memory and effector T-cell transfer rates, from lymphoid tissue to blood (**Supplementary Figures 5I-K**). The transition of effector cells to peripheral tissues, as terminal action sites, were also described by increasing saturation functions with age ( $\omega_{EFF4_{bl-git}}\left(age\right),\;\omega_{EFF4_{bl-lung}}\left(age\right),\;\omega_{EFF4_{bl-tis}}\left(age\right)$, **Supplementary Figure 5H**). These dependencies delineated a gradual increase in the rate of dissemination of effector cells and the time of onset of the immune response up to adulthood (0–20 years), in connection with the development of organ systems. Incorporation in the model of a decreasing age function for the RTE T-cell transfer rate from blood to lymphoid organs, $\omega_{RTE4_{bl-lt}}\left(age\right)$ (**Supplementary Figure 5D**), enabled a better description of cell concentrations in blood. Since there was no proliferation of RTE cells included in the model structure, this age-dependent process captured not only age-related changes in peripheral transitions of RTE cells, but also the increase in proliferative activity of these cells and in export rates from the thymus, due to thymic involution compensation.

### 3.3 Incorporation of an intercellular regulation on age-dependent CD4^+^ T-lymphocyte homeostasis

Empirical, age-dependent functions for homeostatic processes enabled an accurate description of the age-dependent homeostasis of CD4^+^ T-lymphocytes. To evaluate a reciprocal cell influence on such homeostasis, age-dependent functions were tested by replacing them with negative or positive feedback functions, depending on T-cell concentrations or cell counts (**Figure 1C**). This exploratory analysis on potential cell count impact was performed by plotting the homeostatic process vs. cells counts or concentrations of precursor cells, using the homeostatic model with the age effect being incorporated, as described in the previous step (see Section 3.2). This analysis revealed that two age-dependent homeostatic processes, namely the naïve CD4^+^ T-cell proliferation rate and the RTE CD4^+^ T-cell death rate (**Supplementary Figures 7A, C**; blue curves), depended on the concentration of RTE CD4^+^ cells in blood (**Supplementary Figures 7B, D**; blue curves). The age-increasing proliferation rate of naïve CD4^+^ T cells tended to decrease with CD4^+^ T cell RTE concentrations (**Supplementary Figure 7B**). Conversely, the RTE CD4^+^ T-cell death rate increased with increasing RTE CD4^+^ T cell concentrations, while the relationship with age showed the opposite trend (**Supplementary Figure 7D**). These dependencies could be explained by the immune system adapting to diminishing RTE or naïve cell numbers, which can occur not only during thymic involution, but also under intervention and/or patho-physiological conditions such as thymectomy, thymomas, HIV infection and other conditions influencing the number of antigen-unexposed cells.

For the model to capture these homeostatic relationships, age-dependent functions were tested by replacing them with cell concentration-dependent functions. Upon iterative function replacements and model building, the final model included two RTE CD4^+^ T-cell concentration-dependent hyperbolic functions – for the naïve CD4^+^ T-cell proliferation rate ( $\lambda_{N4}\left(T_{RTE4_{cells\_uL}}^{BL}\right)$) and the RTE CD4^+^ T-cell death rate ( $\mu_{RTE4}\left(T_{RTE4_{cells\_uL}}^{BL}\right)$) – instead of the $\lambda_{N4}\left(age\right)$ and $\mu_{RTE4}\left(age\right)$ functions, respectively. Other age-dependent functions incorporated in the model structure per the previous step (see Section 3.2) remained unchanged. The scheme of the mathematical model with the relevant age and cell count dependencies is shown in **Supplementary Figure 8**. The proposed equations are presented in **Supplementary Table 9**. The baseline values of homeostatic processes for each of the hyperbolic functions ( $\lambda_{N4_{BASE}}=\;\lambda_{N4}\left(T_{RTE4_{cells\_uL}}^{BL}=0\right)$ and $\mu_{RTE4_{BASE}}=\;\mu_{RTE4}\left(T_{RTE4_{cells\_uL}}^{BL}=0\right)$) were formulated according to the previously calibrated parameter values of $\lambda_{N4}$ and $\mu_{RTE4}$. Parameter estimate results are shown in **Supplementary Table 10**. All parameter values for both the cell count-dependent ( $\lambda_{N4}\left(T_{RTE4_{cells\_uL}}^{BL}\right)$ and $\mu_{RTE4}\left(T_{RTE4_{cells\_uL}}^{BL}\right)$) and age-dependent functions ( $\lambda_{A4}\left(age\right)$, $\omega_{RTE4_{bl-lt}}\left(age\right)$, $\varphi_{CM4}\left(age\right)$, $\varphi_{EM4}\left(age\right)$, $\omega_{EM4_{lung-bl}}\left(age\right)$, $\omega_{EFF4_{bl-git}}\left(age\right)$, $\omega_{EFF4_{bl-lung}}\left(age\right)$, $\omega_{EFF4_{bl-tis}}\left(age\right)$, $\omega_{A4_{lt-bl}}\left(age\right)$, $\omega_{CM4_{lt-bl}}\left(age\right)$ and $\omega_{EFF4_{lt-bl}}\left(age\right)$) were identified. According to the exploratory analysis, the Hill coefficients $Nh_{\lambda_{N4}}$ and $Nh_{\mu_{RTE4}}$ for $\lambda_{N4}\left(T_{RTE4_{cells\_uL}}^{BL}\right)$ and $\mu_{RTE4}\left(T_{RTE4_{cells\_uL}}^{BL}\right)$, respectively, were set to 10, in order to describe the sharp shift in the nature of these homeostatic relationships.

A description of cell concentration dynamics with age, as performed by the homeostatic model following the inclusion of these cell influence functions is presented in **Supplementary Figure 9**. Model diagnostics are shown in **Supplementary Figure 10**. The proposed model accurately captured the age dynamics of CD4^+^ T-lymphocytes, along with the age-dependent homeostatic model developed in the previous step (see Section 3.2). Also, the proposed model exhibits relationships of $\lambda_{N4}$ and $\mu_{RTE4}$ with age (**Supplementary Figures 7A, C**; red curves) and with RTE concentration age (**Supplementary Figures 7B, D**; red curves) that are similar to the corresponding relationships in the age-dependent homeostatic model (**Supplementary Figure 7**; blue curves). The AIC values for the cell count-dependent and age-dependent models were, respectively, 4753.23 and 4862.35, indicative of an improved description of the observed data upon including these reciprocal cell influence functions in the model.

The overall performance of the homeostatic CD4^+^ T-lymphocyte cellular kinetic model at each stage of development in reproducing observed CD4^+^ T-lymphocyte subpopulation dynamics is presented in **Table 2**.

**Table 2:** Model performance summary across CD4^+^ T-lymphocyte subpopulation age-dependent dynamics.

	Model description	Organ	Age range	CD4^+^ T-lymphocyte subpopulation						Model insights
				RTE	Naïve	Activated	Central-memory	Effector-memory	Effector	
**Homeostatic CD4^+^ T-lymphocyte cellular kinetics model**	(1) without age effect on cellular kinetics	Blood (cells/μL)	0–1 y. of age	++	+++	+++	+++	++	+++	Initial expansion and subsequent decline of blood cell concentration are captured mainly through the age-dependent thymic involution component; Memory T-cell accumulation is not reproduced; naïve/activated input is insufficient for homeostasis maintenance; No age-related changes in cell percentages are captured, as no age-dependent variations in cellular kinetic processes were incorporated into the model.
			1–18 y. of age	++	+	+++	+	+/–	+++	
			18–40 y. of age	+++	+	+++	–	–	+++	
			>40 y. of age	+	–	–	–	–	+/–	
		Lymphoid tissue (%)	0–18 y. of age	+++	+++	n/a	+++	+++	+++	
			18–60 y. of age	–	–	++	–	–	+	
		Gastro-intestinal tract (%)	0–18 y. of age	n/a	+++	n/a	+++	+++	+++	
			18–60 y. of age	n/a	–	n/a	++	+/–	+	
		Lungs (%)	0–18 y. of age	n/a	+++	n/a	+++	+++	+++	
			18–60 y. of age	n/a	–	n/a	++	+++	+++	
	(2) with age effect	Blood (cells/μL)	0–1 y. of age	++	++	++	++	+++	+++	An age-shifted increase in naïve T-cell proliferation rate ( $\lambda_{N4}\left(age\right)$) reproduces the slight rise in blood concentration at ~64 years as an adaptation to decreased thymic output; The accumulation of memory T-cells is achieved through an increase and maintenance of a higher activated T-cell proliferation rate ( $\lambda_{A4}\left(age\right)$) compared to newborns; Central-memory concentration peak at ~64 years and effector-memory percentage decline after ~40 years is captured by a decrease in the differentiation rate of central-memory to effector-memory cells ( $\varphi_{CM4}\left(age\right)$).
			1–18 y. of age	++	+	+++	++	++	+	
			18–40 y. of age	+	+++	+++	++	++	++	
			>40 y. of age	+++	++	+	+/–	+++	++	
		Lymphoid tissue (%)	0–18 y. of age	++	++	n/a	++	++	+++	
			18–60 y. of age	++	++	+++	++	++	+/–	
		Gastro-intestinal tract (%)	0–18 y. of age	n/a	++	n/a	+++	++	+++	
			18–60 y. of age	n/a	+/–	n/a	++	++	+++	
		Lungs (%)	0–18 y. of age	n/a	+++	n/a	++	+++	++	
			18–60 y. of age	n/a	+	n/a	+	++	++	
	(3) with age and cellular feedback effects	Blood (cells/μL)	0–1 y. of age	++	++	++	++	+++	+++	An increase in naïve T-cell proliferation rate depending on RTE cells concentration ( $\lambda_{N4}\left(T_{RTE4_{cells\_uL}}^{BL}\right)$) reproduces the rise at ~64 years as an adaptation to decreased thymic output.
			1–18 y. of age	++	+	+++	++	++	+	
			18–40 y. of age	+	+++	+++	++	+++	++	
			>40 y. of age	++	++	+	+/–	+++	+	
		Lymphoid tissue (%)	0–18 y. of age	++	++	n/a	++	++	+++	
			18–60 y. of age	++	++	+++	++	++	+/–	
		Gastro-intestinal tract (%)	0–18 y. of age	n/a	++	n/a	+++	++	+++	
			18–60 y. of age	n/a	+	n/a	++	++	+++	
		Lungs (%)	0–18 y. of age	n/a	+++	n/a	++	+++	++	
			18–60 y. of age	n/a	+	n/a	+	++	++	

Cell phenotypes: RTE: CD45RA^+^CD31^+^; Naïve: CD45RA^+^CD45RO^–^CCR7^+^/CD62L^+^; Activated: CD38^+^HLADR^+^; Central-memory: CD45RA-CD45RO^+^CCR7^+^/CD62L^+^; Effector-memory: CD45RA^–^CD45RO^+^CCR7^–^/CD62L^–^; Effector: CD45RA^+^CD45RO^–^CCR7^–^/CD62L^–^; y. - years; n/a - not applicable;

+++ — complete overlap between observed and predicted 95% CIs (100% of cases); ++ — overlap between observed and predicted 95% CIs in >75% of cases or the absolute difference between observed and predicted 95% CI limits ≤30% of the observed value (100% of cases); + — overlap between observed and predicted 95% confidence intervals in >50% of cases or the difference between observed and predicted 95% CI limits ≤30% of the observed value in >50% of cases; +/– — overlap between observed and predicted 95% CIs in >20% of cases or the difference between observed and predicted 95% CI limits ≤30% of the observed value in >20% of cases; – — overlap between observed and predicted 95% CIs in<20% of cases and the difference between observed and predicted 95% CI limits ≤30% of the observed value in<20% of cases within the selected age range.

Age-related cell dynamic representation results are presented in **Supplementary Figure 2**, **Figure 2** and **Supplementary Figure 9** for homeostatic CD4^+^ T-lymphocyte cellular kinetics model (1) (without age effect on cellular kinetics), (2) (with age effect) and (3) (with age and cellular feedback effects), respectively.

### 3.4 Model evaluation and analysis

#### 3.4.1 Model validation on external data

**Figure 4:**
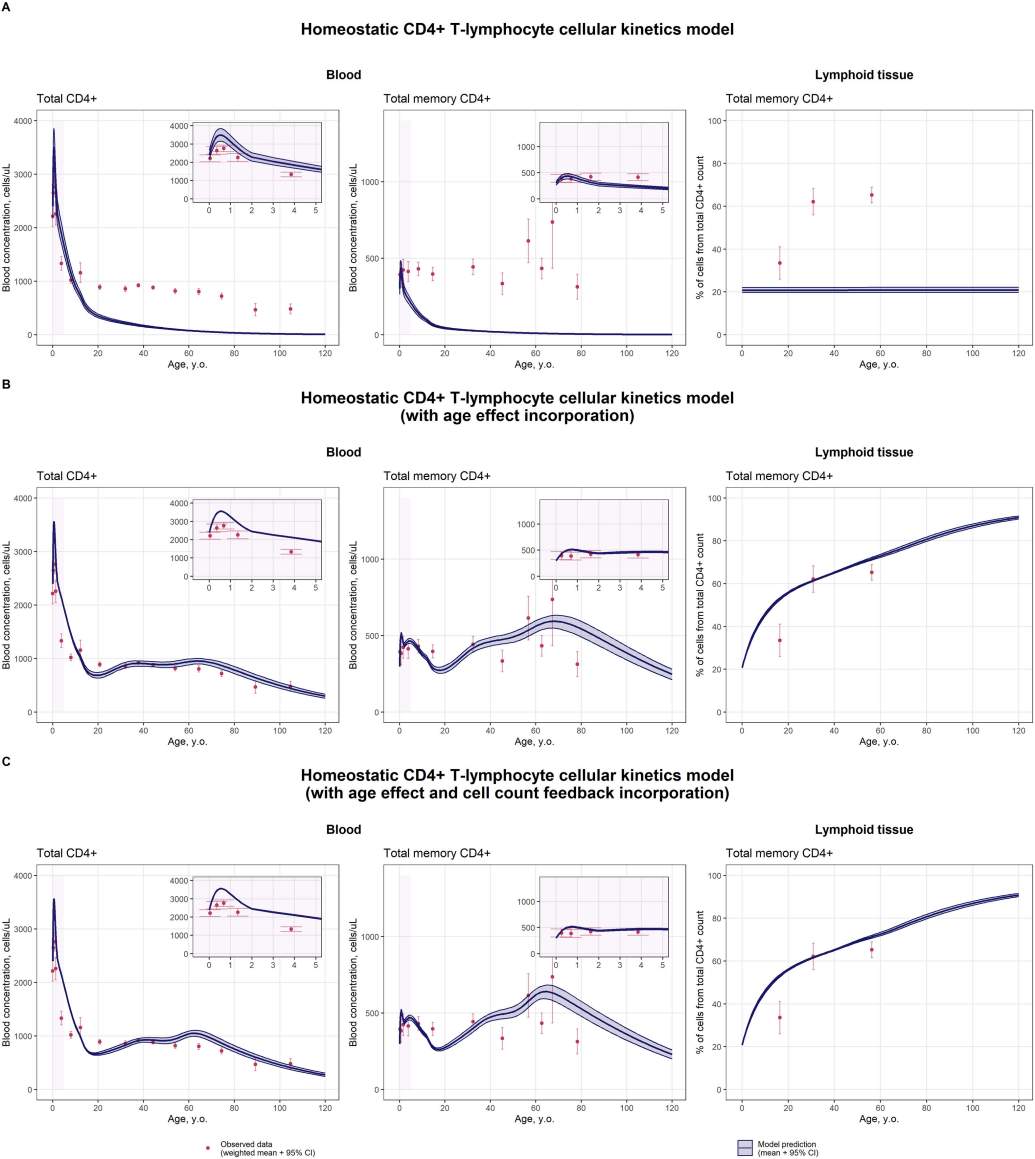
Description of age-related cell dynamics for total CD4^+^ and total memory CD4^+^ CD45RO^+^ T-lymphocyte concentrations in blood and lymphoid tissue by the homeostatic CD4^+^ T-lymphocyte cellular kinetics model (**(A)** – without age effect on cellular kinetics, **(B)** – with age-dependent function inclusion; **(C)** – with age- and cell count-dependent function inclusion) (red dots represent observed data as meta-analytical weighted averages with 95% CIs; blue solid lines with shaded area represent predicted means with 95% CIs; purple shaded areas represent data description for neonates, infants and toddlers (0 to 5 years of age)).

A model evaluation analysis was conducted, to test the hypotheses underlying the model structure and to assess the generalizability of the system (**Figure 1D**). An independent dataset of total CD4^+^ and total memory CD4^+^ CD45RO^+^ T-lymphocyte concentrations was used to evaluate the model ability to describe the overall immune homeostasis (18). Model validation results per each model development step are presented in **Figure 4**, **Table 3**. With respect to specific CD4^+^ T-cell subpopulations (**Supplementary Figure 2**), age-related changes in total CD4^+^ T-lymphocytes were not described by the basic cellular kinetics model (observed and predicted CIs overlapped in 12.9% of cases; **Figure 4A**, **Table 3**). Nevertheless, the observed increase in blood concentrations in the first years of life was well captured by the model, indicating the pivotal role of thymic output in the overall immune system functioning in early childhood. Incorporation of age-dependent and cell-dependent functions drastically improved the description of the data (observed and predicted CIs overlapped in 45.2% of cases; **Figures 4B, C**, **Table 3**). Inclusion of cellular feedback influences yielded more precise total memory cell blood concentration predictions for the 50–70 years-of-age group (**Figure 4C**), in instances where an increase in memory cells was observed, as compared to the model with inclusion of age effects only (**Figure 4B**) (percentage differences between observed and predicted mean values were 22.3% and 21.9% for model with age and cellular feedback effects and model with age effect, respectively; **Table 3**). The age dynamics of memory cells in lymphoid tissues were also well described by both models (**Figures 4B, C**).

**Table 3:** Summary of validation and model evaluation results per each step of the homeostatic CD4^+^ T-lymphocyte cellular kinetics model development.

CD4^+^ T-lymphocyte subpopulation	Age window	Expected trend	Homeostatic CD4^+^ T-lymphocyte cellular kinetics model		
			(1) without age effect on cellular kinetics	(2) with age effect	(3) with age and cellular feedback effects
Model validation results*					
Total CD4^+^ T-cells in blood (cells/μL)	0–1 y. of age	Marked increase (0–1 years)	++	++	++
	1–18 y. of age	Steep decline	++	+	+
			(via the age-dependent thymic involution component)		
	18–40 y. of age	Stable level	–	++	++
				(via an increase and maintenance of a higher activated T-cell proliferation rate ( $\lambda_{A4}\left(age\right)$) compared to newborns)	
	>40 y. of age	Slight increase followed by a gradual decline	–	++ (an age-shifted increase in proliferation rate ( $\lambda_{N4}\left(age\right)$) reproduces the slight rise at ~64 years as an adaptation to decreased thymic output)	++ (an increase in proliferation rate depending on RTE cells concentration ( $\lambda_{N4}\left(T_{RTE4_{cells\_uL}}^{BL}\right)$) reproduces the rise at ~64 years as an adaptation to decreased thymic output; the concentration peak more observable compared to model with age effect included only);
Total memory CD4^+^ T-cells in blood (cells/μL)	0–1 y. of age	Stable level or slight increase	+++	++	++
	1–18 y. of age	Stable level	+	++	++
	18–40 y. of age	Stable level or gradual increase	– (no cell accumulation is observed)	+++	+++
				(via an increase and maintenance of a higher activated T-cell proliferation rate ( $\lambda_{A4}\left(age\right)$) compared to newborns)	
	>40 y. of age	Marked increase followed by a decline	– (no cell accumulation is observed)	+ (an age-shifted increase in naïve cells proliferation rate ( $\lambda_{N4}\left(age\right)$) and a decrease in the differentiation rate of central-memory to effector-memory cells reproduce the rise at ~64 years)	+ (an increase in naïve cells proliferation rate depending on RTE cells concentration ( $\lambda_{N4}\left(T_{RTE4_{cells\_uL}}^{BL}\right)$) and a decrease in the differentiation rate of central-memory to effector-memory cells reproduce the rise at ~64 years; the concentration peak more observable compared to model with age effect included only)
Total memory CD4^+^ T-cells in lymphoid tissue (%)	All age groups	Initial steep increase followed by a gradual rise	+/– (no age-dependent variations in cellular kinetic processes were incorporated in the model)	+	+
				(via an increase and maintenance of a higher activated T-cell proliferation rate ( $\lambda_{A4}\left(age\right)$) compared to newborns)	
Model evaluation results**					
Total CD4^+^ T-cells in blood, lymphoid tissue, gastro-intestinal tract and lungs (cells)	All age groups		A single peak in cell counts is observed during early childhood (around 5 years of age); the age-related cell dynamics closely follow the dynamics of thymic output.	Three phases of age-dependent dynamics are distinguished; each phase exhibits a characteristic rise, peak, and subsequent decline in cell counts: 1) 0–18 years old; 2) 18–50 years old; and 3) >50 years old.	
RTE T-cells in blood, lymphoid tissue, gastro-intestinal tract and lungs (cells)	All age groups			Cell counts increase sharply during the first years of life and remain at high levels until approximately 20–25 years of age; thereafter, cell numbers gradually decline as a result of thymic involution.	
Naive T-cells in blood, lymphoid tissue, gastro-intestinal tract and lungs (cells)	All age groups			Three phases are observed for naïve T cells (1) 0–18 years old; 2) 18–50 years old; and 3) >50 years old): the first peak (at ~4 years old) is driven by the increased thymic output; the second (at ~40 years old) reflects the adaptation of RTE cell survival to reduced thymic output; and the third (at ~64 years old) is associated with the adaptation of naïve cell proliferation to the decreased number of RTE cells.	
Central- and effector-memory T-cells in blood, lymphoid tissue, gastro-intestinal tract and lungs (cells)	All age groups			Three phases are observed for central- and effector-memory T cells, with a gradual increase in concentration up to approximately 64 years of age, followed by a subsequent decline	
Effector T-cells in blood, lymphoid tissue, gastro-intestinal tract and lungs (cells)	All age groups			Effector T cells exhibit age-related dynamics similar to those of naïve T cells	

* Model validation results are presented on **Figure 4**; ** Evaluation of age dynamics of cell counts is shown on **Supplementary Figures 11**-**15**; y. – years; n/a – not available;

+++ — complete overlap between observed and predicted 95% CIs (100% of cases); ++ — overlap between observed and predicted 95% CIs in >75% of cases or the absolute difference between observed and predicted 95% CI limits ≤30% of the observed value (100% of cases); + — overlap between observed and predicted 95% confidence intervals in >50% of cases or the difference between observed and predicted 95% CI limits ≤30% of the observed value in >50% of cases; +/– — overlap between observed and predicted 95% CIs in >20% of cases or the difference between observed and predicted 95% CI limits ≤30% of the observed value in >20% of cases; – — overlap between observed and predicted 95% CIs in<20% of cases and the difference between observed and predicted 95% CI limits ≤30% of the observed value in<20% of cases within the selected age range.

In terms of describing total CD4^+^ T-lymphocyte absolute cell counts in organs (**Supplementary Figure 11**), the model performed well, as compared to estimates of immune cell distributions (60). Estimates of total T-cell counts, as obtained by Sender and colleagues, were 8*10^9^, 3.6*10^11^, 1.7*10^10^ and 1.3*10^10^ cells in, respectively, blood, lymphoid organs, the gastro-intestinal tract and lungs (60). Considering CD4^+^/CD8^+^ ratio estimates of ~2.0 for blood and lymphoid tissue and of ~1.0 for peripheral organs (18), the CD4^+^ T-lymphocyte cell counts were ~ 5.3*10^9^, ~ 2.4*10^11^, ~ 8.5*10^9^ and 6.5*10^9^ cells in, respectively, blood, lymphoid organs, the gastro-intestinal tract and lungs. The maximum CD4^+^ T-lymphocyte cell count predicted by the homeostatic model for these compartments reached, respectively, 5.09*10^9^ (95% CI: [4.85*10^9^; 5.33*10^9^]), 2.02*10^11^ (95% CI: [1.91*10^11^; 2.12*10^11^]), 8.25*10^9^ (95% CI: [7.84*10^9^; 8.65*10^9^]) and 4.14*10^9^ (95% CI: [3.78*10^9^; 4.58*10^9^]) cells (**Supplementary Figure 11C**). The percentage differences between predicted mean of maximum CD4^+^ T-cell count and observed estimates were 4.0%, 16%, 3.0% and 36% for blood, lymphoid organs, the gastro-intestinal tract and lungs, respectively. Overall, these accurate validation results demonstrated the ability of the homeostatic model to capture both specific and total subpopulation age-related changes.

Age-related changes in absolute cell counts in organs, specific to CD4^+^ T-cell subsets, were also fully examined (see **Supplementary Figures 12**-**15**). Three distinct phases can be distinguished in age dynamics for nearly all subpopulations, based on changes in number of cells: 1) 0–18 years old; 2) 18–50 years old; and 3) >50 years old. In a first phase, a sharp increase in cell numbers with subsequent decline was observed for less differentiated CD4^+^ T-cells (RTE and naive), with a maximum being reached at ~4.0 years after birth (**Supplementary Figure 13C** – RTE and Naive). Cell counts for more differentiated counterparts increased gradually and were saturable (**Supplementary Figure 13C** – Central-memory, Effector-memory, and Effector). Such growth in cell numbers would correspond to an increase in thymic output in the first years of life, ‘back-filling’ the human body with immune cells. The second phase was characterized by a gradual cell count expansion, from 18 years of age up to 40 years of age (**Supplementary Figure 13C**). From 40 to 50 years of age, counts of less differentiated cells decreased (**Supplementary Figure 13C****–** RTE and Naïve), while more differentiated subsets remained stable (**Supplementary Figure 13C** – Effector-memory and Effector) or continued to increase (**Supplementary Figure 13C** – Central-memory). Such behavior may be explained by the attainment of a consistently high level of activated cell proliferation ( $\lambda_{A4}\left(age\right)$, **Supplementary Figure 5A**), after 18 years of age, which would contribute to the fact that clonal expansion ( $\lambda_{A4}$) exerts its most significant influence on cell age-dependent homeostasis (see Section 3.4.3 for sensitivity analysis results). For the third stage, an increase in cell counts at ~ 64 years of age was observed for all subpopulations except RTE cells. This may correspond to an adaptation of naïve T-cell proliferation upon diminished thymic output ( $\lambda_{N4}\left(T_{RTE4_{cells\_uL}}^{BL}\right)$, **Supplementary Figures 7A, B)**, which is observed after ~50 years of age. Upon reaching a peak during this third phase, cell numbers declined for the remaining years of life. Similar behaviors of age dynamics across these three phases were observed for blood, the gastro-intestinal tract, and the lungs (**Supplementary Figures 12**, **14**, **15**).

#### 3.4.2 Prediction of effects of homeostasis perturbations

The model’s ability to describe the dynamic behavior of CD4^+^ T-lymphocytes was assessed based on data on total and naïve cell concentrations from patients who underwent complete thymectomy. An exploratory analysis of the collected data is depicted in **Supplementary Figure 16**. Data were grouped according to age at thymectomy procedure, into 6 groups: 1) 0 to 1 month; 2) 1 to 2 months; 3) 2 months to 1.0 year; 4) 1.0 to 1.5 years; 5) 1.5 to 3.0 years; 6) greater than 3 years of age. For patients who were thymectomized early after birth or during infancy (groups 1-3), short-term dynamical changes of total CD4^+^ T-lymphocyte concentrations in blood are presented for the first 5 years of life (**Supplementary Figure 16A**). For the later thymectomy ages (groups 3-6), CD4^+^ naïve T-lymphocyte concentrations in blood are available for the adulthood period of 20–30 years of age, enabling a long-term evaluation of effects incurred by thymectomy (**Supplementary Figure 16B**).

**Figure 5:**
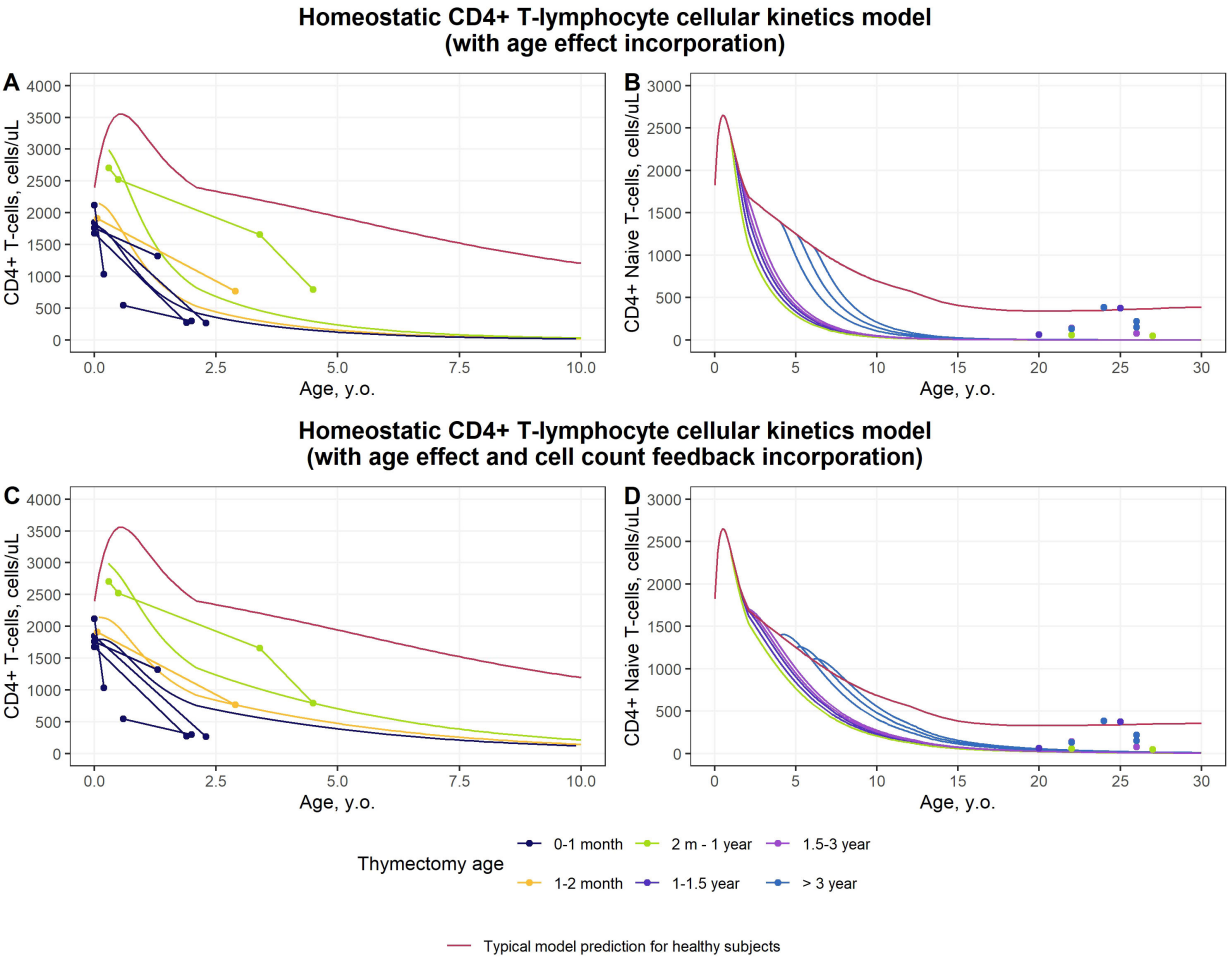
Prediction of total and naïve CD4^+^ T-lymphocyte concentration dynamics after complete thymectomy using the homeostatic model (**(A, B)** – model with inclusion of age-dependent functions; **(C, D)** – model with inclusion of age- and cell count-dependent functions; dots depict individual datapoints from thymectomized subjects; lines illustrate typical model predictions for each data source; color represents group of subjects, according to the age of thymus gland removal; red line indicates typical model-based predictions for healthy, non-thymectomized subjects).

Model-based results following simulated thymectomy conditions are presented in **Figure 5**. After “switching-off” the inflow of mature T-cells into the periphery, the number of CD4^+^ T-cells sharply decreased in the first 2 years of life. The magnitude of cell number decreases fully corresponded to baseline values of cell counts prior to thymectomy. While CD4^+^ T-lymphocyte blood concentrations increased during infancy, as observed from the data (**Supplementary Figure 16A**, red dots) and model predictions (**Figures 5A, C**, red curve), the implications of thymus gland removal directly depended on the age of thymectomy. The short-term dynamic of cell concentrations was well described by the model, with included relationships between homeostatic processes and cell concentrations (**Figure 5C**), as compared to the model which accounted only for an age effect (**Figure 5A**). This exercise supported the evidence that empirical age functions do not allow for a full accounting of strong perturbations in cell homeostasis, over a range of several years. It further indicated the validity of adapting the model structure by incorporating cellular feedback loops. However, the homeostatic model did not fully capture long-term consequences of thymectomy (**Figures 5B, D**), where the observed cell concentrations were close to the values found in healthy non-thymectomized subjects - especially in the case of late-age thymectomy.

#### 3.4.3 Sensitivity analysis results

**Figure 6:**
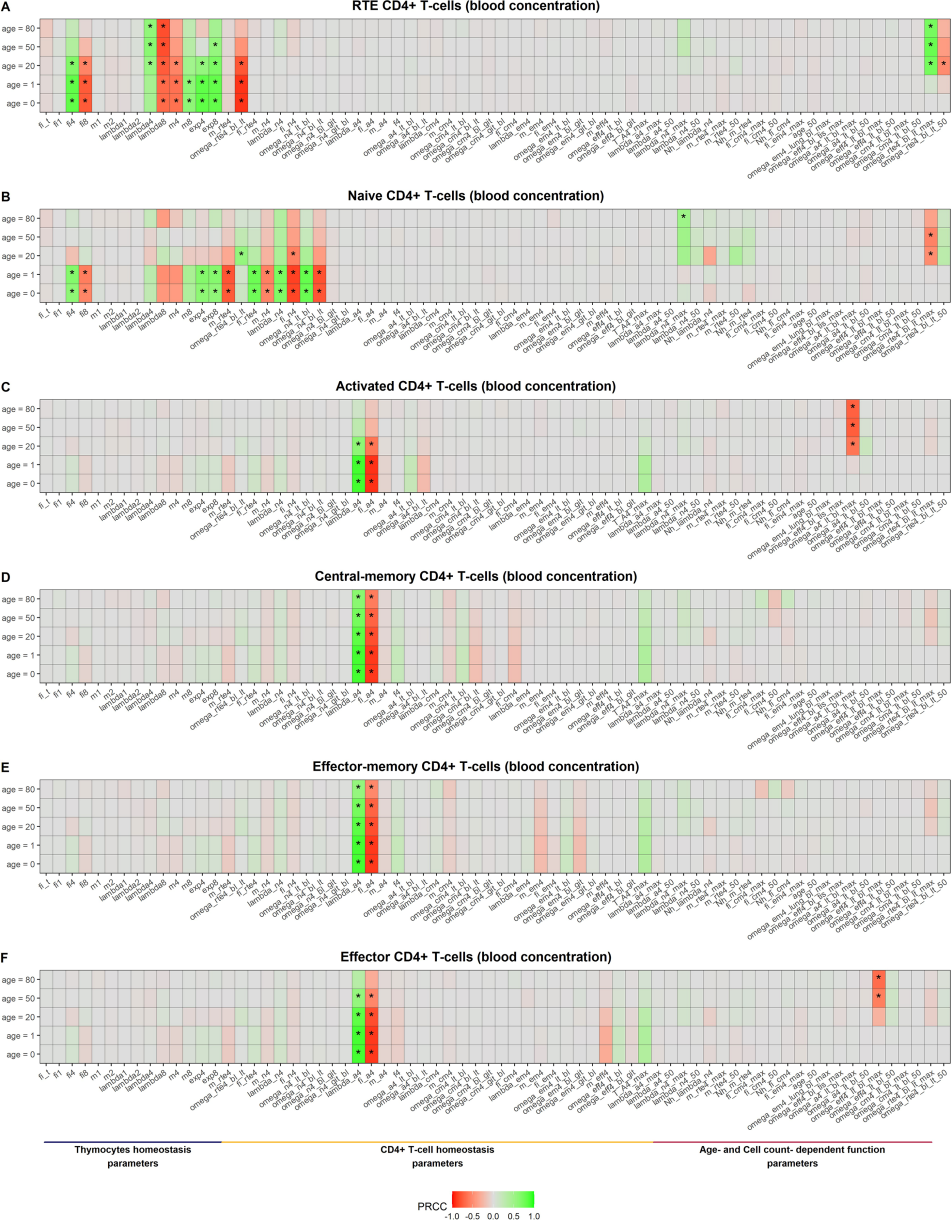
Global sensitivity analysis using a PRCC-based method: Blood concentration of RTE **(A)**, naïve **(B)**, activated **(C)**, central-memory **(D)**, effector-memory **(E)** and effector **(F)** CD4^+^ T-lymphocytes for five age levels (0, 1, 20, 50, 80 years of age) (asterisk (*) indicates |PRCC| ≥ 0.5).

As an integral part of model evaluation, a sensitivity analysis was performed, in order to assess how an arbitrary change in model parameters would impact both cellular kinetics homeostasis and age-related changes. Results from a global sensitivity analysis for blood concentrations of specific CD4^+^ T-cell subsets are presented in **Figure 6**. Homeostasis of lesser differentiated subsets (**Figures 6A, B**) was highly impacted by single-positive thymocyte homeostasis (|PRCC| ≥ 0.5). For RTE CD4^+^ T-lymphocytes, the transition parameter $\omega_{RTE4_{bl\_lt}}$, as well as age-related changes of transition in lymphoid tissue ( $\omega_{RTE4_{bl-lt_{max}}}$ and $\omega_{RTE4_{bl-lt_{50}}}$) had a great impact on the homeostasis of these cells (**Figure 6A**). For naïve CD4^+^ T-lymphocytes, the following homeostatic processes – differentiation from precursor RTE cells, proliferation, death, differentiation to activated cells after antigen encounter and transitions between blood and lymphoid tissue – played a notable role in influencing naïve cell kinetics (**Figure 6B**). Remarkably, the magnitude of the influence changed with age. The impact of single-positive cell differentiation, death and export to the periphery was less observable for adults and elderly, as compared to subjects in their first years of life. The same behavior was revealed for naïve T-cell homeostatic processes. However, thymocyte proliferation displayed the opposite behavior, indicating an increasing dependence of mature T cell homeostasis on thymocyte proliferative activity, as thymic involution progresses. Moreover, the impact of the magnitude of naïve CD4^+^ T-cell proliferation relative decrease ( $\lambda_{N4_{max}\;}^{RTE4}$) became more pronounced in elderly as compared to younger subjects, illustrating the stronger influence of homeostatic proliferation vs. thymic output on immune cell homeostasis at more advanced ages.

The greatest influence on more differentiated CD4^+^ T-lymphocyte subpopulations was exerted by proliferation ( $\lambda_{A4}$) and differentiation ( $\varphi_{A4}$) rates of activated T-cells (**Figures 6D–F**). The proliferation rate of activated CD4^+^ T-lymphocytes, $\lambda_{A4}$, represents the magnitude of clonal expansion, while the differentiation rate, $\varphi_{A4}$, stands for the activity of infectious processes. Results of the sensitivity analysis indicated that antigen exposure throughout human life played a major role in influencing memory and effector cell counts. Lifelong antigen exposure had a much stronger effect on maintaining cell homeostasis than thymic output or the homeostatic regulation of precursor naïve, memory, and effector cells. The significant influence of the maximum relative decrease in the transition rate of effector CD4^+^ T-cells from lymphoid tissue to blood, $\omega_{EFF4_{lt-bl_{max}}}$ on effector cells count became pronounced in elderly subjects (**Figure 6F**). This makes cell circulation properties to be one of the key determinants of effector cells reaching their sites of action. No major differences were found, in the global sensitivity analysis, for cell counts of specific subpopulations in lymphoid tissue, the gastro-intestinal tract, and the lungs (**Supplementary Figures 17**-**19**).

Moreover, a likelihood profiling analysis was used to evaluate the validity of selected model parameter values. Likelihood profiling was conducted for the parameters $\omega_{N4_{bl-git}}$ and $\omega_{CM4_{bl-git}}$, whose values had non-informative priors, to ensure obtaining the best estimate of the objective function for the proposed parameter values (**Supplementary Figure 20**). Fixing the parameter at any value in ranges of [0.0002; 0.01] d^-1^ for $\omega_{N4_{bl-git}}$ and [0.03; 5] d^-1^ for $\omega_{CM4_{bl-git}}$ led to the same likelihood function value, indicating the sensibleness of selected values. This conclusion was also confirmed by the global sensitivity analysis, whereby ± 25% changes in these parameters did not have any significant influence on cellular homeostasis (**Figures 6B, D**).

As the final model included intercellular regulatory mechanisms of naïve T-cell proliferation and RTE cell death adaptation to decreased thymic output, an additional quantification of the contribution of the proposed compensatory mechanisms was performed. Specifically, CD4^+^ T-lymphocyte numbers in lymphoid organs were simulated under three scenarios in which two state-dependent adaptations were selectively removed: (1) no adaptation of naïve T-cell proliferation; (2) no adaptation of the RTE death rate; and (3) neither adaptation, while keeping all other model components unchanged. Simulations of CD4^+^ T-cell dynamics in lymphoid organs showed that removing RTE death adaptation resulted in an ~20% reduction in CD4^+^ T-cell numbers at 20 years of age. Removing naïve proliferation adaptation led to an ~50% reduction in CD4^+^ T-cell numbers at 80 years of age relative to the full model. When both adaptations were disabled simultaneously, CD4^+^ T-cell numbers declined by more than 80%, indicating that these mechanisms jointly contribute to maintaining CD4^+^ T-cell homeostasis over the lifespan (**Supplementary Figure 21**).

The two-parameter response surfaces (contour plots) revealed distinct interaction patterns across the analyzed parameter pairs for CD4^+^ T-lymphocyte numbers in lymphoid organs (**Supplementary Figure 22**). In terms of $\lambda_{N4}$ vs. $\varphi_{EM4}$ (**Supplementary Figure 22B**) interaction, nearly vertical iso-contours indicated that the output was dominated by proliferation of naïve CD4^+^ T-cells, with minor modulation only via the differentiation rate of the effector-memory subset. In contrast, strongly tilted and curved contours in the $\lambda_{N4}$ vs. $\varphi_{CM4}$ plane (**Supplementary Figure 22A**) demonstrated compensatory behavior, where increases in the naïve CD4^+^ T-cell proliferation rate could compensate for changes in the differentiation rate of central-memory cells, to maintain similar CD4^+^ T-cell levels; the curvature suggested non-additive interactions. The $\varphi_{CM4}$ vs. $\varphi_{EM4}\;$ surfaces (**Supplementary Figure 22C**) showed predominantly vertical contours across ages, suggesting that CD4^+^ T-cell numbers in lymphoid tissue are much more sensitive to central-memory cell proliferation vs. effector-memory, with only weak pairwise interactions apparent in restricted regions of the parameter space. For parameter pairs involving $\lambda_{A4}$ (**Supplementary Figures 22D, E**), a threshold-like influence nature was observed, with low CD4^+^ T-cell numbers over a broad region followed by a rapid increase once $\lambda_{A4}$ exceeded a critical range, after which the influence of the paired parameter ( $\varphi_{CM4}$ and $\varphi_{EM4}$) became more apparent. The $\lambda_{A4}$ vs. $\varphi_{A4}\;$ surface showed the clearest compensatory structure, with diagonal iso-line contours consistent with strong parameter interdependence (**Supplementary Figure 22F**). Overall, the qualitative interaction patterns were preserved across ages, while the steepness and curvature of response surfaces varied, indicating age-dependent sensitivity of lymphoid CD4^+^ T-cell numbers to specific parameter combinations.

## 4 Discussion

*In vivo* stable isotope labeling remains a gold standard in the investigation of T-lymphocyte kinetics (5, 12). Comprehensive reviews of T-cell kinetic estimates were provided by two groups, Borghans and de Boer (5) and Macallan et al. (12), summarizing half-life estimates, generation and proliferation rates for total and specific cell subpopulations obtained over two decades, based on deuterium-labelled experimental data analyses. These data were essential in defining experimental estimates of parameters in the present model (see **Supplementary Tables 2**, **3**), enabling validation of the model in its dynamic representation of current knowledge on lymphocyte kinetics. The modeling approaches used to describe labeled data kinetics included predominantly ODE-based models, where kinetic heterogeneity in cell populations was accounted for using a compartmental approach (62). More computationally intense methodologies such as physiologically-based pharmacokinetic modeling (PBPK) (63) and agent-based modeling (ABM) (64) have also been proposed for labeled data description and the derivation of kinetic estimates. However, while these modeling approaches may provide quantitative insights on cell homeostasis, their ability to extrapolate results and to predict cell kinetics under homeostatic alterations is limited. As for the present homeostatic CD4^+^ T-lymphocyte cellular kinetic model, data on steady-state cell counts and concentrations were used, rather than dynamics of labelled cells over limited labeling periods. It provided further options to consider mechanistic aspects of cell behaviors and interactions. The proposed mechanistic model thus incorporated T-lymphocyte kinetics estimates from labelling experiments as well as steady-state information of kinetically heterogenous populations, and enabled predictive simulations of physiologically relevant scenarios.

The developed multiscale homeostatic model included age-related dependencies at various levels: 1) in the thymus compartment, by capturing involution processes ( $T_{cort}^{max}\left(age\right)$ and $T_{med}^{max}\left(age\right)$); 2) on blood volume, representative of organism growth ( BV(age)); 3) on proliferation ( $\lambda_{N4}\left(age\right)$ and $\lambda_{A4}\left(age\right)$) and death ( $\mu_{RTE4}\left(age\right)$) rates of less differentiated T-cell subsets, indicative of adaptation to decreased thymic output and memory cell longevity maintenance; 4) on further differentiation of T-cell subsets ( $\varphi_{CM4}\left(age\right)$ and $\varphi_{EM4}\left(age\right)$), by capturing the kinetic heterogeneity of memory CD4^+^ T-cell subpopulations; and 5) on transitions of circulating cells ( $\omega_{RTE4_{bl-lt}}\left(age\right)$, $\omega_{EFF4_{bl-git}}\left(age\right)$, $\omega_{EFF4_{bl-lung}}\left(age\right)$, $\omega_{EFF4_{bl-tis}}\left(age\right)$, $\omega_{EM4_{lung-bl}}\left(age\right)$, $\omega_{A4_{lt-bl}}\left(age\right)$, $\omega_{CM4_{lt-bl}}\left(age\right)$, $\omega_{EFF4_{lt-bl}}\left(age\right)$)), representative of changes in organ perfusion and permeability throughout life. In terms of capturing, overall, age-related changes in T-lymphocyte numbers, model results were in line with the staging proposed in a recent review by Baliu-Pique et al., distinguishing developmental (0–20 years of age), middle age (20–65 years of age) and older (>65 years of age) stages (13). We observed and quantified similar behavior of age dynamics for total CD4^+^ T-lymphocytes, as well as identified cell count changes within these stages. Specifically, we identified the biphasic nature of CD4^+^ T-cell numbers within the “middle age” stage, with two peaks at ~ 40 and ~ 64 years of age.

Numerous studies have investigated changes in immune homeostasis during healthy aging (65, 66); several studies evaluated such changes using mathematical modeling approaches (67, 68). Firstly, the process of thymic involution has been widely explored and described using various model structures, from the straightforward exponential decay function (69) to mechanistic ODE-based thymocyte population dynamics models accounting for a maximal carrying capacity in thymic niches (27). The data most often used for modeling age-related changes are signal joint TCR excision circles and stable isotope labeling data. Despite the heterogeneity among modeling approaches to thymus homeostasis, the parameter values used in the present thymus sub-model fall within reported ranges, and detailed benchmarking is provided in our previous study (27). One open issue is whether the compensatory homeostatic proliferation in instances of low cell numbers due to, e.g., aging, HIV-infection, or other lymphocytopenia conditions, which is typically observed in rodents, may also hold in humans (70). From a immuno-physiological point of view, this compensatory behavior can be understood in the context of cytokine-regulated T-cell homeostasis. In particular, IL-7/IL-7R signaling is a central regulator of naïve T-cell survival and low-level homeostatic proliferation, and IL-7 availability is shaped by stromal production and T-cell consumption, providing a plausible biological substrate for effective compensation when thymic output declines (29). In the Westera et al. modeling study, no significant peripheral homeostatic compensation in naïve CD4^+^ T-lymphocytes was identified in elderly (66–72 years of age) vs. young healthy subjects (20–25 years of age), despite a decreased thymic output (67). These results contradicted previous experimental evidence of an enhanced percentage of the Ki-67^+^ proliferation marker in naïve CD4^+^ T-cells in elderly subjects with a reduced pool size of immune cells (71). Our modeling results propose that naïve CD4^+^ T-lymphocyte proliferation significantly increases with age. Moreover, the dependency of naïve cell proliferation on RTE cell concentration was taken into account and parameterized. However, our modeling results are still in line with the Westera et al. findings. The point estimates of CD4^+^ naïve T-cell proliferation rates for aged and young subjects were 0.07 and 0.04%*d^-1^, respectively, indicating an aged/young ratio of 1.75 (67). A comparable ratio (1.7) was calculated from the model-based results, with $\lambda_{N4}\left(age=70\right)$ = 0.0012 d^-1^ and $\lambda_{N4}\left(age=23\right)$ = 0.0007 d^-1^. Thymic output was not sufficient to maintain immune cell numbers at their respective levels. The homeostatic CD4^+^ T-lymphocyte cellular kinetics model calibrated on data from the first years of life - which included only thymic involution and blood volume as age-dependent processes - provided a precise description of blood concentration data for the first years of life, while it failed to adequately capture age dynamics for all cell subpopulations. Moreover, the maximal total number of CD4^+^ T-cells in lymphatic tissue predicted by the homeostatic model was almost five-fold less than the number reported in the literature [~5*10^10^ cells vs. ~ 2.4*10^11^ cells (60)]. These model-based findings point to a key role of naïve T-lymphocyte proliferation adaptation and of increased clonal expansion activity, to maintain immune cell homeostasis throughout human life.

The immune cell repertoire investigation using data from patients who underwent thymus gland removal was another rich source of evidence on cell homeostasis (72). Partial or complete thymectomies are surgical procedures motivated by cardiac operations in early childhood, as well as treatment of myasthenia gravis or thymomas. The extent of thymic tissue removal as well as age of thymectomy are principal determinants of T-cell numbers and immune system functionality preservation and restoration. Numerous experimental studies have evaluated numbers of total and naïve CD4^+^ T-lymphocytes coupled with other markers, in thymectomized subjects (72). According to these data, upon complete thymectomy, a substantial decrease in cell concentrations in the first 5 years following the procedure is observed. However, after 20 years, subjects who underwent complete thymus removal age 3 or older exhibited naïve CD4^+^ T-lymphocyte concentrations close to reference values found in non-thymectomized healthy subjects.

The mechanistic model presented here provides an opportunity to investigate the observed cell dynamics and to identify potential regulatory mechanisms which may explain homeostatic adaptation. The homeostatic model of CD4^+^ T-lymphocyte cellular kinetics with incorporation of an age effect was not successful in describing neither short-term nor long-term cell concentration dynamics. This underlines the necessity to take into account homeostatic regulations, without which cell concentrations may drop close to zero, 10 years after a thymectomy procedure. Incorporation of RTE cell concentration feedback on naïve cell proliferation in the model resulted in an adequate description of short-term cell dynamics, which was supported by elevated Ki-67^+^ data in thymectomized subjects, in their first years following thymectomy (73). However, this adaptive mechanism of naïve CD4^+^ T-cell proliferation was not sufficient to capture long-term restoration of immune cell counts; additional regulatory mechanisms needed to be included. While some researchers have reported re-growth of thymic tissue, based on MRI scans, the extent of restored thymopoiesis remains in question (28). Considering the high impact of thymic output on immune homeostasis during the first years of life, both in terms of cell accumulation and the encountering of novel antigens, the present modeling results support the notions of avoiding full thymic removal and, therefore, preserving some minimal amount of thymic tissue during surgery within the first year of life, whenever feasible and appropriate (29, 72). The proposed mechanistic model indeed accounts for the age of thymectomy, and our data analysis showed that one may make more significant inferences from thymectomy data as compared to data of single experimental studies. With this mechanistic inclusion of the thymus gland in the model, simulation-based investigations of various clinically relevant scenarios, such as the extent of thymectomy or gradual thymic re-growth features, as well as other perturbations, become feasible.

In addition to the homeostatic adaptation to declining cell numbers, an important complementary aspect of immune aging is the system’s adaptation to increasing antigenic load and, consequently, to periods of expanded T-cell numbers. The proposed model already captures several key nonlinear homeostatic constraints, including growth control of thymocyte subpopulations, limited peripheral clonal expansion, and an RTE-dependent feedback affecting RTE death and naïve proliferation. At the same time, the current formulation is focused on a baseline, age-dependent homeostasis and does not explicitly consider additional regulatory mechanisms such as cytokine-modulated survival or activation-induced cell death, which are expected to become particularly relevant under strong immune stimulation or infection. Incorporating these processes is a natural extension of the presented framework and would require coupling the homeostatic module to explicit antigen- or infection-driven dynamics. In addition to expanding the proposed physiology-based model to describe infection-driven lymphocyte dynamics, the developed model may also be used in vaccination studies. By providing age-specific baselines for naïve precursor availability and memory maintenance in blood and lymphoid organs, it offers a mechanistic scaffold for comparing vaccine responses across the lifespan. To extend the model for vaccination studies, an antigen-driven activation module should be added; adjuvant effects may be implemented via parameter changes affecting activation, clonal expansion, differentiation to memory, and survival of CD4^+^ T cells. Such an extension would enable an *in silico* evaluation of the heterogeneity of age-specific response magnitude and durability, and support optimization of vaccine dose and scheduling strategies.

In parallel, thymic involution was represented through an effective age-dependent reduction in the maximum carrying capacity of the thymic cortex and medulla. This parameterization was informed by empirical observations of age-associated changes in thymus weight and the decline of the true thymic epithelial space (TES), providing a physiologically grounded description at the level of organ function. The biological basis of TES loss is consistent with age-related remodeling of thymic stromal cells, supported by transcriptomic and single-cell studies reporting broad shifts in TES programs and implicating molecular and endocrine regulators (e.g., FGF21, lamin-B1, and sex steroid hormones) (74, 75). Because these drivers were not modeled explicitly, the proposed framework is not designed to predict the effects of targeted hormonal or epigenetic interventions directly; instead, their aggregate influence is reflected indirectly in the inferred age-dependent capacity functions. Heterogeneity of memory T-cells homeostasis and aging subsets represent another recurring question in immunology research. Modeling research based on deuterated glucose labeling data, as proposed by Macallan et al., indicated differences in turnover rates between CD45RO^+^CCR7^+^ central-memory and CD45RO^+^CCR7^–^ effector-memory CD4^+^ T-cells, with higher proliferation rates for the latter (36). Moreover, studies in mice, as performed by Gossel et al., highlighted both 1) kinetic heterogeneity in memory subsets; and 2) longevity dependence on the recruitment of naïve cells into the memory compartment (15). The first aspect has been captured in the present model as central-memory $T_{CM4}$ and effector-memory $T_{EM4}$ CD4^+^ T-cells, with distinct turnover rates, according to the proposed values of proliferation and differentiation parameters. The second aspect was supported by a sensitivity analysis, which identified activated cell homeostasis as the most impactful process on the maintenance of memory cells and the point estimates of differentiation rates to and from the central-memory subset. Age-related changes have also been observed in memory T-cell homeostasis. The full memory CD4^+^ T-cell subpopulation tends to accumulate throughout human life (65). The same can be observed from the calibration dataset used in the present model: an accumulation of central-memory and effector-memory cells detected both in blood and peripheral tissues (18). However, differences between central- and effector-memory cells can be made based on the age dynamics data, where central-memory (CM) cell percentages increased throughout life, while effector-memory (EM) cell percentages started to decline after ~40 years of age. In the present model, such age-related changes in CM and EM cell homeostasis were captured by decreasing age functions for CM and EM differentiation rates ( $\varphi_{CM4}\left(age\right)$ and $\varphi_{EM4}\left(age\right)$). A plausible explanation for the decline in $\varphi_{EM4}$ may relate to the exhaustion and/or senescence of cell accumulation, due to chronic infections during aging (14), which, when coupled with a higher turnover of EM cells, may lead to a decrease in cell counts. The immunity study in mouse, by Gossel et al., showed a diminished replacement rate of EM CD4^+^ T-cells in older adult mice, as compared to younger animals (15). The authors provided two equally plausible explanations for this phenomenon: the existence of resistant EM CD4^+^ T cells; and decreased differentiation from the naïve pool. Since the present model was designed to capture immune system homeostasis, which includes experience in encountering foreign antigens over a lifespan, and while the Gossel et al. experiments were conducted in non-infected mice, the $\varphi_{CM4}\left(age\right)$ dependency is reflective of EM cells decline.

In terms of heterogeneity in memory cell subsets, one limitation of the present model may reside in the fact that stem cell-like memory T-cells (SCM) were not explicitly represented in the model structure. SCM cells are a long-lived memory cell subpopulation that exhibits higher self-renewal properties than other memory subsets, a multipotent differentiation nature, and further kinetic heterogeneity with two distinct sub-groups: rapidly replaced and long-lived SCMs (8, 76, 77). CD95 marker expression has been used to distinguish SCM cells from their naïve counterparts (8, 52). In the proposed model, the $T_{N4}$ variable representing the naïve CD4^+^ T-cell subset was calibrated based on CD45RA^+^/CD45RO^–^ CCR7^+^/CD62L^+^ CD4^+^ T-cell quantitative data (18). Therefore, the model variable describing the naïve T-lymphocyte subpopulation contains a subpopulation of SCM cells, along with true naïve cells. Based on this consideration, model parameters for the naïve subset can be interpreted in a different manner. The homeostatic CD4^+^ T-cell kinetic model suggested capturing the “steady-state” activation of the immune system after the first encounter with an antigen with the naïve CD4^+^ T-cell differentiation rate parameter, $\varphi_{N4}$. Considering the embedding of naïve cells with SCM cells within this one model variable, the parameter $\varphi_{N4}$ may also be reflective of a steady-state transition from naïve to SCM cells, without antigen involvement. The naïve CD4^+^ T-cell proliferation rate parameter, $\lambda_{N4}$, may also include SCM cell homeostatic properties, such as self-renewal. Owing to the detected age-related changes of the parameter $\lambda_{N4}$, which were associated with RTE cell concentration dependencies ( $\lambda_{N4}\left(age\right)$ and $\lambda_{N4}\left(T_{RTE4_{cells\_uL}}^{BL}\right)$, the age-associated alterations in SCM cells homeostasis may be presumed to influence $\lambda_{N4}$, in addition to the naïve T-cell adaptation to a decreased thymic output. Moreover, in spite of kinetic heterogeneity in SCM T-cells (76, 77), the activated cell model variable, $T_{A4}$, could capture the SCM subset with enhanced self-renewal properties. In view of the sensitivity analysis results, whereby activated CD4^+^ T-cell homeostasis parameters had the greatest influence on memory subset homeostasis, the impact of SCM cell proliferative activity on long-lived immune memory maintenance was implicitly considered, along with the clonal expansion effect. However, the magnitude of this impact, as well as an overall characterization of SCM T-lymphocyte homeostasis, would require an additional analysis and model expansion by considering “true” naïve CD95^–^ and SCM CD95^+^ subpopulations, along with an inventory of all relevant quantitative data sources pertaining to these phenotypic subsets.

Another limitation of the present analysis is that resident-memory (RM) CD4^+^ T-cells, which play a key role in local immunity and immune memory maintenance (78), were not directly represented in the model structure. Depending on the marker expressions being used for tissue residency, RM CD4^+^ cells may constitute 63.9 – 92.2% (CD45RO^+^CD69^+^) and 54.1 – 75.2% (CD45RO^+^CD103^+^) in the gastro-intestinal tract, and 5.9 – 29.3% (CD45RO^+^CD69^+^) and 1.9 – 4.5% (CD45RO^+^CD103^+^) in the lungs, relative to the total amount of memory CD4^+^ T-cells [calculated in (18), based on data from (79)]. Indirectly, the residency of cells in organs was captured by CM and EM cells, which could fail to recirculate, in the respective organs. The incorporation of RM CD4^+^ T-cells as a distinct variable would represent one of the next steps in expanding the present model, conditional on the availability of age-dependent data for RM cell percentages in various organs (79).

The influence of non-immunological factors, such as sex and environmental exposures, which can plausibly modify age-dependent CD4^+^ T-cell homeostasis, requires further investigation. Large human studies have reported sex-associated differences in immune aging, including shifts in adaptive immune compartments and naïve T-cell features, suggesting that sex may influence the inferred age trends (80). Mechanistically, sex steroid hormones are recognized contributors to thymic remodeling via effects on thymic epithelial compartments, providing a biological basis for potential sex-specific thymic trajectories (74, 75). In the previously performed systematic review and meta-analysis of age-dependent T-lymphocyte homeostasis, evaluation of sex effects was attempted; however, sex was inconsistently reported across primary sources, thereby limiting statistical power (18). In a preliminary analysis combining individual observations with known sex and single-sex cohort studies, no significant male–female differences were detected in the age dynamics of total lymphocytes and major T-cell counts, while the CD4^+^/CD8^+^ T-cell ratio tended to be higher in females without reaching significance (18). The developed model utilized an age-dependent blood volume function scaled from a published body weight vs. age relationship (54). Although the underlying model included sex as a covariate, sex-specific scaling was not considered in model calibration because (i) sex was not consistently reported across the data sources used to compile the calibration dataset, and (ii) the aggregated meta-analytic estimates of T-cell counts did not show robust sex differences in the available homeostatic database. At the same time, the developed model can be readily extended by introducing a sex-specific correction factor at the blood-volume parameterization step, enabling sex-specific predictions of T-cell concentrations without altering the cellular kinetic equations. Quantifying sex effects at the level of homeostatic kinetic parameters would, however, require an additional sex-stratified dataset with sufficient longitudinal or age-resolved immunophenotyping. Environmental factors such as the gut microbiome may also shape immune aging through age-associated dysbiosis, altered barrier integrity, and inflammaging (81); however, assessing these effects would require dedicated datasets.

Several assumptions were made during model development regarding T-cell death rates and transition rates between organs. Death rates for the various CD4^+^ T-lymphocyte subpopulations were fixed based on experimental estimates and were assumed to be equal in each physiological compartment, in order to capture immune homeostasis. However, in an active immune response, death rates may vary across different organs, especially for the effector subset with an arguably higher death rate at the site of infection. Another assumption relates to the kinetic transition rates of specific subpopulations in the gastro-intestinal tract and lungs compartments, which were driven by comparable cell percentages for each CD4^+^ subpopulation in these compartments. Despite differences in volumetric flow rate of blood between lungs and the gastro-intestinal tract, kinetics of blood cells are comparable, e.g., as approximated from blood cell flow (ml/h) and volume-based (ml) physiological parameters from the Shah and Betts PBPK model (82). In the present model, the identified age-related changes in T-cell migration – namely, a reduced transition from lymphoid tissue to blood and an increased effector CD4^+^ transition to peripheral organs – reflect mechanisms reported in experimental studies (83, 84). Specifically, aging process contributed not only to a decline in lymph node numbers and lymphatic system deterioration, but also to a decrease in high endothelial venule (HEV) numbers, which further contributed to age-dependent cell migration alterations (83). The observed increase in effector cell transfer to peripheral sites with age may further correspond to inflammaging processes and increased intestinal mucosal permeability (84).

## 5 Conclusions

A multiscale mechanistic model of homeostatic CD4^+^ T-lymphocyte cellular kinetics was developed, integrating maturation, peripheral differentiation and migration aspects, as well as age-related changes in the homeostasis of various cell subpopulations, from thymocytes to effector cells. A sequential approach to model development was proposed, to subsequently characterize cellular kinetics in newborns, capture age-dependent homeostasis of CD4^+^ T-lymphocytes, and evaluate reciprocal cellular influences on homeostasis. An analysis of generalized data, from multiple sources, on cell concentrations in various organs and across narrow age groups, combined with experimental estimates of cellular kinetics enabled a consistent representation of quantitative, age-dependent homeostasis of CD4^+^ T-cells and the conduction of *in silico* experiments to evaluate the immune system’s behavior following homeostatic disturbances. Age-dependent changes in several processes were identified as significant features of the model for describing CD4^+^ T-lymphocyte homeostasis in healthy humans. These processes include naïve and activated cell proliferation, RTE cell death, central-memory and effector-memory cell differentiation. In addition, age-dependent cellular transitions from lymphoid tissue to blood and peripheral organs as well as decreased thymocyte egress as a result of thymic involution were identified as significant features to maintain CD4^+^ T-lymphocyte homeostasis. A model-based sensitivity analysis revealed that thymocyte homeostasis parameters, as well as the differentiation, proliferation and transition rates of naïve CD4^+^ T-lymphocytes were the more important processes influencing concentrations of lesser differentiated subpopulations, while for more differentiated memory and effector cells, the extent of clonal expansion appeared to be the most critical process. The degree of influence of the abovementioned processes was found to decrease with age. The compensatory mechanism of an increase in the naïve T-cell proliferation rate and a decrease in the RTE cell death rate, in response to a declining number of RTE cells, was essential to capture perturbations in cellular homeostasis. As a result of evaluating the restoration of CD4^+^ T-lymphocyte counts upon thymectomy, an increase in naïve T-cell proliferation was found to be insufficient to maintain cell counts, decades after the procedure. The present mechanistic model of immunological homeostasis enabled a systematic aggregation of all heterogeneous experimental and clinical information on cell homeostasis and their integration into biological mechanisms, to obtain an accurate, time-dependent description of cell counts in various tissue compartments and to perform quantitative simulations of various clinically relevant scenarios.

The developed homeostatic model of CD4^+^ T-lymphocyte cellular kinetics provides a quantitative description of maturation, activation and differentiation of distinct CD4^+^ T-cell subpopulations, together with age-related changes in homeostasis. Besides the identification of key alterations in homeostatic processes during aging, the proposed model also provides an instrumental tool for personalized age-conditioned CD4^+^ T-lymphocyte immune profiling, which can be further individualized using patient-specific immunophenotyping data, and for generating immune response predictions across the lifespan of healthy individuals. Incorporation of cell transition aspects in the model further provides an opportunity to evaluate local infection scenarios and/or organ-specific immune response dynamics, for example an HIV infection scenario which is known to strongly affect the gastro-intestinal tract. Moreover, the developed model represents a quantitative framework to investigate alterations in cell homeostasis under HIV or other infections, for which questions concerning regulations of the immune system remain unaddressed. For future perspective, the developed model can be used as a core module in a quantitative systems pharmacology platform, for practical use in drug or vaccine development, in accordance with model-informed drug discovery and development (MIDD) principles (57).

## Data Availability

The original contributions presented in the study are included in the article/**Supplementary Material**. Further inquiries can be directed to the corresponding author.
