# Supplementary material for: Multiscale physiologically-based model of age-dependent CD4+ T-lymphocyte homeostasis
Source: Front Immunol. 2026 Feb 4;17:1742817. doi: 10.3389/fimmu.2026.1742817 (PMC12914733; doi:10.3389/fimmu.2026.1742817)
Supplement: Supplementary file 1 [file Table1.docx]

Supplementary Material

Multiscale physiologically-based model of age-dependent CD4+ T-lymphocyte homeostasis

Victoria Kulesh^1,2,3*^, Kirill Peskov^1,2,4^, Gabriel Helmlinger^5^, Gennady Bocharov^2,6,7^

*** Correspondence:** Victoria Kulesh: [viktoriaan37@gmail.com](mailto:viktoriaan37@gmail.com)

Table of contents

[1 Supplementary Tables 3](#_Toc219670626)

[1.1 Supplementary Table 1 3](#_Toc219670627)

[1.2 Supplementary Table 2 6](#_Toc219670628)

[1.3 Supplementary Table 3 18](#_Toc219670629)

[1.4 Supplementary Table 4 31](#_Toc219670630)

[1.5 Supplementary Table 5 47](#_Toc219670631)

[1.6 Supplementary Table 6 48](#_Toc219670632)

[1.7 Supplementary Table 7 52](#_Toc219670633)

[1.8 Supplementary Table 8 55](#_Toc219670634)

[1.9 Supplementary Table 9 57](#_Toc219670635)

[1.10 Supplementary Table 10 58](#_Toc219670636)

[1.11 Supplementary Table 11 61](#_Toc219670637)

[2 Supplementary Figures 65](#_Toc219670638)

[2.1 Supplementary Figure 1 66](#_Toc219670639)

[2.2 Supplementary Figure 2 67](#_Toc219670640)

[2.3 Supplementary Figure 3 68](#_Toc219670641)

[2.4 Supplementary Figure 4 69](#_Toc219670642)

[2.5 Supplementary Figure 5 71](#_Toc219670643)

[2.6 Supplementary Figure 6 72](#_Toc219670644)

[2.7 Supplementary Figure 7 73](#_Toc219670645)

[2.8 Supplementary Figure 8 74](#_Toc219670646)

[2.9 Supplementary Figure 9 76](#_Toc219670647)

[2.10 Supplementary Figure 10 78](#_Toc219670648)

[2.11 Supplementary Figure 11 80](#_Toc219670649)

[2.12 Supplementary Figure 12 81](#_Toc219670650)

[2.13 Supplementary Figure 13 82](#_Toc219670651)

[2.14 Supplementary Figure 14 83](#_Toc219670652)

[2.15 Supplementary Figure 15 84](#_Toc219670653)

[2.16 Supplementary Figure 16 85](#_Toc219670654)

[2.17 Supplementary Figure 17 85](#_Toc219670655)

[2.18 Supplementary Figure 18 87](#_Toc219670656)

[2.19 Supplementary Figure 19 88](#_Toc219670657)

[2.20 Supplementary Figure 20 89](#_Toc219670658)

[2.21 Supplementary Figure 21 89](#_Toc219670659)

[2.22 Supplementary Figure 22 90](#_Toc219670660)

[3 Model code 91](#_Toc219670661)

[4 References 103](#_Toc219670662)

# Supplementary Tables

## Supplementary Table 1

**Supplementary Table 1**. Clinical data used for model development

| **Data description** | **Organ (units)** | **Age range**  **(years)** | **Data points number** | **Data type** | **Data assignment** | **Reference** |
| --- | --- | --- | --- | --- | --- | --- |
| *CD4+ T-lymphocyte subpopulations data from healthy subjects* | | | | | | |
| Recent thymic emigrants  *(**CD45RA+*  *CD31+)* | Blood (cells/μL) | 0.20 – 64.75 | 10 | Weighted averages | Calibration | (1) |
|  | Lymphatic tissue (%) | 1.60 – 52.39 | 4 | Weighted averages | Calibration | (1) |
| Naïve CD4+ T-lymphocytes  *(**CD45RA+*  *CD45RO-*  *CCR7+*  *CD62L+)* | Blood (cells/μL) | 0.08 – 85.03 | 13 | Weighted averages | Calibration | (1) |
|  | Lymphatic tissue (%) | 1.56 – 54.70 | 4 | Weighted averages | Calibration | (1) |
|  | GI tract (%) | 1.56 – 54.83 | 4 | Weighted averages | Calibration | (1) |
|  | Lungs (%) | 1.56 – 54.50 | 3 | Weighted averages | Calibration | (1) |
| Activated CD4+ T-lymphocytes  *(**CD38+*  *HLADR+)* | Blood (cells/μL) | 0.25 – 65.00 | 7 | Weighted averages | Calibration | (1) |
|  | Lymphatic tissue (%) | 39.2 | 1 | Weighted averages | Calibration | (1) |
| Central-memory CD4+ T-lymphocytes  *(**CD45RA-*  *CD45RO+*  *CCR7+*  *CD62L+)* | Blood (cells/μL) | 0.06 – 68.32 | 11 | Weighted averages | Calibration | (1) |
|  | Lymphatic tissue (%) | 1.56 – 54.70 | 4 | Weighted averages | Calibration | (1) |
|  | GI tract (%) | 1.56 - 54.83 | 4 | Weighted averages | Calibration | (1) |
|  | Lungs (%) | 1.56 – 54.50 | 3 | Weighted averages | Calibration | (1) |
| Effector-memory CD4+ T-lymphocytes  *(CD45RA-*  *CD45RO+*  *CCR7-*  *CD62L-)* | Blood (cells/μL) | 0.06 – 66.32 | 10 | Weighted averages | Calibration | (1) |
|  | Lymphatic tissue (%) | 1.56 – 54.70 | 4 | Weighted averages | Calibration | (1) |
|  | GI tract (%) | 1.56 – 54.83 | 4 | Weighted averages | Calibration | (1) |
|  | Lungs (%) | 1.56 – 54.50 | 3 | Weighted averages | Calibration | (1) |
| Effector CD4+ T-lymphocytes  *(CD45RA+*  *CD45RO-*  *CCR7-*  *CD62L-)* | Blood (cells/μL) | 0.19 – 68.32 | 9 | Weighted averages | Calibration | (1) |
|  | Lymphatic tissue (%) | 1.56 – 55.62 | 4 | Weighted averages | Calibration | (1) |
|  | GI tract (%) | 1.56 – 54.83 | 4 | Weighted averages | Calibration | (1) |
|  | Lungs (%) | 1.56 – 54.50 | 3 | Weighted averages | Calibration | (1) |
| Total CD4+ T-lymphocytes | Blood (cells/μL) | 0.04 – 104.74 | 16 | Weighted averages | Validation | (1) |
| Total memory CD4+ T-lymphocytes  *(CD45RO+)* | Blood (cells/μL) | 0.25 – 78.27 | 12 | Weighted averages | Validation | (1) |
|  | Lymphatic tissue (%) | 16.36 – 56.25 | 3 | Weighted averages | Validation | (1) |
|  | GI tract (%) | 17.00 – 53.20 | 3 | Weighted averages | Validation | (1) |
|  | Lungs (%) | 17.00 – 53.2 | 3 | Weighted averages | Validation | (1) |
| *Physiological and anatomical parameters related to the thymus and circulatory system* | | | | | | |
| Total blood volume data | Blood (L) | 0.02 – 86.10 | 145 | Individual | Calibration | (2,3) |
|  | Blood (L) | 0.01 – 0.99 | 14 | Aggregated | Calibration | (4) |
|  | Blood (L) | 0 – 70 | 13 | Aggregated | Validation | (5–7) |
| Thymus wet weight | Thymus (g) | 0.76 – 98.9 | 574 | Individual | Calibration  *(previously used for thymus wet weight age dependency calibration in thymocyte homeostasis model* (8)*)* | (9) |
|  | Thymus (g) | 0 – 26 | 191 | Individual | Calibration  *(new data source for thymus wet weight age dependency calibration in thymocyte homeostasis model (10))* | (10) |
| *CD4+ T-lymphocyte subpopulations data from thymectomized subjects* | | | | | | |
| Total CD4+ T-lymphocytes | Blood (cells/μL) | 0 – 4.50  (thymectomy age range: 0 – 0.3) | 16 | Individual | Validation | (11) |
| Naive CD4+ T-lymphocytes  *(CD45RO- CCR7+)* | Blood (cells/μL) | 20 – 27  (thymectomy age range: 1 – 12) | 10 | Individual | Validation | (12) |

## Supplementary Table 2

**Supplementary Table 2**. Kinetic characteristics of CD4+ T-lymphocyte subpopulations

| **Parameter name** | **Definition** | **Experimental values** | **Experimental estimates range** |
| --- | --- | --- | --- |
| $\mu_{rte4}$ | Death rate of CD4+ RTE T-cells | **0.00092 – 0.0015** d^-1^ [*vanHoeven2017, mouse model, deuterium labeled data*] (13) (scaled for human)  (for derivation see Supplementary Table 3 (*derivation #1*))  **0.0076** d^-1^ [*Haines2009, thymectomized human data*] (14) – approximated death and differentiation rates to naïve cells  (for derivation see Supplementary Table 3 (*derivation #2*)) | [**0.00092; 0.0076**] d^-1^ |
| $\omega_{rte4_{bl\_lt}}$ | Transition rate of CD4+ RTE T-cells from blood to lymphatic tissue | 0**.459 – 0.917** d^-1^ [*vanHoeven2017, mouse model, deuterium labeled data*] (13) (scaled for human)  (for derivation see Supplementary Table 3 (*derivation #3*)) | [**0.459; 0.917**] d^-1^ |
| $\varphi_{rte4}$ | Differentiation rate of CD4+ RTE T-cells to CD4+ naïve T-cells | **0.0005** d^-1^ [*Vrisekoop2008, human model, deuterium labeled data*] (15) (turnover rate for naïve cells)  **0.00014 – 0.0012** d^-1^ [*vanHoeven2017, mouse model, deuterium labeled data*] (13) (scaled for human)  (for derivation see Supplementary Table 3 (*derivation #1*)) | [**0.00014; 0.0012**] d^-1^ |
| $\mu_{N4}$ | Death rate of CD4+ naive T-cells | **0.002 – 0.006** d^-1^ ($t_{1/2}$ = 120-365 days [*Richman2000*] (16))  **0.000457** d^-1^ ($t_{1/2}$ = 1517 days [*Vrisekoop2008, human model, deuterium labeled data*] (15) (from turnover rate))  **0.0011** d^-1^ ($t_{1/2}$ = 630 days [*Michie1992, human data after radiation*] (17) (include CD45RA+CD27- effector cells))  **0.0059** d^-1^ ($t_{1/2}$ = 118 days [*Macallan2003, human Asquith et al. - based model* (18) *accounting cellular heterogeneity, deuterium labeled data*] (19))  **0.0019** d^-1^ ($t_{1/2}$ = 361 days [*Macallan2004, human Asquith et al. - based model* (18) *accounting cellular heterogeneity, deuterium labeled data*] (20))  **0.0038 – 0.0058** d^-1^ ($t_{1/2}$ = 119 (young) – 184 (old) days [*Wallace2004, human Asquith et al. - based model* (18) *accounting cellular heterogeneity, deuterium labeled data*] (21))  (for derivation of the estimated parameters above see Supplementary Table 3 (*derivation #4*). The assumption that $t_{1/2}$ of cells corresponds only to death rate was made)  **0.0725** d^-1^ [*Macallan2003, human Asquith et al. - based model* (18) *accounting cellular heterogeneity, deuterium labeled data*] (19) (estimated disappearance rate for CD4+ CD45RA+ cells)  **0.0104** d^-1^ [*Mueller-Schoell2021, human CAR-T QSP model*] (22) (CD4+ and CD8+, CAR-T)  **0.03665** d^-1^ [*Schubert2000, in vitro data*] (23) (total T-cells in blood)  (for derivation see Supplementary Table 3 (*derivation #5*)) | [**0.000457; 0.0725**] d^-1^ |
| $\lambda_{N4}$ | Proliferation rate of CD4+ naïve T-cells | **0.00078** d^-1^ [95CI: 0.00046;0.0011] [*McLean_Michie1995, human model, data from patients, rebuilding their lymphocyte pool after radiotherapy*] (24) (estimated proliferation rate for unprimed CD4+ CD45RA+ cells)  **0.006** d^-1^ [*Macallan2003, human Asquith et al. - based model* (18) *accounting cellular heterogeneity, deuterium labeled data*] (19) (estimated proliferation rate for CD4+ CD45RA+ cells)  **0.0005** d^-1^ [*Vrisekoop2008, human model, deuterium labeled data*] (15) (turnover rate for naïve cells)  **0.0007** d^-1^ (0.0004-0.001) [*Macallan2019, human model, deuterium labeled data*] (25) (best estimate from unpublished Costa del Amo et al. results, CD45RO- CD27(bright) CCR7+ CD95-) | [**0.0005; 0.006**] d^-1^ |
| $\varphi_{N4}$ | Differentiation rate of naïve to activated CD4+ T-cells | **0.5 – 0.67** d^-1^ [*Kaech2002*] (26) (clonal expansion of CD4+ T-cells is observed 36–48 hours after the initial stimulus)  (for derivation see Supplementary Table 3 (*derivation #6*))  **0.000005 - 0.000067** d^-1^ (derived by differentiation rate after stimuli (26) and fraction of T-cells, responding to stimuli (27))  (for derivation see Supplementary Table 3 (*derivation #7*))  **0.093** d^-1^ [*Mueller-Schoell2021, human CAR-T QSP model*] (22) (CD4+ and CD8+, CAR-T, estimated differentiation rate of naïve to central memory cells = 0.14 d^-1^, calculated based on CD4/CD8 ratio)  (for derivation see Supplementary Table 3 (*derivation #8*))  **0.0002** d^-1^ [*Bajaria2002, human model*] (28) (estimated parameter for differentiation rate from naïve to memory cells) | [**0.000005; 0.093**] d^-1^ |
| $\omega_{N4_{lt-bl}}$ | Transition rate of CD4+ naïve T-cells from lymphatic tissue to blood | **0.13** d^-1^ [*Sprent1973*, *mice data*] (29,30) (scaled for human)  **0.276** d^-1^ [*Mandl2012, mice data, exponential model, experiments with blocked entry in LN, exponential model*] (31) (Peripheral LNs) (scaled for human)  **0.368** d^-1^ [*Mandl2012, mice data, exponential model, experiments with blocked entry in LN, exponential mode*l] (Mesenteric LNs) (31) (scaled for human)  (for derivation of the estimated parameters above see Supplementary Table 3 (*derivation #9*))  **0.26** d^-1^ (human scaling from (27,31)  (for derivation see Supplementary Table 3 (*derivation #10*)) | [**0.13; 0.368**] d^-1^ |
| $\omega_{N4_{bl-lt}}$ | Transition rate of CD4+ naïve T-cells from blood to lymphatic tissue | **40.0** d^-1^ [*Sprent1973*, *mice data*] (29,30) (assumption that transition rate from blood to lymphatic tissue is the same for humans) | **40.0** d^-1^ |
| $\omega_{N4_{bl-git}}$, d^-1^  $\omega_{N4_{bl-lung}}$, d^-1^  $\omega_{N4_{bl-tis}}$, d^-1^  $\omega_{A4_{bl-tis}}$, d^-1^ | Transition rate of naïve CD4+ T-cells from blood to gastro-intestinal tract / lungs / other peripheral tissues and activated CD4+ T-cells from blood to peripheral tissues | *Values are obtained based on the sensitivity analysis and likelihood profiling analysis of the developed model (see section 3.4.3,* ***Supplementary Figure 22A****)* | **[0.0002; 0.01]** d^-1^ |
| $\lambda_{A4}$ | Proliferation rate of activated CD4+ T-cells | **1.725** d^-1^ [*Kaech2002, Malhotra2020*] (26,32) (division time of CD4+ T-cells during clonal expansion = ~ 10 h per cell division)  (for derivation see Supplementary Table 3 (*derivation #11*)) | **1.725** d^-1^ |
| $\varphi_{A4}$ | Differentiation rate of activated CD4+ T-cells | **0.093** d^-1^ [*Mueller-Schoell2021, human CAR-T QSP model*] (22) (CD4+ and CD8+, CAR-T, estimated differentiation rate of naïve to central memory cells = 0.14 d^-1^, calculated based on CD4/CD8 ratio)  (for derivation see Supplementary Table 3 (derivation #8))  **0.000026 – 0.0005** d^-1^ [*Gossel2017, mice data*] (33) (CD4+ N-to-CM, scaled for human)  **0.000034 – 0.00033** d^-1^ [*Gossel2017, mice data*] (33) (CD4+ N-to-EM(EFF), scaled for human)  (for derivation see Supplementary Table 3 (*derivation #12*))  **0.00018 – 0.0021** d^-1^ [*Gossel2017, mice data*] (33) (CD4+ N-to-CM, scaled for human)  **0.00024 – 0.0014** d^-1^ [*Gossel2017, mice data*] (33) (CD4+ N-to- EM(EFF), scaled for human)  (for derivation see Supplementary Table 3 (*derivation #13*))  Overall diff. rate = **0.00042 – 0.0035** d^-1^ | [**0.00042; 0.093**] d^-1^ |
| $\omega_{A4_{lt-bl}}$ | Transition rate of CD4+ activated T-cells from lymphatic tissue to blood | (Assumed to be the same as for naïve $\omega_{{N4}_{lt-bl}}$) | [**0.13; 0.368**] d^-1^ |
| $\omega_{A4_{bl-lt}}$ | Transition rate of CD4+ activated T-cells from blood to lymphatic tissue | (Assumed to be the same as for naïve  $\omega_{{N4}_{bl-lt}}$) | **40.0** d^-1^ |
| $\mu_{A4}$ | Death rate of activated CD4 T-cells | **0.04** d^-1^ [*Ribeiro2002, human deuterated glucose labeling data, model value*] (34)  **0.028** d^-1^ [*Biancotto2008*] (35) (calculated from ex vivo experiments for uninfected activated cells, CD69+ cells)  (for derivation see Supplementary Table 3 (*derivation #14*)) | [**0.028;0.04**] d^-1^ |
| $f_{4}$ | Fraction of activated CD4+ T-cells, differentiated to central-memory CD4+ T-cells | **0.43 – 0.6** [*Gossel2017, mice data*] (33) (calculated based on proliferation rates)  (for derivation see Supplementary Table 3 (*derivation #15*)) | [**0.43; 0.6**] d^-1^ |
| $\lambda_{CM4}$ | Proliferation rate of central-memory CD4+ T-cells | **0.0065** d^-1^ [95CI: 0.00511;0.00789] [*McLean-Michie1995, human model, data from patients, rebuilding their lymphocyte pool after radiotherapy*] (24) (estimated proliferation rate for unprimed CD4+ CD45RO+ cells)  **0.027** d^-1^ [*Macallan2003, human Asquith et al. - based model* (18) *accounting cellular heterogeneity, deuterium labeled data*] (19) (estimated proliferation rate for CD4+ CD45RO+ cells)  **0.010** d^-1^ [*Macallan2004, human Asquith et al. - based model* (18) *accounting cellular heterogeneity, deuterium labeled data*] (20) (CD45RO+CCR7+ cells)  **0.0391** d^-1^ (0.0234, 0.0716) [*Gossel2017, mice data*] (33) (scaled to human)  (for derivation see Supplementary Table 3 (*derivation #16*)) | [**0.0065; 0.0391**] d^-1^ |
| $\mu_{CM4}$ | Death rate of CM CD4 cells | **0.006 – 0.02** d^-1^ ($t_{1/2}$ = 1-4 months [*Richman2000, Bajaria2002*] (16,28))  (for derivation of the estimated parameters above see Supplementary Table 3 (*derivation #4*). The assumption that $t_{1/2}$ of cells corresponds only to death rate was made)  **0.0737** d^-1^ [*Macallan2003, human Asquith et al. - based model* (18) *accounting cellular heterogeneity, deuterium labeled data*] (19) (estimated disappearance rate for CD4+ CD45RO+ cells)  **0.041** d^-1^ [*Macallan2004, human Asquith et al. - based model* (18) *accounting cellular heterogeneity, deuterium labeled data*] (20) (estimated disappearance rate for CD45RO+CCR7+ cells)  **0.0104** d^-1^ [*Mueller-Schoell2021, human CAR-T QSP model*] (22) (CD4+ and CD8+, CAR-T) | [**0.006; 0.041**] d^-1^ |
| $\omega_{CM4_{lt-bl}}$ | Transition rate of CD4+ central-memory T-cells from lymphatic tissue to blood | **0.035** d^-1^ [*Sprent1973*, *mice data*] (29,30) (scaled for human) (for total memory subset)  (for derivation of the estimated parameters above see Supplementary Table 3 (*derivation #9*)) | **0.035** d^-1^ |
| $\omega_{CM4_{bl-lt}}$ | Transition rate of CD4+ central-memory T-cells from blood to lymphatic tissue | **10.0** d^-1^ [*Sprent1973*, *mice data*] (29,30) (assumption that transition rate from blood to lymphatic tissue is the same for humans) | **10.0** d^-1^ |
| $\varphi_{CM4}$ | Differentiation rate of central-memory to effector-memory CD4+ T-cells | **0.00082 – 0.0067** d^-1^ [*Gossel2017, mice data*] (33) (CD4+ CM-to-EM, scaled for human)  (for derivation of the estimated parameters above see Supplementary Table 3 (*derivation #12*))  **0.00057 – 0.05** d^-1^ [*Gossel2017, mice data*] (33) (CD4+ CM-to-EM, scaled for human)  (for derivation of the estimated parameters above see Supplementary Table 3 (*derivation #13*))  **0.111** d^-1^ [*Mueller-Schoell2021, human CAR-T QSP model*] (22) (CD4+ and CD8+, CAR-T, estimated differentiation rate of central-memory to effector-memory cells = 0.191 d^-1^, calculated based on CD4/CD8 ratio)  (for derivation see Supplementary Table 3 (*derivation #8*)) | [**0.00082; 0.111**] d^-1^ |
| $\omega_{CM4_{bl-git}}$, d^-1^  $\omega_{CM4_{bl-lung}}$, d^-1^  $\omega_{CM4_{bl-tis}}$, d^-1^ | Transition rate of central-memory CD4+ T-cells from blood to gastro-intestinal tract / lungs / other peripheral tissues | *Values are obtained based on the sensitivity analysis and likelihood profiling analysis of the developed model (see section 3.4.3,* ***Supplementary Figure 22B****)* | **[0.03; 5]** d^-1^ |
| $\lambda_{EM4}$ | Proliferation rate of effector-memory CD4+ T-cells | **0.0065** d^-1^ [95CI: 0.00511;0.00789] [*McLean_Michie1995, human model, data from patients, rebuilding their lymphocyte pool after radiotherapy*] (24) (estimated proliferation rate for unprimed CD4+ CD45RO+ cells)  **0.027** d^-1^ [*Macallan2003, human Asquith et al. - based model* (18) *accounting cellular heterogeneity, deuterium labeled data*] (19) (estimated proliferation rate for CD4+ CD45RO+ cells)  **0.042** d^-1^ (0.02-0.08) [*Macallan2004, human Asquith et al. - based model* (18) *accounting cellular heterogeneity, deuterium labeled data*] (20) (CD45RO+CCR7- cells)  **0.03822** d^-1^ (0.0213; 0.04) [*Gossel2017, mice data*] (33) (scaled for human)  (for derivation see Supplementary Table 3 (*derivation #16*)) | [**0.0065; 0.042**] d^-1^ |
| $\mu_{EM4}$ | Death rate of effector-memory CD4+ T-cells | **0.006 – 0.02** d^-1^ ($t_{1/2}$ = 1-4 months [*Richman2000, Bajaria2002*] (16,28))  (for derivation of the estimated parameters above see Supplementary Table 3 (*derivation #4*). The assumption that $t_{1/2}$ of cells corresponds only to death rate was made)  **0.0737** d^-1^ [*Macallan2003, human Asquith et al. - based model* (18) *accounting cellular heterogeneity, deuterium labeled data*] (19) (estimated disappearance rate for CD4+ CD45RO+ cells)  **0.11** d^-1^ [*Macallan2004, human Asquith et al. - based model* (18) *accounting cellular heterogeneity, deuterium labeled data*] (20) (estimated disappearance rate for CD45RO+CCR7- cells)  **0.0104** d^-1^ [*Mueller-Schoell2021, human CAR-T QSP model*] (22) (CD4+ and CD8+, CAR-T) | [**0.006; 0.11**] d^-1^ |
| $\varphi_{EM4}$ | Differentiation rate of EM CD4 cells to Eff CD4 cells | **0.205** d^-1^ [*Mueller-Schoell2021, human CAR-T QSP model*] (22) (CD4+ and CD8+, CAR-T, estimated differentiation rate of effector-memory to effector cells = 0.355 d^-1^, calculated based on CD4/CD8 ratio)  (for derivation see Supplementary Table 3 (*derivation #8*)) | **0.205** d^-1^ |
| $\omega_{EM4_{lt-bl}}$ | Transition rate of CD4+ effector-memory T-cells from lymphatic tissue to blood | **0.035** d^-1^ [*Sprent1973*, *mice data*] (29,30) (scaled for human) (for total memory subset)  (for derivation of the estimated parameters above see Supplementary Table 3 (*derivation #9*))  **0.156** d^-1^ [*Ganusov and Tomura 2021, Sprent1976, mica data*] (27,36) (scaled for human, estimates for CD4+ and CD8+ effector-memory and effector cells)  (for derivation of the estimated parameters above see Supplementary Table 3 (*derivation #17*)) | [**0.035; 0.156**] d^-1^ |
| $\omega_{EM4_{bl-git}}$ | Transition rate of CD4+ effector-memory T-cells from blood to gastro-intestinal tract and other peripheral tissues | **5.85** d^-1^ [*Ganusov and Tomura 2021, Sprent1976, mica data*] (27,36) (scaled for human, estimates for CD4+ and CD8+ effector-memory and effector cells)  (for derivation of the estimated parameters above see Supplementary Table 3 (*derivation #17*)) | **5.85** d^-1^ |
| $\mu_{Eff4}$ | Death rate of effector CD4+ T-cells | **0.518** d^-1^ [*Mueller-Schoell2021, human CAR-T QSP model*] (22) (CD4+ and CD8+, CAR-T)  **0.6** d^-1^ [*Macallan2004, human Asquith et al. - based model* (18) *accounting cellular heterogeneity, deuterium labeled data*] (20) (estimated disappearance rate for CD45RA+CCR7- cells) | [**0.518 - 0.6**] d^-1^ |
| $\omega_{Eff4_{lt-bl}}$ | Transition rate of CD4+ effector T-cells from lymphatic tissue to blood | **0.156** d^-1^ [*Ganusov and Tomura 2021, Sprent1976, mica data*] (27,36) (scaled for human, estimates for CD4+ and CD8+ effector-memory and effector cells)  (for derivation of the estimated parameters above see Supplementary Table 3 (*derivation #17*)) | **0.156** d^-1^ |
| $\omega_{Eff4_{bl-git}}$ | Transition rate of CD4+ effector T-cells from blood to gastro-intestinal tract and other peripheral tissues | **5.85** d^-1^ [*Ganusov and Tomura2021, Sprent1976, mica data*] (27,36) (scaled for human, estimates for CD4+ and CD8+ effector-memory and effector cells)  (for derivation of the estimated parameters above see Supplementary Table 3 (*derivation #17*)) | **5.85** d^-1^ |
| $T_{{A4}_{max}}$ | Maximal carrying capacity of activated CD4+ T-cells proliferation | **10^13^** cells [*Kulesh2025, Sender2023, Malhotra2020*] (1,32,37) (calculated based on the fold expansion of activated HLADR+ CD38+ cells during acute infection)  (for derivation of the estimated parameters above see Supplementary Table 3 (*derivation #18*)) | **10^13^** cells |
| $\omega_{EM4_{git-bl}}$ | Transition rate of CD4+ effector T-cells from gastro-intestinal tract / lungs/ other peripheral tissues to blood | **0.005 – 0.01** d^-1^ [*Chandler2025*] (38) (scaled for human)  **0.3** d^-1^ [*Ganusov and Tomura 2021*] (27) (scaled for human, estimates for CD4+ and CD8+ effector-memory and effector cells)  (for derivation of the estimated parameters above see Supplementary Table 3 (*derivation #17*)) | [**0.005 – 0.3**] d^-1^ |

## Supplementary Table 3

**Supplementary Table 3.** Derivation of experimental estimates

| **Derivation #** | **Process name** | **Detailed description of derivation** |
| --- | --- | --- |
| 1 | Death and differentiation rates of CD4+ RTE T-cells | Human life is 30-50 times longer than mouse life (39) ==> Lifespan (human) = 30-50 * lifespan (mouse)  According to *Perelson et al.* (40):  $Residence time= \frac{1}{export time}$  Assume the same equation for lifespan:  $Lifespan= \frac{1}{diff. rate+death rate}$  Hence:  $Death rate \left( human \right)=\left( \frac{1}{30}or\frac{1}{50} \right)*death rate (mouse)$  $Diff. rate \left( human \right)=\left( \frac{1}{30}or\frac{1}{50} \right)*diff. rate (mouse)$  Estimates from *vanHoeven2017* (13) (mouse model)  RTE CD4+ death rate = 0.046 d^-1^ (95% CI: [0.028; 0.081]) ==> **Scaled values for human: [0.00092; 0.0015] d^-1^**  Derivation:  0.046 * 1/30 = 0.0015 d^-1^ ; 0.046 * 1/50 = 0.00092 d^-1^  RTE CD4+ diff rate = 0.017 d^-1^ (95% CI: [0.007; 0.035]) ==> **Scaled values for human:** [0.00014; 0.0012] d^-1^  Derivation:  0.007 * 1/30 = 0.00023 d^-1^; 0.007 * 1/50 = 0.00014 d^-1^  0.017 * 1/30 = 0.00057 d^-1^; 0.017 * 1/50 = 0.00034 d^-1^  0.035 * 1/30 = 0.0012 d^-1^; 0.035 * 1/50 = 0.0007 d^-1^ |
| 2 | Death rate of CD4+ RTE T-cells | CD4+ T-cell concentration in 2-year-old thymectomized child (data from *Haines2009 (Figure 3C)*) (14)  0 weeks - 370.8 cells/μL; 34 weeks – 60.4 cells/μL  Assuming exponential loss of cells: $y\left( t \right)=C*e^{-k*t}$ ==>  $C$ = 370.8 cells/μL; $k$ = 0.0076 d^-1^ – corresponds to cell loss rate |
| 3 | Transition rate of CD4+ RTE T-cells from blood to lymphatic tissue | Estimates from *vanHoeven2017* (13) (mice model)  CD4+ RTE transition time (finite-term labeling) = 3.4 d (95% CI: [2.5;4.3])  CD4+ RTE transition time (thymus transplantation experiment) = 6.8 d (95% CI: [5.5; 9.9])  According to scaling rules from *Perelson et al.* (40):  $t_{s}\sim M^{0.25}$  ($t_{s}$ – residence time; $M$ - animal weight)  Assume the same scaling rule for transition rate:  Using exponential function: $\ln\left( t_{s} \right)=0.25*\ln\left( M \right)+b$  for mouse:  mouse weight ≈ 20 g (*no information about weight and in the article vanHoeven2017, approximate* weight for *CD57/6j mice*)  finite-term labeling: $b=\ln\left( 3.4 \right)-0.25*\ln\left( 20 \right)= 0.4748$  thymus transplantation: $b=\ln\left( 6.8 \right)-0.25*\ln\left( 20 \right)= 1.1679895$  for human:  finite-term labeling: $\ln\left( t_{s} \right)=0.25*\ln\left( 70*{10}^{3} \right)+0.4748$ $= 3.26386263$ ==> $t_{s}= 26.15 h= 1.09 d$  thymus transplantation: $\ln\left( t_{s} \right)=0.25*\ln\left( 70*{10}^{3} \right)+1.1679895$ $= 3.957$==> $t_{s}= 52.3 h= 2.18 d$  **Scaled transition rates for human:**  finite-term labeling: 1/1.09 = 0.917 d^-1^  thymus transplantation: 1/2.18 = 0.459 d^-1^ |
| 4 | Death rate of cells | $death rate= \frac{ln(2)}{t_{1/2}}$ |
| 5 | Death rate of naïve cells from in vitro experimental data | estimated manually as the slope parameter in linear regression for 7AAD+ cell kinetics (data were obtained from *Figure 6A* (23)) |
| 6 | Differentiation rate of naïve to activated CD4+ T-cells | During the first 24 hours of stimulation, CD4+ and CD8+ T cells prepare for clonal expansion and increase in size, but no cell division is observed [*Kaech2002*] (26)  CD4+ T-cell division is typically delayed for another 12–24 hours (36–48 hours after the initial stimulus) [*Kaech2002*] (26)  According to *Perelson et al.* (40):  $Residence time= \frac{1}{export time}$  Assume the same equation for the time between stimuli and the onset of cell division:  $diff. rate= \frac{1}{time after stimuli}$  Differentiation rate = 1 / (36/24) = 0.67 d^-1^ (after stimuli)  Differentiation rate = 1 / (48/24) = 0.5 d^-1^ (after stimuli) |
| 7 | Differentiation rate of naïve to activated CD4+ T-cells | Differentiation rate = diff. rate (after stimuli) (calculated from *Kaech2002* (26)*,* see derivation #6) * fraction of cells which would respond to stimuli [*Ganusov and Tomura 2021*] (27)  1 in 10^5^-10^6^ of T-lymphocytes would respond to a given antigen [*Ganusov and Tomura 2021, mice*] (27) ==> fraction = 10^5^/10^10^ (total T-cells) or 10^6^/10^10^ = 0.00001 - 0.0001  CD4+: diff. rate [0.5;0.67] * fraction of responding cells [0.00001; 0.0001] = **0.000005 - 0.000067** d^-1^ |
| 8 | Differentiation rate of naïve to activated CD4+ T-cells | Estimate for *Mueller-Schoell2021* QSP model (22):  Differentiation rate (N to CM, CD4+ and CD8+ CAR-T cells) = 0.140 d^-1^  Considering CD4+/CD8+ ratio ≈ 2 [*Kulesh2025*] (1) in Lymphatic system  0.140 * 2 / 3 = **0.093** d^-1^ |
| 9 | Transition rate of CD4+ naïve, central-memory and effector-memory T-cells from lymphatic tissue to blood | Estimates from *Sprent1973* experiments (29,30)   \| Source \| Naïve CD4+ T-cells \| Memory CD4+ T-cells \| \| --- \| --- \| --- \| \| Estimates from *Sprent1973* mouse experiments (29,30) \| 1.0 d^-1^ \| 0.25 d^-1^ \| \| Estimates from *Mandl2012* (31) mouse experiments with blocked entry in LN, exponential model (*data from Figure S2 with cell egress rates*) \| 2.12 d^-1^ (Peripheral LNs)  2.83 d^-1^ (Mesenteric LNs) \|  \|   According to scaling rules from *Perelson et al.* (40):  $v_{e}\sim M^{-0.25}$  ($v_{e}$ – exit rate; $M$ - animal weight)  Assume the same scaling rule for transition rate:  Using exponential function: $\ln\left( v_{e} \right)=-0.25*\ln\left( M \right)+b$  for mouse:  mouse weight ≈ 20 g (*no information about weight and in the article Mandl2012, approximate* weight for *CD57/6j mice*)   - *Naïve CD4+ T -cells*   Exit from SLO (*Sprent1973*): $b=\ln\left( 1 \right)+0.25*\ln\left( 20 \right)= 0.748933$  Exit from SLO (*Mandl2012*, Peripheral LN): $b=\ln\left( 2.12 \right)+0.25*\ln\left( 20 \right)= 1.5$  Exit from SLO (*Mandl2012*, Mesenteric LN): $b=\ln\left( 2.83 \right)+0.25*\ln\left( 20 \right)= 1.7892$   - *Memory CD4+ T -cells*   Exit from SLO (*Sprent1973*): $b=\ln\left( 0.25 \right)+0.25*\ln\left( 20 \right)= -0.63736$  for human:   - *Naïve CD4+ T -cells*   Exit from SLO (*Sprent1973*): $\ln\left( v_{e} \right)=-0.25*\ln\left( 70*{10}^{3} \right)+0.748933= -2.04$==> $v_{e}= 0.13 d^{-1}$  Exit from SLO (*Mandl2012*, Peripheral LN): $\ln\left( v_{e} \right)=-0.25*\ln\left( 70*{10}^{3} \right)+1.5= -1.289$==> $v_{e}= 0.276 d^{-1}$  Exit from SLO (*Mandl2012*, Mesenteric LN): $\ln\left( v_{e} \right)=-0.25*\ln\left( 70*{10}^{3} \right)+1.7892= -1$==> $v_{e}= 0.368 d^{-1}$   - *Memory CD4+ T -cells*   Exit from SLO (*Sprent1973*): $\ln\left( v_{e} \right)=-0.25*\ln\left( 70*{10}^{3} \right)-0.63736= -3.42642$==> $v_{e}= 0.0325 d^{-1}$ |
| 10 | Transition rate of CD4+ naïve T-cells from lymphatic tissue to blood | According to *Ganusov and Tomura 2021* (27) mouse modeling (using *Mandl2012* (31) data):  Residence time in LN for naïve CD4+ = 12 h  According to scaling rules from *Perelson et al.* (40):  $t_{s}\sim M^{0.25}$  ($t_{s}$ – residence time; $M$ - animal weight)  Assume the same scaling rule for transition rate:  Using exponential function: $\ln\left( t_{s} \right)=0.25*\ln\left( M \right)+b$  for mouse:  mouse weight ≈ 20 g (*no information about weight and in the article Mandl2012, approximate* weight for *CD57/6j mice*)  Naïve CD4+: $b=\ln\left( 12 \right)-0.25*\ln\left( 20 \right)= 1.73597$  for human:  Naïve CD4+: $\ln\left( t_{s} \right)=0.25*\ln\left( 70*{10}^{3} \right)+1.73597= 4.525$ ==> $t_{s}= 92.3 h= 3.85 d$  $exit rate=\frac{1}{t_{s}}=\frac{1}{3.85}=\boldsymbol{0.26} d^{-1}$ |
| 11 | Proliferation rate of activated CD4+ T-cells | ~10 hours per cell division of activated cells [*Kaech2002*] (26) ==> ~ 0.4167 d per cell division  During primary infection a 10^3^-fold increase is observed for antigen-specific CD4+ T-cells in mouse [*Malhotra2020*] (32)  2*n = 10^3^ –> 10 divisions during primary infection  10 divisions = 10*10 hours = 4 days  10^3^ = exp(prolif.rate*4days) –> prolifer. rate = 1.725 d^-1^ |
| 12 | Differentiation rates of activated CD4+ T-cells | 1^st^ approach:  Human life is 30-50 times longer than mouse life (39) ==> Lifespan (human) = 30-50 * lifespan (mouse)  According to *Perelson et al.* (40):  $Residence time= \frac{1}{export time}$  Assume the same equation for lifespan:  $Lifespan= \frac{1}{diff. rate+death rate}$  Hence:  $Diff. rate \left( human \right)=\left( \frac{1}{30}or\frac{1}{50} \right)*diff. rate (mice)$  Estimates from *Gossel2017* (33) (14 week-old mouse data, model) for differentiation rates   \| Model \| N-to-CM \| N-to-EM \| CM-to-EM \| \| --- \| --- \| --- \| --- \| \| Empirical model \| 0.015 d^-1^ \| 0.01 d^-1^ \| 0.041 d^-1^ \| \| Resistant memory model \| 0.0045 d^-1^  [0.0028; 0.0087] \| 0.0017 d^-1^  [0.0011; 0.0025] \| 0.2 d^-1^  [0.13, 0.4] \| \| Declining recruitment model \| 0.0013 d^-1^  [0.00047; 0.004] \| 0.0022 d^-1^  [0.00094; 0.0058] \| 0.17 d^-1^  [0.096, 0.37] \|   **Scaled for human:**   \| Model \| N-to-CM \| N-to-EM \| CM-to-EM \| \| --- \| --- \| --- \| --- \| \| Empirical model \| 0.0003 - 0.0005 d^-1^ \| 0.0002 - 0.00033 d^-1^ \| 0.00082 - 0.00137 d^-1^ \| \| Resistant memory model \| 0.00009 - 0.00015 d^-1^ \| 0.000034 - 0.000057 d^-1^ \| 0.004 - 0.0067 d^-1^ \| \| Declining recruitment model \| 0.000026 - 0.000043 d^-1^ \| 0.000044 - 0.000073 d^-1^ \| 0.0034 - 0.0057 d^-1^ \| |
| 13 | Differentiation rates of activated CD4+ T-cells | 2^nd^ approach:  According to scaling rules from *Perelson et al.* (40):  $v_{e}\sim M^{-0.25}$  ($v_{e}$ – exit rate; $M$ - animal weight)  Assume the same scaling rule for transition rate:  Using exponential function: $\ln\left( v_{e} \right)=-0.25*\ln\left( M \right)+b$  Using estimates from *Gossel2017* (33) (14 week-old mouse data, model) for differentiation rates (see table from *derivation #12*)  for mouse:  mouse weight ≈ 27 g (*no information about weight and in the article Gossel2017, approximate* weight for 14-week-old *CD57/6j mice*)   - *CD4+: from naïve to CM*   Empirical model: $b=\ln\left( 0.015 \right)+0.25*\ln\left( 27 \right)=-3.37574586$  Resistant memory model: $b=\ln\left( 0.0045 \right)+0.25*\ln\left( 27 \right)=-4.5797$  Declining recruitment model: $b=\ln\left( 0.0013 \right)+0.25*\ln\left( 27 \right)=-5.82143$   - *CD4+: from naïve to EM*   Empirical model: $b=\ln\left( 0.01 \right)+0.25*\ln\left( 27 \right)=-3.78121$  Resistant memory model: $b=\ln\left( 0.0017 \right)+0.25*\ln\left( 27 \right)=-5.553$  Declining recruitment model: $b=\ln\left( 0.0022 \right)+0.25*\ln\left( 27 \right)=-5.2953387$   - *CD4+: from CM to EM*   Empirical model: $b=\ln\left( 0.041 \right)+0.25*\ln\left( 27 \right)=-2.37$  Resistant memory model: $b=\ln\left( 0.2 \right)+0.25*\ln\left( 27 \right)=-0.785478695933$  Declining recruitment model: $b=\ln\left( 0.17 \right)+0.25*\ln\left( 27 \right)=-0.94799762543$  for human:   - *CD4+: from naïve to CM*   Empirical model: $\ln\left( v_{e} \right)=-0.25*\ln\left( 70000 \right)-3.37574586=-6.1648$ ==> $v_{e}= 0.0021 d^{-1}$  Resistant memory model: $\ln\left( v_{e} \right)=-0.25*\ln\left( 70000 \right)-4.5797=-7.36876263$ ==> $v_{e}= 0.00063 d^{-1}$  Declining recruitment model: $\ln\left( v_{e} \right)=-0.25*\ln\left( 70000 \right)-5.82143 =-8.61$ ==> $v_{e}= 0.00018 d^{-1}$   - *CD4+: from naïve to EM*   Empirical model: $\ln\left( v_{e} \right)=-0.25*\ln\left( 70000 \right)-3.78121=-6.57$ ==> $v_{e}= 0.0014 d^{-1}$  Resistant memory model: $\ln\left( v_{e} \right)=-0.25*\ln\left( 70000 \right)-5.553=-8.342$ ==> $v_{e}= 0.00024 d^{-1}$  Declining recruitment model: $\ln\left( v_{e} \right)=-0.25*\ln\left( 70000 \right)-5.2953387 =-8.0844$ ==> $v_{e}= 0.00031 d^{-1}$   - *CD4+: from CM to EM*   Empirical model: $\ln\left( v_{e} \right)=-0.25*\ln\left( 70000 \right)-2.37=-5.159$ ==> $v_{e}= 0.0057 d^{-1}$  Resistant memory model: $\ln\left( v_{e} \right)=-0.25*\ln\left( 70000 \right)-0.785478695933=-3.0167288$ ==> $v_{e}= 0.05 d^{-1}$  Declining recruitment model: $\ln\left( v_{e} \right)=-0.25*\ln\left( 70000 \right)-0.94799762543 =-3.737$ ==> $v_{e}= 0.024 d^{-1}$ |
| 14 | Death rate of activated CD4+ T-cells | Data from ex vivo experiment in uninfected activated cells, (CD69+ cells) [*Biancotto2008*] (35):  Day 1 - 63% of cells  Day 12 - 30% of cells  Assuming linear function $y=k*x+b$ ==> $k$ = **0.028** d^-1^ – corresponds to death rate |
| 15 | Fraction of activated CD4+ T-cells, differentiated to central-memory CD4+ T-cells | According to the results obtained for activated CD4+ T-cell proliferation rates (see derivation #13)  N-to-CM proliferation rate / overall proliferation rate:  0.00018/0.00042 = 0.43  0.0021/0.0035 = 0.6 |
| 16 | Proliferation rates of central-memory and effector-memory CD4+ T-cells | Pool-average interdivision time estimates from *Gossel2017* (33) (14 week-old mouse data, model):   - For CD4+ EM cells = 88 d (83;158) - For CD4+ CM cells = 86 d (47;144)   According to scaling rules from *Perelson et al.* (40):  $t_{s}\sim M^{0.25}$  ($t_{s}$ – residence time; $M$ - animal weight)  Assume the same scaling rule for transition rate:  Using exponential function: $\ln\left( t_{s} \right)=0.25*\ln\left( M \right)+b$  for mouse:  mouse weight ≈ 27 g (*no information about weight and in the article Gossel2017, approximate* weight for 14-week-old *CD57/6j mice*)   - CD4+ EM cells:   Estimate (88 d): $b=\ln\left( 88 \right)-0.25*\ln\left( 27 \right)= 3.653377597977$  Lower limit (83 d): $b=\ln\left( 83 \right)-0.25*\ln\left( 27 \right)= 3.59488139$  Upper limit (158 d): $\ln\left( 158 \right)-0.25*\ln\left( 27 \right)= 4.2386358165$   - CD4+ CM cells:   Estimate (86 d): $b=\ln\left( 86 \right)-0.25*\ln\left( 27 \right)= 3.630388$  Lower limit (47 d): $\ln\left( 47 \right)-0.25*\ln\left( 27 \right)= 3.0261883852$  Upper limit (144 d): $\ln\left( 144 \right)-0.25*\ln\left( 27 \right)= 4.145854$  for human:   - CD4+ EM cells:   Estimate:  $\ln\left( t_{s} \right)=0.25*\ln\left( 70*{10}^{3} \right)+3.653377597977 = 6.44244$ ==> $t_{s}= 627.937 h= 26.164 d$  Lower limit:  $\ln\left( t_{s} \right)=0.25*\ln\left( 70*{10}^{3} \right)+3.59488139 = 6.383944$ ==> $t_{s}=592.26 h= 24.68 d$  Upper limit:  $\ln\left( t_{s} \right)=0.25*\ln\left( 70*{10}^{3} \right)+4.2386358165 = 7.0276984$ ==> $t_{s}=1,127.4327 h= 46.98 d$   - CD4+ CM cells:   Estimate:  $\ln\left( t_{s} \right)=0.25*\ln\left( 70*{10}^{3} \right)+3.630388 = 6.41945$ ==> $t_{s}= 613.6655 h= 25.57 d$  Lower limit:  $\ln\left( t_{s} \right)=0.25*\ln\left( 70*{10}^{3} \right)+3.0261883852 = 5.815251$ ==> $t_{s}=335.3755672 h=13.97d$  Upper limit:  $\ln\left( t_{s} \right)=0.25*\ln\left( 70*{10}^{3} \right)+4.145854 = 6.9349$ ==> $t_{s}=1,027.52 h= 42.81 d$  $division rate=\frac{1}{interdivision time}$   - CD4+ EM cells:   Estimate: 1/$26.164$ = 0.03822 d^-1^  Lower limit: 1/$24.68$ = 0.04 d^-1^  Upper limit: 1/$46.98$ = 0.0213 d^-1^   - CD4+ CM cells:   Estimate: 1/$25.57$ = 0.0391 d^-1^  Lower limit: 1/$13.97$ = 0.0716 d^-1^  Upper limit: 1/$42.81$ = 0.0234 d^-1^ |
| 17 | Transition rates of effector and effector-memory CD4+ T-cells | Estimates from *Ganusov and Tomura 2021, Sprent1976* experiments and modeling (recirculation of activated T-lymphocytes, mouse) – assumption that activated T-lymphocytes corresponds to effectors and effector-memory cells   \| **Organ** \| **Entrance from blood, h^-1^** \| **Exit to blood, h^-1^** \| \| --- \| --- \| --- \| \| Lung \| 23.64 (13.3 – 44.4) \| 1.7 (1 – 3.6) \| \| Liver \| 8.61 (5-12.8) \| 0.4 (0.3 – 0.7) \| \| Spleen \| 5.4 (2.9 – 7.7) \| 0.2 (0.2 – 0.4) \| \| MesLN \| 0.7 (0.3 – 1.6) \| 0.1 (0 – 0.8) – not identified \| \| Intestine \| 5.5 (2.9 – 7.3) \| 0.1 \|   Migration to other organs in the body = 7.5 h^-1^ (3.68 − 10.02) – from blood to tissue in our model  Exit from SLO = average exit rate for Spleen and MesLN (not identif.) ==> 0.2 (0.2 – 0.4) h^-1^  According to scaling rules from *Perelson et al.* (40):  $v_{e}\sim M^{-0.25}$  ($v_{e}$ – exit rate; $M$ - animal weight)  Assume the same scaling rule for transition rate:  Using an exponential function: $\ln\left( v_{e} \right)=-0.25*\ln\left( M \right)+b$  for mouse:  mouse weight ≈ 20 g (*no information about weight and in the article Sprent1976, approximate* weight for *CD57/6j mice*)  Migration to other organs: $b=\ln\left( 7.5 \right)+0.25*\ln\left( 20 \right)= 2.763836$  Exit from spleen: $b=\ln\left( 0.2 \right)+0.25*\ln\left( 20 \right)= -0.86$  for human:  Migration to other organs: $\ln\left( v_{e} \right)=-0.25*\ln\left( 70*{10}^{3} \right)+2.763836= -0.02522663$==> $v_{e}= 0.975 h^{-1}= \boldsymbol{23.4} d^{-1}$  Exit from spleen: $\ln\left( v_{e} \right)=-0.25*\ln\left( 70*{10}^{3} \right)-0.86= -3.649$==> $v_{e}= 0.026 h^{-1}= \boldsymbol{0.6244}d^{-1}$  Using the same scaling rule, the transition rate for CD4+ EM cells from the gastro-intestinal tract to blood was calculated:  Residence time of CD4+ EM cells in Lamina Propria = 14-25 days [*Chandler2025*] (38) ==> **0.005 – 0.01** d^-1^ transition rate for EM cells |
| 18 | Maximal carrying capacity of activated CD4+ T-cell proliferation | Number of T-lymphocytes in lymphatic system ≈ 3.6 * 10^11^ [*Sender2023*] (37)  Considering CD4+/CD8+ ratio ≈ 2 [*Kulesh2025*] (1) in lymphatic system –number of CD4+ T-cell ≈ 2.4 * 10^11^  Considering the percentage of activated HLADR+ CD38+ CD4+ T-cells in lymphoid tissue ≈ 4% [*Kulesh2025*] (1) –number of activated T-cells ≈ 10^10^  During primary infection, a 10^3^-fold increase is observed for antigen-specific CD4+ T-cells in mouse [*Malhotra2020*] – 10^13^ cells is an approximate maximal carrying capacity of lymphoid tissue |

## Supplementary Table 4

Supplementary Table 4. Homeostatic CD4+ T-lymphocyte model variables and equations

| **Variable** | **Definition** | **Equations** |
| --- | --- | --- |
| *Thymocyte dynamics sub-model: thymus involution part* | | |
| $WW\left( age \right)$, g | Thymus wet weight | $WW\left( age \right)=\left( WW_{BL} + \frac{{WW}_{max} * age}{\left( age + {WW}_{{EC50}_{1}} \right)} \right)* \left( 1- \frac{age}{age+{WW}_{{EC50}_{2}}} \right)$, (**Equation 1***)  where $\left( WW_{BL} + \frac{{WW}_{max}*age}{\left( age + {WW}_{{EC50}_{1}} \right)} \right)$ captures the increase in thymus wet weight in the first years of life; $\left( 1- \frac{age}{age+{WW}_{{EC50}_{2}}} \right)$ represents the subsequent decrease in thymus wet weight with age; $WW_{BL}$ stands for the thymus wet weight in infants (0 years-of-age) (g), ${WW}_{max}$represents the maximum increase in thymus wet weight (g); ${WW}_{{EC50}_{1}}$is the age corresponding to the 50% of the maximum increase in thymus wet weight; ${WW}_{{EC50}_{2}}$is the age corresponding to the 50% of the maximum decrease in thymus wet weight. |
| $TES\left( age \right)$, % | Relative proportion of thymic epithelial space (TES) | $TES\left( age \right)= b_{tes}*e^{-k_{tes}*age},$ (**Equation 2****)  where $k_{tes}$ (%*year^-1^) and $b_{tes}$ (%) are regression coefficients representing the slope of the relative proportion of TES and the relative proportion of TES in infants, respectively. |
| $CM\left( age \right)$, - | Cortico-medullary ratio function | $CM\left( age \right)= b_{cm}e^{{-k}_{cm}age}$, (**Equation 3****)  where $k_{cm}$ (year^-1^) and $b_{cm}$ are regression coefficients representing the slope of the cortico-medullary ratio and the cortico-medullary ratio in infants, respectively. |
| $T_{cort}^{max}\left( age \right)$, cells | Maximal thymocyte number in thymic cortex | $T_{cort}^{max}\left( age \right)= \frac{T_{0}CM\left( age \right)}{CM\left( age \right)+1}*\frac{WW\left( age \right)*TES\left( age \right)}{WW\left( 0 \right)*TES\left( 0 \right)}$, (**Equation 4****)  where $T_{0}$ is the absolute total number of thymocytes in infants (0 years-of-age); $WW\left( 0 \right)$ is thymus wet weight in infants (equals $WW_{BL}$); $TES\left( 0 \right)$ is the relative proportion of TES in infants (equals 100%). |
| $T_{med}^{max}\left( age \right)$, cells | Maximal thymocyte number in thymic medulla | $T_{med}^{max}\left( age \right)= \frac{T_{0}}{CM\left( age \right)+1}*\frac{WW\left( age \right)*TES\left( age \right)}{WW\left( 0 \right)*TES\left( 0 \right)}$, (**Equation 5****)  where $T_{0}$ is the absolute total number of thymocytes in infants (0 years-of-age); $WW\left( 0 \right)$ is thymus wet weight in infants (equals $WW_{BL}$); $TES\left( 0 \right)$ is the relative proportion of TES in infants (equals 100%). |
| *Thymocyte dynamics sub-model: thymocyte homeostasis part* | | |
| $T_{DN}$, cells | Double-negative (DN) thymocytes | $\frac{dT_{DN}}{dt}= \phi\left( 1- \frac{T_{DN}+T_{DP}}{T_{cort}^{max}\left( age \right)} \right)- \varphi_{1}T_{DN}+\lambda_{1}\left( 1- \frac{T_{DN}+T_{DP}}{T_{cort}^{max}\left( age \right)} \right)T_{DN}- \mu_{1}T_{DN}$, (**Equation 6****)  where $T_{cort}^{max}\left( age \right)$ represents an age-dependent function for the maximal thymocyte numbers (i.e., carrying capacity) in the thymic cortex; $\phi\left( 1- \frac{T_{DN}+T_{DP}}{T_{cort}^{max}\left( age \right)} \right)$ represents the inflow of thymocyte precursors; $\varphi_{1}T_{DN}$ is the differentiation rate of DN cells to DP cells; $\lambda_{1}\left( 1- \frac{T_{DN}+T_{DP}}{T_{cort}^{max}\left( age \right)} \right)T_{DN}$ represents a logistic growth function for the DN cell proliferation in the thymic cortex; $\mu_{1}T_{DN}$ represents the DN cell death; $\phi$ is the thymocyte precursor inflow rate (cells*d^-1^); $\varphi_{1}$ is the rate constant of DN-to-DP differentiation (d^-1^); $\lambda_{1}$ is the DN cell proliferation rate constant (d^-1^); and $\mu_{1}$ is the DN cell death rate constant (d^-1^). |
| $T_{DP}$, cells | Double-positive (DP) thymocytes | $\frac{dT_{DP}}{dt}= \varphi_{1}T_{DN}+\lambda_{2}\left( 1- \frac{T_{DN}+T_{DP}}{T_{cort}^{max}\left( age \right)} \right)T_{DP}-\left( \varphi_{4}+ \varphi_{8} \right)\left( 1-\frac{T_{SP4}+ T_{SP8}}{T_{med}^{max}\left( age \right)} \right)T_{DP}-\mu_{2}T_{DP}$ , (**Equation 7****)  where $T_{cort}^{max}\left( age \right)$ represents an age-dependent function for the maximal thymocyte number (i.e., carrying capacity) in the thymic cortex; $\lambda_{2}\left( 1- \frac{T_{DN}+T_{DP}}{T_{cort}^{max}\left( age \right)} \right)T_{DP}$ represents a logistic growth function for the DP cell proliferation in the thymic cortex; $T_{med}^{max}\left( age \right)$ represents an age-dependent function for the maximal thymocyte number (i.e., carrying capacity) in the thymic medulla; $\left( 1- \frac{T_{SP4}+T_{SP8}}{T_{med}^{max}\left( age \right)} \right)$ is a logistic growth function in the thymic medulla; $\varphi_{1}T_{DN}$ is the DN-to-DP differentiation; $\left( \varphi_{4}+ \varphi_{8} \right){\left( 1- \frac{T_{SP4}+T_{SP8}}{T_{med}^{max}\left( age \right)} \right)T}_{DP}$ represents the DP-to-SP4/SP8 differentiation; $\mu_{2}T_{DP}$ is the DP cell death rate; $\varphi_{1}$ is the DN-to-DP differentiation rate constant (d^-1^); $\lambda_{2}$ is the per capita DP cell proliferation rate constant (d^-1^); $\varphi_{4}$ and $\varphi_{8}$ are rate constants of DP-to-SP4 and DP-to-SP8 differentiations, respectively (d^-1^); and $\mu_{2}$ is the DP cell death rate constant (d^-1^). |
| $T_{SP4}$, cells | CD4+ Single-positive (SP4) thymocytes | $\frac{dT_{SP4}}{dt}=\varphi_{4}\left( 1- \frac{T_{SP4}+T_{SP8}}{T_{med}^{max}\left( age \right)} \right)T_{DP}-\varepsilon_{4}T_{SP4}+\lambda_{4}\left( 1-\frac{T_{SP4}+ T_{SP8}}{T_{med}^{max}\left( age \right)} \right)T_{SP4}-\mu_{4}T_{SP4}$, (**Equation 8****)  where $T_{med}^{max}\left( age \right)$ represents an age-dependent function for the maximal thymocyte number (i.e., carrying capacity) in the thymic medulla; $\varphi_{4}\left( 1- \frac{T_{SP4}+T_{SP8}}{T_{med}^{max}\left( age \right)} \right)T_{DP}$ represents the DP-to-SP4 differentiation rate constant; $\varepsilon_{4}T_{SP4}$ represents the SP4 outflux rate; $\lambda_{4}\left( 1-\frac{T_{SP4}+ T_{SP8}}{T_{med}^{max}\left( age \right)} \right)T_{SP4}$ is the logistic growth function for SP4 proliferation in the thymic medulla; $\mu_{4}T_{SP4}$ represents SP4 cell death; $\varphi_{4}$ is rate constant of DP-to-SP4 differentiation (d^-1^); $\varepsilon_{4}$ is rate constant of SP4 outflow (d^-1^); $\lambda_{4}$ is rate constant of SP4 proliferation (d^-1^); and $\mu_{4}$ is SP4 cell death rate constant (d^-1^). |
| $T_{SP8}$, cells | CD8+ Single-positive (SP8) thymocytes | $\frac{dT_{SP8}}{dt}=\varphi_{8}\left( 1- \frac{T_{SP4}+T_{SP8}}{T_{med}^{max}\left( age \right)} \right)T_{DP}-\varepsilon_{8}T_{SP8}+\lambda_{8}\left( 1-\frac{T_{SP4}+ T_{SP8}}{T_{med}^{max}\left( age \right)} \right)T_{SP8}-\mu_{8}T_{SP8}$*,* (**Equation 9****)  where $T_{med}^{max}\left( age \right)$ represents an age-dependent function for the maximal thymocyte number (i.e., carrying capacity) in the thymic medulla; $\varphi_{8}\left( 1- \frac{T_{SP4}+T_{SP8}}{T_{med}^{max}\left( age \right)} \right)T_{DP}$ represents the DP-to-SP8 differentiation rate constant; $\varepsilon_{8}T_{SP8}$ represents the SP8 outflux rate; $\lambda_{8}\left( 1-\frac{T_{SP4}+ T_{SP8}}{T_{med}^{max}\left( age \right)} \right)T_{SP8}$ is the logistic growth function for SP8 proliferation in the thymic medulla; $\mu_{8}T_{SP8}$ represents SP8 cell death; $\varphi_{8}$ is rate constant of DP-to-SP8 differentiation (d^-1^); $\varepsilon_{8}$ is rate constant of SP8 outflow (d^-1^); $\lambda_{8}$ is rate constant of SP8 proliferation (d^-1^); and $\mu_{8}$ is SP8 cell death rate constant (d^-1^). |
| *Blood volume vs age dependency* | | |
| $WT(age)$, kg | Body weight | $WT_{1}\left( age \right)=\frac{{WT}_{max1}}{\left( 1+ \frac{{TM50}_{WT1}}{age} \right)^{{hill}_{WT1}}}$, where ${hill}_{WT1}=\left\{ \begin{aligned} {hill1}_{WT1}, if age<{TM50}_{WT1} \\ {hill2}_{WT1}, if age\geq{TM50}_{WT1} \end{aligned} \right.$, (**Equation 10*****)  $WT_{2}\left( age \right)=\frac{{WT}_{max2}}{\left( 1+ \frac{{TM50}_{WT2}}{age} \right)^{{hill}_{WT2}}}$, where ${hill}_{WT2}=\left\{ \begin{aligned} {hill1}_{WT2}, if age<{TM50}_{WT2} \\ {hill2}_{WT2}, if age\geq{TM50}_{WT2} \end{aligned} \right.$, (**Equation 11*****)  $WT_{3}\left( age \right)=\frac{{WT}_{max3}}{\left( 1+ \frac{{TM50}_{WT3}}{age} \right)^{{hill}_{WT3}}}$, where ${hill}_{WT3}=\left\{ \begin{aligned} {hill1}_{WT3}, if age<{TM50}_{WT3} \\ {hill2}_{WT3}, if age\geq{TM50}_{WT3} \end{aligned} \right.$, (**Equation 12*****)  $WT_{4}\left( age \right)=\left\{ \begin{aligned} 0, if age\leq{TM50}_{WT3} \\ {WT}_{max4}*\left( 1- e^{- \frac{\ln\left( 2 \right)}{THALF_{WT4}}*\left( age- {TM50}_{WT3} \right)} \right), if age>{TM50}_{WT3} \end{aligned} \right.$ , (**Equation 13*****) #years  $WT\left( age \right)= WT_{1}+ WT_{2}+WT_{3}+ WT_{4}$, (**Equation 14*****)  where $WT_{1}\left( age \right)$, $WT_{2}\left( age \right)$, $WT_{3}\left( age \right)$, $WT_{4}\left( age \right)$ are four components ($WT_{1}$, $WT_{2}$, $WT_{3}$ – asymmetrical sigmoid hyperbolic functions; $WT_{4}$ – exponential function with a lag time) capturing the staged non-linear age dependency of body weight; ${WT}_{max1}$, ${WT}_{max2}$, ${WT}_{max3}$ and ${WT}_{max4}$ represent the maximum increase in body weight sigmoid functions for each component, respectively (kg); ${TM50}_{WT1}$, ${TM50}_{WT2}$, ${TM50}_{WT3}$ are the age corresponding to the 50% of the maximum increase in body weight for each component, respectively; ${hill1}_{WT1}$, ${hill2}_{WT1}$, ${hill1}_{WT2}$, ${hill2}_{WT2}$, ${hill1}_{WT3}$, ${hill2}_{WT3}$ are Hill coefficients; $THALF_{WT4}$ is the age half-life for $WT_{4}$component. |
| $BV(age)$, mL | Blood volume | $BV\left( age \right)=BV_{BL}+k_{BV}*WT(age)$, (**Equation 15**)  where $BV_{BL}$ (mL) and $k_{BV}$(mL/kg) are regression coefficients representing the intercept and slope of the blood volume dependency on body weight, respectively. |
| CD4+ T-lymphocyte cellular kinetics sub-model (blood, lymphatic tissue, gastro-intestinal tract and lungs) | | |
| $T_{RTE4}^{BL}$, cells | CD4+ RTE T-lymphocytes in blood (cell count) | $\frac{d T_{RTE4}^{BL}}{dt}=\varepsilon_{4}T_{SP4} -\mu_{RTE4}T_{RTE4}^{BL}-\omega_{RTE4_{bl-lt}}T_{RTE4}^{BL}$, (**Equation 16**)  where $\varepsilon_{4}T_{SP4}$ represents the SP4 outflux rate; $\mu_{RTE4}T_{RTE4}^{BL}$ represents RTE CD4+ T-cell death in blood; $\omega_{RTE4_{bl-lt}}T_{RTE4}^{BL}$ represents the migration rate of RTE CD4+ T-cells from blood to lymphatic tissue; $\mu_{RTE4}$ is RTE CD4+ T-cell death rate constant (d^-1^); $\omega_{RTE4_{bl-lt}}$ is RTE CD4+ T-cell migration from blood to lymphatic tissue rate constant (d^-1^). |
| $T_{RTE4}^{LT}$, cells | CD4+ RTE T-lymphocytes in lymphatic tissue (cell count) | $\frac{d T_{RTE4}^{LT}}{dt}=\omega_{RTE4_{bl-lt}}T_{RTE4}^{BL}-\mu_{RTE4}T_{RTE4}^{LT}-\varphi_{RTE4}T_{RTE4}^{LT}$, (**Equation 17**)  where $\omega_{RTE4_{bl-lt}}T_{RTE4}^{BL}$ represents migration rate of RTE CD4+ T-cells from blood to lymphatic tissue; $\mu_{RTE4}T_{RTE4}^{LT}$ represents RTE CD4+ T-cell death in lymphatic tissue; $\varphi_{RTE4}T_{RTE4}^{LT}$ represents the differentiation rate of RTE to naive CD4+ T-cells; $\omega_{RTE4_{bl-lt}}$ is RTE CD4+ T-cell migration from blood to lymphatic tissue rate (d^-1^); $\mu_{RTE4}$ is RTE CD4+ T-cell death rate constant (d^-1^); $\varphi_{RTE4}$ is RTE CD4+ T-cell differentiation rate constant (d^-1^). |
| $T_{N4}^{LT}$, cells | CD4+ naïve T-lymphocytes in lymphatic tissue (cell count) | $\frac{d T_{N4}^{LT}}{dt}=\varphi_{RTE4}T_{RTE4}^{LT}-\mu_{N4}T_{N4}^{LT}+\lambda_{N4}T_{N4}^{LT}-{\omega_{N4}}_{lt-bl}T_{N4}^{LT}+{\omega_{N4}}_{bl-lt}T_{N4}^{BL}-\varphi_{N4}T_{N4}^{LT}$, (**Equation 18**)  where $\varphi_{RTE4}T_{RTE4}^{LT}$ represents the differentiation rate of RTE to naive CD4+ T-cells; $\mu_{N4}T_{N4}^{LT}$ represents naïve CD4+ T-cell death in lymphatic tissue; $\lambda_{N4}T_{N4}^{LT}$ represents the proliferation rate of naïve CD4+ T-cells; ${\omega_{N4}}_{lt-bl}T_{N4}^{LT}$ represents the migration of naïve CD4+ T-cells from lymphoid tissue to blood; ${\omega_{N4}}_{bl-lt}T_{N4}^{BL}$ represents the migration of naïve CD4+ T-cells from blood to lymphoid tissue; $\varphi_{N4}T_{N4}^{LT}$ represents the differentiation rate of naïve to activated CD4+ T-cells; $\varphi_{RTE4}$ is the RTE CD4+ T-cell differentiation rate constant (d^-1^); $\mu_{N4}$ is the naïve CD4+ T-cell death rate constant (d^-1^); $\lambda_{N4}$ is the naïve CD4+ T-cell proliferation rate constant (d^-1^); ${\omega_{N4}}_{lt-bl}$ is the naïve CD4+ T-cell transition from lymphoid tissue to blood rate (d^-1^); ${\omega_{N4}}_{bl-lt}$ is the naïve CD4+ T-cell transition from blood to lymphoid tissue rate (d^-1^); and $\varphi_{N4}$ is the naïve CD4+ T-cell differentiation rate constant (d^-1^). |
| $T_{N4}^{BL}$, cells | CD4+ naïve T-lymphocytes in blood (cell count) | $\frac{d T_{N4}^{BL}}{dt}={\omega_{N4}}_{lt-bl}T_{N4}^{LT}-{\omega_{N4}}_{bl-lt}T_{N4}^{BL}+{\omega_{N4}}_{git-bl}T_{N4}^{GIT}-{\omega_{N4}}_{bl-git}T_{N4}^{BL}+{\omega_{N4}}_{lung-bl}T_{N4}^{Lung}-{\omega_{N4}}_{bl-lung}T_{N4}^{BL}-{\omega_{N4}}_{bl-tis}T_{N4}^{BL}-\mu_{N4}T_{N4}^{BL}$*,* (**Equation 19**)  where ${\omega_{N4}}_{lt-bl}T_{N4}^{LT}$ represents the migration of naïve CD4+ T-cells from lymphoid tissue to blood; ${\omega_{N4}}_{bl-lt}T_{N4}^{BL}$ represents the migration of naïve CD4+ T-cells from blood to lymphoid tissue; ${\omega_{N4}}_{git-bl}T_{N4}^{GIT}$ represents the migration of naïve CD4+ T-cells from gastro-intestinal tract to blood; ${\omega_{N4}}_{bl-git}T_{N4}^{BL}$ represents the migration of naïve CD4+ T-cells from blood to gastro-intestinal tract; ${\omega_{N4}}_{lung-bl}T_{N4}^{LUNG}$ represents the migration of naïve CD4+ T-cells from lungs to blood; ${\omega_{N4}}_{bl-lung}T_{N4}^{BL}$ represents the migration of naïve CD4+ T-cells from blood to lungs; ${\omega_{N4}}_{bl-tis}T_{N4}^{BL}$ represents the migration of naïve CD4+ T-cells from blood to other peripheral tissues; $\mu_{N4}T_{N4}^{BL}$ represents the naïve CD4+ T-cell death in blood; ${\omega_{N4}}_{lt-bl}$ is the naïve CD4+ T-cell transition from lymphoid tissue to blood rate (d^-1^); ${\omega_{N4}}_{bl-lt}$ is the naïve CD4+ T-cell transition from blood to lymphoid tissue rate constant (d^-1^); ${\omega_{N4}}_{git-bl}$ is the naïve CD4+ T-cell transition from gastro-intestinal tract to blood rate (d^-1^); ${\omega_{N4}}_{bl-git}$ is the naïve CD4+ T-cell transition from blood to gastro-intestinal tract rate (d^-1^); ${\omega_{N4}}_{lung-bl}$ is the naïve CD4+ T-cell transition from lungs to blood rate (d^-1^); ${\omega_{N4}}_{bl-lung}$ is the naïve CD4+ T-cell transition from blood to lungs rate (d^-1^); $\mu_{N4}$ is the naïve CD4+ T-cell death rate constant (d^-1^). |
| $T_{N4}^{GIT}$, cells | CD4+ naïve T-lymphocytes in gastro-intestinal tract (cell count) | $\frac{d T_{N4}^{GIT}}{dt}={\omega_{N4}}_{bl-git}T_{N4}^{BL}-{\omega_{N4}}_{git-bl}T_{N4}^{GIT}-\mu_{N4}T_{N4}^{GIT}$*,* (**Equation 20**)  where ${\omega_{N4}}_{git-bl}T_{N4}^{GIT}$ represents the migration of naïve CD4+ T-cells from gastro-intestinal tract to blood; ${\omega_{N4}}_{bl-git}T_{N4}^{BL}$ represents the migration of naïve CD4+ T-cells from blood to the gastro-intestinal tract; $\mu_{N4}T_{N4}^{GIT}$ represents the naïve CD4+ T-cell death in the gastro-intestinal tract; ${\omega_{N4}}_{git-bl}$ is the naïve CD4+ T-cell transition from gastro-intestinal tract to blood rate (d^-1^); ${\omega_{N4}}_{bl-git}$ is the naïve CD4+ T-cell transition from blood to gastro-intestinal tract rate (d^-1^); $\mu_{N4}$ is the naïve CD4+ T-cell death rate constant (d^-1^). |
| $T_{N4}^{LUNG}$, cells | CD4+ naïve T-lymphocytes in lungs (cell count) | $\frac{d T_{N4}^{LUNG}}{dt}={\omega_{N4}}_{bl-lung}T_{N4}^{LUNG}-{\omega_{N4}}_{lung-bl}T_{N4}^{LUNG}-\mu_{N4}T_{N4}^{LUNG}$*,* (**Equation 21**)  where ${\omega_{N4}}_{lung-bl}T_{N4}^{LUNG}$ represents the migration of naïve CD4+ T-cells from lungs to blood; ${\omega_{N4}}_{bl-lung}T_{N4}^{BL}$ represents the migration of naïve CD4+ T-cells from blood to lungs; $\mu_{N4}T_{N4}^{LUNG}$ represents the naïve CD4+ T-cell death in lungs; ${\omega_{N4}}_{lung-bl}$ is the naïve CD4+ T-cell transition from lungs to blood rate (d^-1^); ${\omega_{N4}}_{bl-lung}$ is the naïve CD4+ T-cell transition from blood to lungs rate (d^-1^); $\mu_{N4}$ is the naïve CD4+ T-cell death rate constant (d^-1^). |
| $T_{A4}^{LT}$, cells | CD4+ activated T-lymphocytes in lymphatic tissue (cell count) | $\frac{d T_{A4}^{LT}}{dt}=\varphi_{N4}T_{N4}^{LT}-\mu_{A4}T_{A4}^{LT}+\lambda_{A4}T_{A4}^{LT}\left( 1- \frac{T_{A4}^{LT}}{T_{{A4}_{max}}} \right)-{\omega_{A4}}_{lt-bl}T_{A4}^{LT}+{\omega_{A4}}_{bl-lt}T_{A4}^{BL}-\varphi_{A4}T_{A4}^{LT}$, (**Equation 22**)  where $\varphi_{N4}T_{N4}^{LT}$ represents the differentiation rate of naïve to activated CD4+ T-cells; $\mu_{A4}T_{A4}^{LT}$ represents the activated CD4+ T-cell death in lymphatic tissue; $\lambda_{A4}T_{A4}^{LT}\left( 1- \frac{T_{A4}^{LT}}{T_{{A4}_{max}}} \right)$ represents the logistic growth function for the activated CD4+ T-cell proliferation; ${\omega_{A4}}_{lt-bl}T_{A4}^{LT}$ represents the migration of activated CD4+ T-cells from lymphoid tissue to blood; ${\omega_{A4}}_{bl-lt}T_{A4}^{BL}$ represents the migration of activated CD4+ T-cells from blood to lymphoid tissue; $\varphi_{A4}T_{A4}^{LT}$ represents the differentiation rate of activated to memory and effector CD4+ T-cells; $\varphi_{N4}$ is the naïve CD4+ T-cell differentiation rate constant (d^-1^); $\mu_{A4}$ is the activated CD4+ T-cell death rate constant (d^-1^); $\lambda_{A4}$ is the activated CD4+ T-cell proliferation rate constant (d^-1^); $T_{{A4}_{max}}$ is the maximal activated cell number (i.e., carrying capacity for clonal expansion) (cells); ${\omega_{A4}}_{lt-bl}$ is the activated CD4+ T-cell transition from lymphoid tissue to blood rate (d^-1^); ${\omega_{A4}}_{bl-lt}$ is the activated CD4+ T-cell transition from blood to lymphoid tissue rate (d^-1^); $\varphi_{A4}$ is the activated CD4+ T-cell differentiation rate constant (d^-1^). |
| $T_{A4}^{BL}$, cells | CD4+ activated T-lymphocytes in blood (cell count) | $\frac{d T_{A4}^{BL}}{dt}={\omega_{A4}}_{lt-bl}T_{A4}^{LT}-{\omega_{A4}}_{bl-lt}T_{A4}^{BL}- {\omega_{A4}}_{bl-tis}T_{A4}^{BL}-\mu_{A4}T_{A4}^{BL}$, (**Equation 23**)  where ${\omega_{A4}}_{lt-bl}T_{A4}^{LT}$ represents the migration of activated CD4+ T-cells from lymphoid tissue to blood; ${\omega_{A4}}_{bl-lt}T_{A4}^{BL}$ represents the migration of activated CD4+ T-cells from blood to lymphoid tissue; ${\omega_{A4}}_{bl-tis}T_{A4}^{BL}$ represents the migration of activated CD4+ T-cells from blood to other peripheral tissues; $\mu_{A4}T_{A4}^{BL}$ represents the activated CD4+ T-cell death in blood; ${\omega_{A4}}_{lt-bl}$ is the activated CD4+ T-cell transition from lymphoid tissue to blood rate (d^-1^); ${\omega_{A4}}_{bl-lt}$ is the activated CD4+ T-cell transition from blood to lymphoid tissue rate (d^-1^); ${\omega_{A4}}_{bl-tis}$ is the activated CD4+ T-cell transition from blood to other peripheral tissues rate (d^-1^); $\mu_{A4}$ is the activated CD4+ T-cell death rate constant (d^-1^). |
| $T_{CM4}^{LT}$, cells | CD4+ central-memory T-lymphocytes in lymphatic tissue (cell count) | $\frac{d T_{CM4}^{LT}}{dt}=f_{4}\varphi_{A4}T_{A4}^{LT}-\mu_{CM4}T_{CM4}^{LT}+\lambda_{CM4}T_{CM4}^{LT}-{\omega_{CM4}}_{lt-bl}T_{CM4}^{LT}+{\omega_{CM4}}_{bl-lt}T_{CM4}^{BL}-\varphi_{CM4}T_{CM4}^{LT}$, (**Equation 24**)  where $f_{4}\varphi_{A4}T_{A4}^{LT}$ represents the differentiation rate of activated to central-memory CD4+ T-cells; $\mu_{CM4}T_{CM4}^{LT}$ represents the central-memory CD4+ T-cell death in lymphatic tissue; $\lambda_{CM4}T_{CM4}^{LT}$ represents the proliferation rate of central-memory CD4+ T-cells; ${\omega_{CM4}}_{lt-bl}T_{CM4}^{LT}$ represents the migration of central-memory CD4+ T-cells from lymphoid tissue to blood; ${\omega_{CM4}}_{bl-lt}T_{CM4}^{BL}$ represents the migration of central-memory CD4+ T-cells from blood to lymphoid tissue; $\varphi_{CM4}T_{CM4}^{LT}$ represents the differentiation rate of central-memory to effector-memory CD4+ T-cells; $\varphi_{A4}$ is the activated CD4+ T-cell differentiation rate constant (d^-1^); $f_{4}$ is the fraction of activated CD4+ T-cells, differentiated to central-memory vs. effector CD4+ T-cells; $\mu_{CM4}$ is the central-memory CD4+ T-cell death rate constant (d^-1^); $\lambda_{CM4}$ is the central-memory CD4+ T-cell proliferation rate constant (d^-1^); ${\omega_{CM4}}_{lt-bl}$ is the central-memory CD4+ T-cell transition from lymphoid tissue to blood rate (d^-1^); ${\omega_{CM4}}_{bl-lt}$ is the central-memory CD4+ T-cell transition from blood to lymphoid tissue rate (d^-1^); $\varphi_{CM4}$ is the central-memory CD4+ T-cell differentiation rate constant (d^-1^). |
| $T_{CM4}^{BL}$, cells | CD4+ central-memory T-lymphocytes in blood (cell count) | $\frac{d T_{CM4}^{BL}}{dt}={\omega_{CM4}}_{lt-bl}T_{CM4}^{LT}-{\omega_{CM4}}_{bl-lt}T_{CM4}^{BL}+{\omega_{CM4}}_{git-bl}T_{CM4}^{GIT}-{\omega_{CM4}}_{bl-git}T_{CM4}^{BL}+{\omega_{CM4}}_{lung-bl}T_{CM4}^{Lung}-{\omega_{CM4}}_{bl-lung}T_{CM4}^{BL}-{\omega_{CM4}}_{bl-tis}T_{CM4}^{BL}- \mu_{CM4}T_{CM4}^{BL}$*,* (**Equation 25**)  where ${\omega_{CM4}}_{lt-bl}T_{CM4}^{LT}$ represents the migration of central-memory CD4+ T-cells from lymphoid tissue to blood; ${\omega_{CM4}}_{bl-lt}T_{CM4}^{BL}$ represents the migration of central-memory CD4+ T-cells from blood to lymphoid tissue; ${\omega_{CM4}}_{git-bl}T_{CM4}^{GIT}$ represents the migration of central-memory CD4+ T-cells from gastro-intestinal tract to blood; ${\omega_{CM4}}_{bl-git}T_{CM4}^{BL}$ represents the migration of central-memory CD4+ T-cells from blood to gastro-intestinal tract; ${\omega_{CM4}}_{lung-bl}T_{CM4}^{LUNG}$ represents the migration of central-memory CD4+ T-cells from lungs to blood; ${\omega_{CM4}}_{bl-lung}T_{CM4}^{BL}$ represents the migration of central-memory CD4+ T-cells from blood to lungs; ${\omega_{CM4}}_{bl-tis}T_{CM4}^{BL}$ represents the migration of central-memory CD4+ T-cells from blood to other peripheral tissues; $\mu_{CM4}T_{CM4}^{BL}$ represents the central-memory CD4+ T-cell death in blood; ${\omega_{CM4}}_{lt-bl}$ is the central-memory CD4+ T-cell transition from lymphoid tissue to blood rate constant (d^-1^); ${\omega_{CM4}}_{bl-lt}$ is the central-memory CD4+ T-cell transition from blood to lymphoid tissue rate (d^-1^); ${\omega_{CM4}}_{git-bl}$ is the central-memory CD4+ T-cell transition from gastro-intestinal tract to blood rate (d^-1^); ${\omega_{CM4}}_{bl-git}$ is the central-memory CD4+ T-cell transition from blood to gastro-intestinal tract rate (d^-1^); ${\omega_{CM4}}_{lung-bl}$ is the central-memory CD4+ T-cell transition from lungs to blood rate (d^-1^); ${\omega_{CM4}}_{bl-lung}$ is the central-memory CD4+ T-cell transition from blood to lungs rate (d^-1^); $\mu_{CM4}$ is the central-memory CD4+ T-cell death rate constant (d^-1^). |
| $T_{CM4}^{GIT}$, cells | CD4+ central-memory T-lymphocytes in gastro-intestinal tract (cell count) | $\frac{d T_{CM4}^{GIT}}{dt}={\omega_{CM4}}_{bl-git}T_{CM4}^{BL}-{\omega_{CM4}}_{git-bl}T_{CM4}^{GIT}-\mu_{CM4}T_{CM4}^{GIT}$*,* (**Equation 26**)  where ${\omega_{CM4}}_{git-bl}T_{CM4}^{GIT}$ represents the migration of central-memory CD4+ T-cells from gastro-intestinal tract to blood; ${\omega_{CM4}}_{bl-git}T_{CM4}^{BL}$ represents the migration of central-memory CD4+ T-cells from blood to gastro-intestinal tract; $\mu_{CM4}T_{CM4}^{GIT}$ represents the central-memory CD4+ T-cell death in gastro-intestinal tract; ${\omega_{CM4}}_{git-bl}$ is the central-memory CD4+ T-cell transition from gastro-intestinal tract to blood rate (d^-1^); ${\omega_{CM4}}_{bl-git}$ is the central-memory CD4+ T-cell transition from blood to gastro-intestinal tract rate (d^-1^); $\mu_{CM4}$ is the central-memory CD4+ T-cell death rate constant (d^-1^). |
| $T_{CM4}^{LUNG}$, cells | CD4+ central-memory T-lymphocytes in lungs (cell count) | $\frac{d T_{CM4}^{LUNG}}{dt}={\omega_{CM4}}_{bl-lung}T_{CM4}^{LUNG}-{\omega_{CM4}}_{lung-bl}T_{CM4}^{LUNG}-\mu_{CM4}T_{CM4}^{LUNG}$*,* (**Equation 27**)  where ${\omega_{CM4}}_{lung-bl}T_{CM4}^{LUNG}$ represents the migration of central-memory CD4+ T-cells from lungs to blood; ${\omega_{CM4}}_{bl-lung}T_{CM4}^{BL}$ represents the migration of central-memory CD4+ T-cells from blood to lungs; $\mu_{CM4}T_{CM4}^{LUNG}$ represents the central-memory CD4+ T-cell death in lungs; ${\omega_{CM4}}_{lung-bl}$ is the central-memory CD4+ T-cell transition from lungs to blood rate (d^-1^); ${\omega_{CM4}}_{bl-lung}$ is the central-memory CD4+ T-cell transition from blood to lungs rate (d^-1^); $\mu_{CM4}$ is the central-memory CD4+ T-cell death rate constant (d^-1^). |
| $T_{EM4}^{LT}$, cells | CD4+ effector-memory T-lymphocytes in lymphatic tissue (cell count) | $\frac{d T_{EM4}^{LT}}{dt}=\varphi_{CM4}T_{CM4}^{LT}-\mu_{EM4}T_{EM4}^{LT}+\lambda_{EM4}T_{EM4}^{LT}-{\omega_{EM4}}_{lt-bl}T_{EM4}^{LT}-\varphi_{EM4}T_{EM4}^{LT}$, (**Equation 28**)  where $\varphi_{CM4}T_{CM4}^{LT}$ represents the differentiation rate of central-memory to effector-memory CD4+ T-cells; $\mu_{EM4}T_{EM4}^{LT}$ represents the effector-memory CD4+ T-cell death in lymphatic tissue; $\lambda_{EM4}T_{EM4}^{LT}$ represents the proliferation rate of effector-memory CD4+ T-cells; ${\omega_{EM4}}_{lt-bl}T_{EM4}^{LT}$ represents the migration of effector-memory CD4+ T-cells from lymphoid tissue to blood; $\varphi_{EM4}T_{EM4}^{LT}$ represents the differentiation rate of effector-memory to effector CD4+ T-cells; $\varphi_{CM4}$ is the central-memory CD4+ T-cell differentiation rate constant (d^-1^); $\mu_{EM4}$ is the effector-memory CD4+ T-cell death rate constant (d^-1^); $\lambda_{EM4}$ is the effector-memory CD4+ T-cell proliferation rate constant (d^-1^); ${\omega_{EM4}}_{lt-bl}$ is the effector-memory CD4+ T-cell transition from lymphoid tissue to blood rate (d^-1^); $\varphi_{EM4}$ is the effector-memory CD4+ T-cell differentiation rate constant (d^-1^). |
| $T_{EM4}^{BL}$, cells | CD4+ effector-memory T-lymphocytes in blood (cell count) | $\frac{d T_{EM4}^{BL}}{dt}={\omega_{EM4}}_{lt-bl}T_{EM4}^{LT}+{\omega_{EM4}}_{git-bl}T_{EM4}^{GIT}-{\omega_{EM4}}_{bl-git}T_{EM4}^{BL}+{\omega_{EM4}}_{lung-bl}T_{EM4}^{Lung}-{\omega_{EM4}}_{bl-lung}T_{EM4}^{BL}-{\omega_{EM4}}_{bl-tis}T_{EM4}^{BL}- \mu_{EM4}T_{EM4}^{BL}$*,* (**Equation 29**)  where ${\omega_{EM4}}_{lt-bl}T_{EM4}^{LT}$ represents the migration of effector-memory CD4+ T-cells from lymphoid tissue to blood; ${\omega_{EM4}}_{git-bl}T_{EM4}^{GIT}$ represents the migration of effector-memory CD4+ T-cells from gastro-intestinal tract to blood; ${\omega_{EM4}}_{bl-git}T_{EM4}^{BL}$ represents the migration of effector-memory CD4+ T-cells from blood to gastro-intestinal tract; ${\omega_{EM4}}_{lung-bl}T_{EM4}^{LUNG}$ represents the migration of effector-memory CD4+ T-cells from lungs to blood; ${\omega_{EM4}}_{bl-lung}T_{EM4}^{BL}$ represents the migration of effector-memory CD4+ T-cells from blood to lungs; ${\omega_{EM4}}_{bl-tis}T_{EM4}^{BL}$ represents the migration of effector-memory CD4+ T-cells from blood to other peripheral tissues; $\mu_{EM4}T_{EM4}^{BL}$ represents the effector-memory CD4+ T-cell death in blood; ${\omega_{EM4}}_{lt-bl}$ is the effector-memory CD4+ T-cell transition from lymphoid tissue to blood rate (d^-1^); ${\omega_{EM4}}_{git-bl}$ is the effector-memory CD4+ T-cell transition from gastro-intestinal tract to blood rate (d^-1^); ${\omega_{EM4}}_{bl-git}$ is the effector-memory CD4+ T-cell transition from blood to gastro-intestinal tract rate (d^-1^); ${\omega_{EM4}}_{lung-bl}$ is the effector-memory CD4+ T-cell transition from lungs to blood rate (d^-1^); ${\omega_{EM4}}_{bl-lung}$ is the effector-memory CD4+ T-cell transition from blood to lungs rate (d^-1^); $\mu_{EM4}$ is the effector-memory CD4+ T-cell death rate constant (d^-1^). |
| $T_{EM4}^{GIT}$, cells | CD4+ effector-memory T-lymphocytes in gastro-intestinal tract (cell count) | $\frac{d T_{EM4}^{GIT}}{dt}={\omega_{EM4}}_{bl-git}T_{EM4}^{BL}-{\omega_{EM4}}_{git-bl}T_{EM4}^{GIT}-\mu_{EM4}T_{EM4}^{GIT}$*,* (**Equation 30**)  where ${\omega_{EM4}}_{git-bl}T_{EM4}^{GIT}$ represents the migration of effector-memory CD4+ T-cells from gastro-intestinal tract to blood; ${\omega_{EM4}}_{bl-git}T_{EM4}^{BL}$ represents the migration of effector-memory CD4+ T-cells from blood to gastro-intestinal tract; $\mu_{EM4}T_{EM4}^{GIT}$ represents the effector-memory CD4+ T-cell death in gastro-intestinal tract; ${\omega_{EM4}}_{git-bl}$ is the effector-memory CD4+ T-cell transition from gastro-intestinal tract to blood rate (d^-1^); ${\omega_{EM4}}_{bl-git}$ is the effector-memory CD4+ T-cell transition from blood to gastro-intestinal tract rate (d^-1^); $\mu_{EM4}$ is the effector-memory CD4+ T-cell death rate constant (d^-1^). |
| $T_{EM4}^{LUNG}$, cells | CD4+ effector-memory T-lymphocytes in lungs (cell count) | $\frac{d T_{EM4}^{LUNG}}{dt}={\omega_{EM4}}_{bl-lung}T_{EM4}^{LUNG}-{\omega_{EM4}}_{lung-bl}T_{EM4}^{LUNG}-\mu_{EM4}T_{EM4}^{LUNG}$*,* (**Equation 31**)  where ${\omega_{EM4}}_{lung-bl}T_{EM4}^{LUNG}$ represents the migration of effector-memory CD4+ T-cells from lungs to blood; ${\omega_{EM4}}_{bl-lung}T_{EM4}^{BL}$ represents the migration of effector-memory CD4+ T-cells from blood to lungs; $\mu_{EM4}T_{EM4}^{LUNG}$ represents the effector-memory CD4+ T-cell death in lungs; ${\omega_{EM4}}_{lung-bl}$ is the effector-memory CD4+ T-cell transition from lungs to blood rate constant (d^-1^); ${\omega_{EM4}}_{bl-lung}$ is the effector-memory CD4+ T-cell transition from blood to lungs rate (d^-1^); $\mu_{EM4}$ is the effector-memory CD4+ T-cell death rate constant (d^-1^). |
| $T_{EFF4}^{LT}$, cells | CD4+ effector T-lymphocytes in lymphatic tissue (cell count) | $\frac{d T_{EFF4}^{LT}}{dt}=\varphi_{EM4}T_{EM4}^{LT}+\left( 1- f_{4} \right) \varphi_{A4}T_{A4}^{LT}-\mu_{EFF4}T_{EFF4}^{LT}+-{\omega_{EFF4}}_{lt-bl}T_{EFF4}^{LT}$, (**Equation 32**)  where $\varphi_{EM4}T_{EM4}^{LT}$ represents the differentiation rate of effector-memory to effector CD4+ T-cells; $\left( 1- f_{4} \right) \varphi_{A4}T_{A4}^{LT}$ represents the differentiation rate of activated to effector CD4+ T-cells; $\mu_{EFF4}T_{EFF4}^{LT}$ represents the effector CD4+ T-cell death in lymphatic tissue; ${\omega_{EFF4}}_{lt-bl}T_{EFF4}^{LT}$ represents the migration of effector CD4+ T-cells from lymphoid tissue to blood; $\varphi_{EM4}$ is the effector-memory CD4+ T-cell differentiation rate constant (d^-1^); $\varphi_{A4}$ is the activated CD4+ T-cell differentiation rate constant (d^-1^); $f_{4}$ is the fraction of activated CD4+ T-cells, differentiated to central-memory vs. effector CD4+ T-cells; $\mu_{EFF4}$ is the effector CD4+ T-cell death rate constant (d^-1^); ${\omega_{EFF4}}_{lt-bl}$ is the effector CD4+ T-cell transition from lymphoid tissue to blood rate (d^-1^). |
| $T_{EFF4}^{BL}$, cells | CD4+ effector T-lymphocytes in blood (cell count) | $\frac{d T_{EFF4}^{BL}}{dt}={\omega_{EFF4}}_{lt-bl}T_{EFF4}^{LT}-{\omega_{EFF4}}_{bl-git}T_{EFF4}^{BL}-{\omega_{EFF4}}_{bl-lung}T_{EFF4}^{BL}-{\omega_{EFF4}}_{bl-tis}T_{EFF4}^{BL}- \mu_{EFF4}T_{EFF4}^{BL}$*,* (**Equation 33**)  where ${\omega_{EFF4}}_{lt-bl}T_{EFF4}^{LT}$ represents the migration of effector CD4+ T-cells from lymphoid tissue to blood; ${\omega_{EFF4}}_{bl-git}T_{EFF4}^{BL}$ represents the migration of effector CD4+ T-cells from blood to gastro-intestinal tract; ${\omega_{EFF4}}_{bl-lung}T_{EFF4}^{BL}$ represents the migration of effector CD4+ T-cells from blood to lungs; ${\omega_{EFF4}}_{bl-tis}T_{EFF4}^{BL}$ represents the migration of effector CD4+ T-cells from blood to other peripheral tissues; $\mu_{EFF4}T_{EFF4}^{BL}$ represents the effector CD4+ T-cell death in blood; ${\omega_{EFF4}}_{lt-bl}$ is the effector CD4+ T-cell transition from lymphoid tissue to blood rate (d^-1^); ${\omega_{EFF4}}_{bl-git}$ is the effector CD4+ T-cell transition from blood to gastro-intestinal tract rate (d^-1^); ${\omega_{EFF4}}_{bl-lung}$ is the effector CD4+ T-cell transition from blood to lungs rate (d^-1^); $\mu_{EFF4}$ is the effector CD4+ T-cell death rate constant (d^-1^). |
| $T_{EFF4}^{GIT}$, cells | CD4+ effector T-lymphocytes in gastro-intestinal tract (cell count) | $\frac{d T_{EFF4}^{GIT}}{dt}={\omega_{EFF4}}_{bl-git}T_{EFF4}^{BL}-\mu_{EFF4}T_{EFF4}^{GIT}$*,* (**Equation 34**)  where ${\omega_{EFF4}}_{bl-git}T_{EFF4}^{BL}$ represents the migration of effector CD4+ T-cells from blood to gastro-intestinal tract; $\mu_{EFF4}T_{EFF4}^{GIT}$ represents the effector CD4+ T-cell death in blood; ${\omega_{EFF4}}_{bl-git}$ is the effector CD4+ T-cell transition from blood to gastro-intestinal tract rate (d^-1^); $\mu_{EFF4}$ is the effector CD4+ T-cell death rate constant (d^-1^). |
| $T_{EFF4}^{LUNG}$, cells | CD4+ effector T-lymphocytes in lungs (cell count) | $\frac{d T_{EFF4}^{LUNG}}{dt}={\omega_{EFF4}}_{bl-lung}T_{EFF4}^{LUNG}-\mu_{EFF4}T_{EFF4}^{LUNG}$*,* (**Equation 35**)  where ${\omega_{EFF4}}_{bl-lung}T_{EFF4}^{LUNG}$ represents the migration of effector CD4+ T-cells from blood to lungs; $\mu_{EFF4}T_{EFF4}^{LUNG}$ represents the effector CD4+ T-cell death in blood; ${\omega_{EFF4}}_{bl-lung}$ is the effector CD4+ T-cell transition from blood to lungs rate (d^-1^); $\mu_{EFF4}$ is the effector CD4+ T-cell death rate constant (d^-1^). |
| $T_{{RTE4}_{cells\_uL}}^{BL}$, cells/μL | CD4+ RTE T-lymphocytes in blood (cell concentration) † | $T_{{RTE4}_{cells\_uL}}^{BL}=\frac{T_{RTE4}^{BL}}{BV\left( age \right)*{10}^{3}}$, (**Equation 36**)  where $T_{RTE4}^{BL}$ is the number of RTE CD4+ T-lymphocytes in blood (cells); $BV\left( age \right)$ represents the age-dependent function for the blood volume (mL). |
| $T_{{N4}_{cells\_uL}}^{BL}$, cells/μL | CD4+ naïve T-lymphocytes in blood (cell concentration) † | $T_{{N4}_{cells\_uL}}^{BL}=\frac{T_{N4}^{BL}}{BV\left( age \right)*{10}^{3}}$, (**Equation 37**)  where $T_{N4}^{BL}$ is the number of naïve CD4+ T-lymphocytes in blood (cells); $BV\left( age \right)$ represents the age-dependent function for the blood volume (mL). |
| $T_{{A4}_{cells\_uL}}^{BL}$, cells/μL | CD4+ activated T-lymphocytes in blood (cell concentration) † | $T_{{A4}_{cells\_uL}}^{BL}=\frac{T_{A4}^{BL}}{BV\left( age \right)*{10}^{3}}$, (**Equation 38**)  where $T_{A4}^{BL}$ is the number of activated CD4+ T-lymphocytes in blood (cells); $BV\left( age \right)$ represents the age-dependent function for the blood volume (mL). |
| $T_{{CM4}_{cells\_uL}}^{BL}$, cells/μL | CD4+ central-memory T-lymphocytes in blood (cell concentration) † | $T_{{CM4}_{cells\_uL}}^{BL}=\frac{T_{CM4}^{BL}}{BV\left( age \right)*{10}^{3}}$, (**Equation 39**)  where $T_{CM4}^{BL}$ is the number of central-memory CD4+ T-lymphocytes in blood (cells); $BV\left( age \right)$ represents the age-dependent function for the blood volume (mL). |
| $T_{{EM4}_{cells\_uL}}^{BL}$, cells/μL | CD4+ effector-memory T-lymphocytes in blood (cell concentration) † | $T_{{EM4}_{cells\_uL}}^{BL}=\frac{T_{EM4}^{BL}}{BV\left( age \right)*{10}^{3}}$, (**Equation 40**)  where $T_{EM4}^{BL}$ is the number of effector-memory CD4+ T-lymphocytes in blood (cells); $BV\left( age \right)$ represents the age-dependent function for the blood volume (mL). |
| $T_{{EFF4}_{cells\_uL}}^{BL}$, cells/μL | CD4+ effector T-lymphocytes in blood (cell concentration) † | $T_{{EFF4}_{cells\_uL}}^{BL}=\frac{T_{EFF4}^{BL}}{BV\left( age \right)*{10}^{3}}$, (**Equation 41**)  where $T_{EFF4}^{BL}$ is the number of effector CD4+ T-lymphocytes in blood (cells); $BV\left( age \right)$ represents the age-dependent function for the blood volume (mL). |
| $T_{4_{cells\_uL}}^{BL}$, cells | CD4+ T-lymphocytes in blood (cell concentration) ‡ | $T_{4_{cells\_uL}}^{BL}$ = $T_{N4_{cells\_uL}}^{BL}+T_{{CM4}_{cells\_uL}}^{BL}+$ $T_{EM4_{cells\_uL}}^{BL}+T_{{EFF4}_{cells\_uL}}^{BL}$, (**Equation 42**)  where $T_{N4_{cells\_uL}}^{BL}$, $T_{{CM4}_{cells\_uL}}^{BL}$, $T_{EM4_{cells\_uL}}^{BL}$and $T_{{EFF4}_{cells\_uL}}^{BL}$ are the blood concentration of naïve, central-memory, effector-memory and effector CD4+ T-lymphocytes, respectively (cells/μL). |
| $T_{4}^{LT}$, cells | CD4+ T-lymphocytes in lymphatic tissue (cell count) | $T_{4}^{LT}$ = $T_{N4}^{LT}+T_{CM4}^{LT}+$ $T_{EM4}^{LT}+T_{EFF4}^{LT}$, (**Equation 43**)  where $T_{N4}^{LT}$, $T_{CM4}^{LT}$, $T_{EM4}^{LT}$ and $T_{EFF4}^{LT}$ are the number of naïve, central-memory, effector-memory and effector CD4+ T-lymphocytes in lymphoid tissue, respectively (cells). |
| $T_{4}^{GIT}$, cells | CD4+ T-lymphocytes in gastro-intestinal tract (cell count) | $T_{4}^{GIT}$ = $T_{N4}^{GIT}+T_{CM4}^{GIT}+$ $T_{EM4}^{GIT}+T_{EFF4}^{GIT}$, (**Equation 44**)  where $T_{N4}^{GIT}$, $T_{CM4}^{GIT}$, $T_{EM4}^{GIT}$ and $T_{EFF4}^{GIT}$ are the number of naïve, central-memory, effector-memory and effector CD4+ T-lymphocytes in gastro-intestinal tract, respectively (cells). |
| $T_{4}^{LUNG}$, cells | CD4+ T-lymphocytes in lungs (cell count) | $T_{4}^{LUNG}$ = $T_{N4}^{LUNG}+T_{CM4}^{LUNG}+$ $T_{EM4}^{LUNG}+T_{EFF4}^{LUNG}$, (**Equation 45**)  where $T_{N4}^{LUNG}$, $T_{CM4}^{LUNG}$, $T_{EM4}^{LUNG}$ and $T_{EFF4}^{LUNG}$ are the number of naïve, central-memory, effector-memory and effector CD4+ T-lymphocytes in gastro-intestinal tract, respectively (cells). |
| $T_{{MEM4}_{cells\_uL}}^{BL}$, cells | CD4+ memory T-lymphocytes in blood (cell concentration) ‡ | $T_{MEM4_{cells\_uL}}^{BL}$ = $T_{{CM4}_{cells\_uL}}^{BL}+$ $T_{EM4_{cells\_uL}}^{BL}$, (**Equation 46**)  where $T_{{CM4}_{cells\_uL}}^{BL}$ and $T_{EM4_{cells\_uL}}^{BL}$are the blood concentration of central-memory and effector-memory CD4+ T-lymphocytes, respectively (cells/μL). |
| $T_{MEM4}^{LT}$, cells | CD4+ memory T-lymphocytes in lymphatic tissue (cell count) ‡ | $T_{MEM4}^{LT}$ = $T_{CM4}^{LT}+$ $T_{EM4}^{LT}$, (**Equation 47**)  where $T_{CM4}^{LT}$and $T_{EM4}^{LT}$ are the number of central-memory and effector-memory CD4+ T-lymphocytes in lymphoid tissue, respectively (cells). |
| $T_{MEM4}^{GIT}$, cells | CD4+ memory T-lymphocytes in gastro-intestinal tract (cell count) ‡ | $T_{MEM4}^{GIT}$ = $T_{CM4}^{GIT}+$ $T_{EM4}^{GIT}$, (**Equation 48**)  where $T_{CM4}^{GIT}$and $T_{EM4}^{GIT}$ are the number of central-memory and effector-memory CD4+ T-lymphocytes in gastro-intestinal tract, respectively (cells). |
| $T_{MEM4}^{LUNG}$, cells | CD4+ memory T-lymphocytes in lungs (cell count) ‡ | $T_{MEM4}^{LUNG}$ = $T_{CM4}^{LUNG}+$ $T_{EM4}^{LUNG}$, (**Equation 49**)  where $T_{CM4}^{GIT}$and $T_{EM4}^{GIT}$ are the number of central-memory and effector-memory CD4+ T-lymphocytes in lungs, respectively (cells). |
| $T_{{RTE4}_{perc}}^{LT}$, % | CD4+ RTE T-lymphocytes in lymphatic tissue (cell percentage) † | $T_{{RTE4}_{perc}}^{LT}= \frac{T_{RTE4}^{LT}}{T_{4}^{LT}}*100\%$, (**Equation 50**)  where $T_{RTE4}^{LT}$ is the number of RTE CD4+ T-lymphocytes in lymphoid tissue (cells); $T_{4}^{LT}$is the number of CD4+ T-lymphocytes in lymphoid tissue (cells). |
| $T_{{N4}_{perc}}^{LT}$, % | CD4+ naïve T-lymphocytes in lymphatic tissue (cell percentage) † | $T_{{N4}_{perc}}^{LT}= \frac{T_{N4}^{LT}}{T_{4}^{LT}}*100\%$, (**Equation 51**)  where $T_{N4}^{LT}$ is the number of naïve CD4+ T-lymphocytes in lymphoid tissue (cells); $T_{4}^{LT}$is the number of CD4+ T-lymphocytes in lymphoid tissue (cells). |
| $T_{{A4}_{perc}}^{LT}$, % | CD4+ activated T-lymphocytes in lymphatic tissue (cell percentage) † | $T_{{A4}_{perc}}^{LT}= \frac{T_{A4}^{LT}}{T_{4}^{LT}}*100\%$, (**Equation 52**)  where $T_{A4}^{LT}$ is the number of activated CD4+ T-lymphocytes in lymphoid tissue (cells); $T_{4}^{LT}$is the number of CD4+ T-lymphocytes in lymphoid tissue (cells). |
| $T_{{CM4}_{perc}}^{LT}$, % | CD4+ central-memory T-lymphocytes in lymphatic tissue (cell percentage) † | $T_{{CM4}_{perc}}^{LT}= \frac{T_{CM4}^{LT}}{T_{4}^{LT}}*100\%$, (**Equation 53**)  where $T_{CM4}^{LT}$ is the number of central-memory CD4+ T-lymphocytes in lymphoid tissue (cells); $T_{4}^{LT}$is the number of CD4+ T-lymphocytes in lymphoid tissue (cells). |
| $T_{{EM4}_{perc}}^{LT}$, % | CD4+ effector-memory T-lymphocytes in lymphatic tissue (cell percentage) † | $T_{{EM4}_{perc}}^{LT}= \frac{T_{EM4}^{LT}}{T_{4}^{LT}}*100\%$, (**Equation 54**)  where $T_{EM4}^{LT}$ is the number of effector-memory CD4+ T-lymphocytes in lymphoid tissue (cells); $T_{4}^{LT}$is the number of CD4+ T-lymphocytes in lymphoid tissue (cells). |
| $T_{{EFF4}_{perc}}^{LT}$, % | CD4+ effector T-lymphocytes in lymphatic tissue (cell percentage) † | $T_{{EFF4}_{perc}}^{LT}= \frac{T_{EFF4}^{LT}}{T_{4}^{LT}}*100\%$, (**Equation 55**)  where $T_{EFF4}^{LT}$ is the number of effector CD4+ T-lymphocytes in lymphoid tissue (cells); $T_{4}^{LT}$is the number of CD4+ T-lymphocytes in lymphoid tissue (cells). |
| $T_{{N4}_{perc}}^{GIT}$, % | CD4+ naïve T-lymphocytes in gastro-intestinal tract (cell percentage) † | $T_{{N4}_{perc}}^{GIT}= \frac{T_{N4}^{GIT}}{T_{4}^{GIT}}*100\%$, (**Equation 56**)  where $T_{N4}^{GIT}$ is the number of naïve CD4+ T-lymphocytes in gastro-intestinal tract (cells); $T_{4}^{GIT}$is the number of CD4+ T-lymphocytes in gastro-intestinal tract (cells). |
| $T_{{CM4}_{perc}}^{GIT}$, % | CD4+ central-memory T-lymphocytes in gastro-intestinal tract (cell percentage) † | $T_{{CM4}_{perc}}^{GIT}= \frac{T_{CM4}^{GIT}}{T_{4}^{GIT}}*100\%$, (**Equation 57**)  where $T_{CM4}^{GIT}$ is the number of central-memory CD4+ T-lymphocytes in gastro-intestinal tract (cells); $T_{4}^{GIT}$is the number of CD4+ T-lymphocytes in gastro-intestinal tract (cells). |
| $T_{{EM4}_{perc}}^{GIT}$, % | CD4+ effector-memory T-lymphocytes in gastro-intestinal tract (cell percentage) † | $T_{{EM4}_{perc}}^{GIT}= \frac{T_{EM4}^{GIT}}{T_{4}^{GIT}}*100\%$, (**Equation 58**)  where $T_{EM4}^{GIT}$ is the number of effector-memory CD4+ T-lymphocytes in gastro-intestinal tract (cells); $T_{4}^{GIT}$is the number of CD4+ T-lymphocytes in gastro-intestinal tract (cells). |
| $T_{{EFF4}_{perc}}^{GIT}$, % | CD4+ effector T-lymphocytes in gastro-intestinal tract (cell percentage) † | $T_{{EFF4}_{perc}}^{GIT}= \frac{T_{EFF4}^{GIT}}{T_{4}^{GIT}}*100\%$, (**Equation 59**)  where $T_{EFF4}^{GIT}$ is the number of effector CD4+ T-lymphocytes in gastro-intestinal tract (cells); $T_{4}^{GIT}$is the number of CD4+ T-lymphocytes in gastro-intestinal tract (cells). |
| $T_{{N4}_{perc}}^{LUNG}$, % | CD4+ naïve T-lymphocytes in lungs (cell percentage) † | $T_{{N4}_{perc}}^{LUNG}= \frac{T_{N4}^{LUNG}}{T_{4}^{LUNG}}*100\%$, (**Equation 60**)  where $T_{N4}^{LUNG}$ is the number of naïve CD4+ T-lymphocytes in lungs (cells); $T_{4}^{LUNG}$is the number of CD4+ T-lymphocytes in lungs (cells). |
| $T_{{CM4}_{perc}}^{LUNG}$, % | CD4+ central-memory T-lymphocytes in lungs (cell percentage) † | $T_{{CM4}_{perc}}^{LUNG}= \frac{T_{CM4}^{LUNG}}{T_{4}^{LUNG}}*100\%$, (**Equation 61**)  where $T_{CM4}^{LUNG}$ is the number of central-memory CD4+ T-lymphocytes in lungs (cells); $T_{4}^{LUNG}$is the number of CD4+ T-lymphocytes in lungs (cells). |
| $T_{{EM4}_{perc}}^{LUNG}$, % | CD4+ effector-memory T-lymphocytes in lungs (cell percentage) † | $T_{{EM4}_{perc}}^{LUNG}= \frac{T_{EM4}^{LUNG}}{T_{4}^{LUNG}}*100\%$, (**Equation 62**)  where $T_{EM4}^{LUNG}$ is the number of effector-memory CD4+ T-lymphocytes in lungs (cells); $T_{4}^{LUNG}$is the number of CD4+ T-lymphocytes in lungs (cells). |
| $T_{{EFF4}_{perc}}^{LUNG}$, % | CD4+ effector T-lymphocytes in lungs (cell percentage) † | $T_{{EFF4}_{perc}}^{LUNG}= \frac{T_{EFF4}^{LUNG}}{T_{4}^{LUNG}}*100\%$, (**Equation 63**)  where $T_{EFF4}^{LUNG}$ is the number of effector CD4+ T-lymphocytes in lungs (cells); $T_{4}^{LUNG}$is the number of CD4+ T-lymphocytes in lungs (cells). |

* – Equation 1 was adjusted compared to the previously developed model of thymocytes homeostasis (8)

** – Equations 2-9 were obtained from previously developed model of thymocytes homeostasis (8) without any adaptations

*** – Equations 10-14 were obtained from Sumpter and Holford work (41) with several adjustments: random effects as well as covariate effect were excluded from the model structure

† – Model variables used for calibration

‡ – Model variables used for validation

## Supplementary Table 5

**Supplementary Table 5.** Calibration results for thymus wet weight and blood volume age models

| **Parameter** | **Description** | **Parameter Value (CV, %)*** | **RSE, %** |
| --- | --- | --- | --- |
| $WW_{BL}$, g | Thymus wet weight in infants (0 years-of-age) | 11.09 | 7.67 |
| ${WW}_{max}$, g | Maximum increase in thymus wet weight | 24.45 | 11.48 |
| ${WW}_{{EC50}_{1}}$, years | 50% of the maximum increase in thymus wet weight | 1.09 | 42.29 |
| ${WW}_{{EC50}_{2}}$, years | 50% of the maximum decrease in thymus wet weight | 52.2717 | 14.65 |
| $BV_{BL}$, mL | Intercept of the blood volume dependency on body weight | 357.30836 | 1.97 |
| $k_{BV}$, mL/kg | Slope of the blood volume dependency on body weight | 62.73817 (21.23) | 5.32 |

* – Coefficient of variation (CV%) is calculated based on the value of ω, using the following formula for log-transformed parameters $CV\%= 100*\sqrt{e^{\omega^{2}}-1}$ ($\omega$ – random effect parameter)

## Supplementary Table 6

**Supplementary Table 6.** Age-dependent functions used in the homeostatic CD4+ T-lymphocyte cellular kinetics model

| **Process description** | **Age effect** | **Equation** | **Model structure modification** |
| --- | --- | --- | --- |
| Activated CD4+ T-lymphocyte proliferation | ↑ | $\lambda_{A4}\left( age \right)=\lambda_{A4}*\left( 1+ \frac{\lambda_{{A4}_{max}}*age}{age+ \lambda_{{A4}_{50}}} \right)$  (**Equation 1**)  where $\lambda_{A4}$ represents the proliferation rate of activated CD4+ T-cells in newborns; $\lambda_{{A4}_{max}}$ is a parameter of maximum relative increase in activated CD4+ T-lymphocyte proliferation rate from newborns $\lambda_{A4}$ value as a baseline; $\lambda_{{A4}_{50}}$ represents the age corresponding to a 50% maximum increase in activated CD4+ T-lymphocyte proliferation rate. | Equation 22 in **Supplementary Table 4**  ($\lambda_{A4}$ is replaced by $\lambda_{A4}\left( age \right)$) |
| Naïve CD4+ T-lymphocyte proliferation | ↑ | $\lambda_{N4}\left( age \right)=\lambda_{N4}*\left( 1+ \frac{\lambda_{{N4}_{max}}*{age}^{{Nh}_{\lambda_{N4}}}}{{age}^{{Nh}_{\lambda_{N4}}}+ {\lambda_{{N4}_{50}}}^{{Nh}_{\lambda_{N4}}}} \right)$ (**Equation 2**)  where $\lambda_{N4}$ represents the proliferation rate of naïve CD4+ T-cells in newborns; $\lambda_{{N4}_{max}}$ is a parameter of maximum relative increase in naïve CD4+ T-lymphocyte proliferation rate from newborns $\lambda_{N4}$ value as a baseline; $\lambda_{{N4}_{50}}$ represents the age corresponding to a 50% maximum increase in naïve CD4+ T-lymphocyte proliferation rate; ${Nh}_{\lambda_{N4}}$ is a Hill coefficient. | Equation 18 in **Supplementary Table 4**  ($\lambda_{N4}$ is replaced by $\lambda_{N4}\left( age \right)$) |
| RTE CD4+ T-lymphocyte death | ↓ | $\mu_{RTE4}\left( age \right)=\mu_{RTE4}*\left( 1- \frac{\mu_{{RTE4}_{max}}*{age}^{{Nh}_{\mu_{RTE4}}}}{{age}^{{Nh}_{\mu_{RTE4}}}+ {\mu_{{RTE4}_{50}}}^{{Nh}_{\mu_{RTE4}}}} \right)$  (**Equation 3**)  where $\mu_{RTE4}$ represents the death rate of RTE CD4+ T-cells in newborns; $\mu_{{RTE4}_{max}}$ is a parameter of maximum relative decrease in RTE CD4+ T-lymphocyte death rate from newborns $\mu_{RTE4}$ value as a baseline; $\mu_{{RTE4}_{50}}$ represents the age corresponding to a 50% maximum decrease in RTE CD4+ T-lymphocyte death rate; ${Nh}_{\mu_{RTE4}}$ is a Hill coefficient. | Equations 16, 17 in **Supplementary Table 4**  ($\mu_{RTE4}$ is replaced by $\mu_{RTE4}\left( age \right)$) |
| RTE CD4+ T-lymphocyte transfer from blood to lymphoid tissue | ↓ | $\omega_{{RTE4}_{bl-lt}}\left( age \right)=\omega_{RTE4_{bl-lt}}*\left( 1- \frac{\omega_{{RTE4}_{{bl-lt}_{max}}}*age}{age+\omega_{{RTE4}_{{bl-lt}_{50}}}} \right)$  (**Equation 4**)  where $\omega_{RTE4_{bl-lt}}$ represents the transfer rate of RTE CD4+ T-cells (blood – lymphoid tissue) in newborns; $\omega_{{RTE4}_{{bl-lt}_{max}}}$ is a parameter of maximum relative decrease in RTE CD4+ T-lymphocyte transfer rate from newborns $\omega_{RTE4_{bl-lt}}$ value as a baseline; $\omega_{{RTE4}_{{bl-lt}_{50}}}$ represents the age corresponding to a 50% maximum decrease in RTE CD4+ T-lymphocyte transfer rate. | Equations 16, 17 in **Supplementary Table 4**  ($\omega_{{RTE4}_{bl-lt}}$ is replaced by $\omega_{{RTE4}_{bl-lt}}\left( age \right)$) |
| Central-memory to effector-memory CD4+ T-lymphocyte differentiation | ↓ | $\varphi_{CM4}\left( age \right)=\varphi_{CM4}*\left( 1- \frac{\varphi_{CM4_{max}}*{age}^{{Nh}_{\varphi_{CM4}}}}{{age}^{{Nh}_{\varphi_{CM4}}}+ {\varphi_{CM4_{50}}}^{{Nh}_{\varphi_{CM4}}}} \right)$  (**Equation 5**)  where $\varphi_{CM4}$ represents the differentiation rate of CM CD4+ T-cells in newborns; $\varphi_{CM4_{max}}$ is a parameter of maximum relative decrease in CM CD4+ T-lymphocyte differentiation rate from newborns $\varphi_{CM4_{max}}$ value as a baseline; $\varphi_{CM4_{50}}$ represents the age corresponding to a 50% maximum decrease in CM CD4+ T-lymphocyte differentiation rate; ${Nh}_{\varphi_{CM4}}$ is a Hill coefficient. | Equations 24, 28 in **Supplementary Table 4**  ($\varphi_{CM4}$ is replaced by $\varphi_{CM4}\left( age \right)$) |
| Effector-memory to effector-memory CD4+ T-lymphocyte differentiation | ↓ | $\varphi_{EM4}\left( age \right)=\varphi_{EM4}*\left( 1- \frac{\varphi_{EM4_{max}}*age}{age+ age_{50}} \right)$  (**Equation 6**)  where $\varphi_{EM4}$ represents the differentiation rate of EM CD4+ T-cells in newborns; $\varphi_{EM4_{max}}$ is a parameter of maximum relative decrease in EM CD4+ T-lymphocyte differentiation rate from newborns $\varphi_{EM4_{max}}$ value as a baseline; $\varphi_{EM4_{50}}$ represents the age corresponding to a 50% maximum decrease in EM CD4+ T-lymphocyte differentiation rate. | Equations 28, 32 in **Supplementary Table 4**  ($\varphi_{EM4}$ is replaced by $\varphi_{EM4}\left( age \right)$) |
| Effector-memory CD4+ T-lymphocyte transfer from lungs to blood | ↑ | $\omega_{{EM4}_{lung-bl}}\left( age \right)=\omega_{E{M4}_{lung-bl}}*\left( 1+ \frac{\omega_{{EM4}_{{lung-bl}_{max}}}*age}{age+age_{50}} \right)$  (**Equation 7**)  where $\omega_{E{M4}_{lung-bl}}$ represents the transfer rate (lung - blood) of EM CD4+ T-cells in newborns;$\omega_{{EM4}_{{lung-bl}_{max}}}$ is a parameter of maximum relative increase in EM CD4+ T-lymphocyte transfer rate from newborns $\omega_{E{M4}_{lung-bl}}$ value as a baseline; $age_{50}$ represents the age corresponding to a 50% maximum increase in EM CD4+ T-lymphocyte transfer rate. | Equations 29, 31 in **Supplementary Table 4**  ($\omega_{{EM4}_{lung-bl}}$ is replaced by $\omega_{{EM4}_{lung-bl}}\left( age \right)$) |
| Effector CD4+ T-lymphocyte transfer from blood to gastro-intestinal tract | ↑ | $\omega_{{EFF4}_{bl-git}}\left( age \right)=\omega_{{EFF4}_{bl-git}}*\left( 1+ \frac{\omega_{{EFF4}_{{bl-tis}_{max}}}*age}{age+age_{50}} \right)$  (**Equation 8**)  where $\omega_{{EFF4}_{bl-git}}$ represents the transfer rate (blood – gastro-intestinal tract) of effector CD4+ T-cells in newborns;$\omega_{{EFF4}_{{bl-tis}_{max}}}$ is a parameter of maximum relative increase in effector CD4+ T-lymphocyte transfer rate from newborns $\omega_{{EFF4}_{bl-git}}$ value as a baseline; $age_{50}$ represents the age corresponding to a 50% maximum increase in effector CD4+ T-lymphocyte transfer rate. | Equations 33, 34 in **Supplementary Table 4**  ($\omega_{{EFF4}_{bl-git}}$ is replaced by $\omega_{{EFF4}_{bl-git}}\left( age \right)$) |
| Effector CD4+ T-lymphocyte transfer from blood to lungs | ↑ | $\omega_{{EFF4}_{bl-lung}}\left( age \right)=\omega_{{EFF4}_{bl-lung}}*\left( 1+ \frac{\omega_{{EFF4}_{{bl-tis}_{max}}}*age}{age+age_{50}} \right)$  (**Equation 9**)  where $\omega_{{EFF4}_{bl-lung}}$ represents the transfer rate (blood – lungs) of effector CD4+ T-cells in newborns;$\omega_{{EFF4}_{{bl-tis}_{max}}}$ is a parameter of maximum relative increase in effector CD4+ T-lymphocyte transfer rate from newborns $\omega_{{EFF4}_{bl-lung}}$ value as a baseline; $age_{50}$ represents the age corresponding to a 50% maximum increase in effector CD4+ T-lymphocyte transfer rate. | Equations 33, 35 in **Supplementary Table 4**  ($\omega_{{EFF4}_{bl-lung}}$ is replaced by $\omega_{{EFF4}_{bl-lung}}\left( age \right)$) |
| Effector CD4+ T-lymphocyte transfer from blood to other peripheral tissues | ↑ | $\omega_{{EFF4}_{bl-tis}}\left( age \right)=\omega_{{EFF4}_{bl-tis}}*\left( 1+ \frac{\omega_{{EFF4}_{{bl-tis}_{max}}}*age}{age+age_{50}} \right)$  (**Equation 10**)  where $\omega_{{EFF4}_{bl-tis}}$ represents the transfer rate (blood – peripheral tissues) of effector CD4+ T-cells in newborns;$\omega_{{EFF4}_{{bl-tis}_{max}}}$ is a parameter of maximum relative increase in effector CD4+ T-lymphocyte transfer rate from newborns $\omega_{{EFF4}_{bl-tis}}$ value as a baseline; $age_{50}$ represents the age corresponding to a 50% maximum increase in effector CD4+ T-lymphocyte transfer rate. | Equation 33 in Supplementary Table 4  ($\omega_{{EFF4}_{bl-tis}}$ is replaced by $\omega_{{EFF4}_{bl-tis}}\left( age \right)$) |
| Activated CD4+ T-lymphocyte transfer from lymphoid tissue to blood | ↓ | $\omega_{{A4}_{lt-bl}}\left( age \right)=\omega_{{A4}_{lt-bl}}*\left( 1- \frac{\omega_{{A4}_{{lt-bl}_{max}}}*age}{age+\omega_{{A4}_{{lt-bl}_{50}}}} \right)$ (**Equation 11**)  where $\omega_{{A4}_{lt-bl}}$ represents the transfer rate of activated CD4+ T-cells (lymphoid tissue – blood) in newborns; $\omega_{{A4}_{{lt-bl}_{max}}}$ is a parameter of maximum relative decrease in activated CD4+ T-lymphocyte transfer rate from newborns $\omega_{{A4}_{lt-bl}}$ value as a baseline; $\omega_{{A4}_{{lt-bl}_{50}}}$ represents the age corresponding to a 50% maximum decrease in activated CD4+ T-lymphocyte transfer rate. | Equations 22, 23 in **Supplementary Table 4**  ($\omega_{{A4}_{lt-bl}}$ is replaced by $\omega_{{A4}_{lt-bl}}\left( age \right)$) |
| Central-memory CD4+ T-lymphocyte transfer from lymphoid tissue to blood | ↓ | $\omega_{{CM4}_{lt-bl}}\left( age \right)=\omega_{{CM4}_{lt-bl}}*\left( 1- \frac{\omega_{{CM4}_{{lt-bl}_{max}}}*age}{age+\omega_{{CM4}_{{lt-bl}_{50}}}} \right)$  (**Equation 12**)  where $\omega_{{CM4}_{lt-bl}}$ represents the transfer rate of CM CD4+ T-cells (lymphoid tissue – blood) in newborns; $\omega_{{CM4}_{{lt-bl}_{max}}}$ is a parameter of maximum relative decrease in CM CD4+ T-lymphocyte transfer rate from newborns $\omega_{{CM4}_{lt-bl}}$ value as a baseline; $\omega_{{CM4}_{{lt-bl}_{50}}}$ represents the age corresponding to a 50% maximum decrease in CM CD4+ T-lymphocyte transfer rate. | Equation 24, 25 in **Supplementary Table 4**  ($\omega_{{CM4}_{lt-bl}}$ is replaced by $\omega_{{CM4}_{lt-bl}}\left( age \right)$) |
| Effector CD4+ T-lymphocyte transfer from lymphoid tissue to blood | ↓ | $\omega_{{EFF4}_{lt-bl}}\left( age \right)=\omega_{{EFF4}_{lt-bl}}*\left( 1- \frac{\omega_{{EFF4}_{{lt-bl}_{max}}}*age}{age+\omega_{{EFF4}_{{lt-bl}_{50}}}} \right)$  (**Equation 13**)  where $\omega_{{EFF4}_{lt-bl}}$ represents the transfer rate of effector CD4+ T-cells (lymphoid tissue – blood) in newborns; $\omega_{{EFF4}_{{lt-bl}_{max}}}$ is a parameter of maximum relative decrease in effector CD4+ T-lymphocyte transfer rate from newborns $\omega_{{EFF4}_{lt-bl}}$ value as a baseline; $\omega_{{EFF4}_{{lt-bl}_{50}}}$ represents the age corresponding to a 50% maximum decrease in effector CD4+ T-lymphocyte transfer rate. | Equation 28, 29 in **Supplementary Table 4**  ($\omega_{{EFF4}_{lt-bl}}$ is replaced by $\omega_{{EFF4}_{lt-bl}}\left( age \right)$) |

## Supplementary Table 7

**Supplementary Table 7.** Homeostatic CD4+ T-lymphocyte cellular kinetic model (with age effect incorporation) calibration results

| **Parameter** | **Description** | **Parameter Value** | **RSE, %** |
| --- | --- | --- | --- |
| $\lambda_{{A4}_{max}}$, - | Maximum relative increase in the activated CD4+ T-lymphocyte proliferation rate from newborns $\lambda_{A4}$ value as a baseline | 0.023 | 0.70 |
| $\lambda_{{A4}_{50}}$, years | Age corresponding to a 50% maximum increase in the activated CD4+ T-lymphocyte proliferation rate | 4.06 | 5.33 |
| $\lambda_{{N4}_{max}}$, - | Maximum relative increase in the naïve CD4+ T-lymphocyte proliferation rate from newborns $\lambda_{N4}$ value as a baseline | 0.775 | 7.20 |
| $\lambda_{{N4}_{50}}$, years | Age corresponding to a 50% maximum increase in the naïve CD4+ T-lymphocyte proliferation rate | 56.67 | 1.30 |
| ${Nh}_{\lambda_{N4}}$, - | Hill coefficient in the naïve CD4+ T-lymphocyte proliferation rate age-dependent function | 10 | Fixed |
| $\mu_{{RTE4}_{max}}$, - | Maximum relative decrease in the RTE CD4+ T-lymphocyte death rate from newborns $\mu_{RTE4}$ value as a baseline | 0.86 | 2.31 |
| $\mu_{{RTE4}_{50}}$, years | Age corresponding to a 50% maximum increase in the RTE CD4+ T-lymphocyte death rate | 24.02 | 2.62 |
| ${Nh}_{\mu_{RTE4}}$, - | Hill coefficient in the RTE CD4+ T-lymphocyte death rate age-dependent function | 4.49 | 13.00 |
| $\omega_{{RTE4}_{{bl-lt}_{max}}}$, - | Maximum relative decrease in the RTE CD4+ T-lymphocyte blood – lymphoid tissue transfer rate from newborns $\omega_{{RTE4}_{bl-lt}}$ value as a baseline | 1 | Fixed |
| $\omega_{{RTE4}_{{bl-lt}_{50}}}$, years | Age corresponding to a 50% maximum decrease in the RTE CD4+ T-lymphocyte blood – lymphoid tissue transfer rate | 21.44 | 3.92 |
| $\varphi_{CM4_{max}}$, - | Maximum relative decrease in the central-memory CD4+ T-lymphocyte differentiation rate from newborns $\varphi_{CM4}$ value as a baseline | 1 | Fixed |
| $\varphi_{CM4_{50}}$, years | Age corresponding to a 50% maximum increase in the central-memory CD4+ T-lymphocyte differentiation rate | 62.55 | 3.72 |
| ${Nh}_{\varphi_{CM4}}$, - | Hill coefficient in the central-memory CD4+ T-lymphocyte differentiation rate age-dependent function | 3 | Fixed |
| $\varphi_{EM4_{max}}$, - | Maximum relative decrease in the effector-memory CD4+ T-lymphocyte differentiation rate from newborns $\varphi_{EM4}$ value as a baseline | 1 | Fixed |
| $age_{50}$, years | Age corresponding to a 50% maximum increase in the: 1) effector-memory CD4+ T-lymphocyte differentiation rate; 2) effector-memory CD4+ T-lymphocyte transfer rate from lungs to blood; 3) effector CD4+ T-lymphocyte transfer rates from blood to gastro-intestinal tract, lungs and other peripheral tissues | 4.40 | 34.55 |
| $\omega_{{EM4}_{{lung-bl}_{max}}}$, - | Maximum relative increase in the effector-memory CD4+ T-lymphocyte lungs – blood transfer rate from newborns $\omega_{{EM4}_{lung-bl}}$ value as a baseline | 9.50 | 15.06 |
| $\omega_{{EFF4}_{{bl-tis}_{max}}}$, - | Maximum relative increase in the effector CD4+ T-lymphocyte blood transfer rates from blood to gastro-intestinal tract, lungs and other peripheral tissues from newborns $\omega_{{EFF4}_{bl-git}}= \omega_{{EFF4}_{bl-lung}}=\omega_{{EFF4}_{bl-tis}}$ value as a baseline | 1.44 | 16.39 |
| $\omega_{{A4}_{{lt-bl}_{max}}}$, - | Maximum relative decrease in the activated CD4+ T-lymphocyte lymphoid tissue – blood transfer rate from newborns $\omega_{{A4}_{lt-bl}}$ value as a baseline | 1 | Fixed |
| $\omega_{{A4}_{{lt-bl}_{50}}}$, years | Age corresponding to a 50% maximum increase in the activated CD4+ T-lymphocyte lymphoid tissue – blood transfer rate | 3.87 | 5.15 |
| $\omega_{{CM4}_{{lt-bl}_{max}}}$, - | Maximum relative decrease in the central-memory CD4+ T-lymphocyte lymphoid tissue – blood transfer rate from newborns $\omega_{{CM4}_{lt-bl}}$ value as a baseline | 1 | Fixed |
| $\omega_{{CM4}_{{lt-bl}_{50}}}$, years | Age corresponding to a 50% maximum increase in the central-memory CD4+ T-lymphocyte lymphoid tissue – blood transfer rate | 116.14 | 14.62 |
| $\omega_{{EFF4}_{{lt-bl}_{max}}}$, - | Maximum relative decrease in the effector CD4+ T-lymphocyte lymphoid tissue – blood transfer rate from newborns $\omega_{{EFF4}_{lt-bl}}$ value as a baseline | 1 | Fixed |
| $\omega_{{EFF4}_{{lt-bl}_{50}}}$, years | Age corresponding to a 50% maximum increase in the effector CD4+ T-lymphocyte lymphoid tissue – blood transfer rate | 9.38 | 6.76 |

## Supplementary Table 8

**Supplementary Table 8.** Age-dependent CD4+ T-lymphocyte homeostasis model comparison (backward elimination)

| **Model #** | **Description** | **Identifiability (not identified parameters)** | **-2LL** | **AIC** |
| --- | --- | --- | --- | --- |
| 1 | Base model | Yes | 4832.35 | 4862.35 |
| 2 | Model without $\omega_{{CM4}_{lt-bl}}\left( age \right)$ (**Equation 12 in Supplementary Table 6**) | Yes | 4890.56 | 4918.56 |
| 3 | Model without $\omega_{{EFF4}_{bl-git}}\left( age \right)$, $\omega_{{EFF4}_{bl-lung}}\left( age \right)$ and $\omega_{{EFF4}_{bl-tis}}\left( age \right)$ (**Equations 8 – 10 in Supplementary Table 6**) | Yes | 4894.57 | 4922.57 |
| 4 | Model without $\varphi_{EM4}\left( age \right)$ (**Equation 6 in Supplementary Table 6**) | Yes | 4924.52 | 4954.52 |
| 5 | Model without $\varphi_{CM4}\left( age \right)$ (**Equation 5 in Supplementary Table 6**) | No ($\omega_{{CM4}_{{lt-bl}_{50}}}$) | 4990.02 | 5018.02 |
| 6 | Model without $\lambda_{N4}\left( age \right)$ (**Equation 1 in Supplementary Table 6**) | Yes | 5011.21 | 5037.21 |
| 7 | Model without $\omega_{{EFF4}_{lt-bl}}\left( age \right)$ (**Equations 13 in Supplementary Table 6**) | No ($\omega_{{EFF4}_{{bl-tis}_{max}}}$) | 5038.54 | 5066.54 |
| 8 | Model without $\omega_{{EM4}_{lung-bl}}\left( age \right)$ (**Equation 7 in Supplementary Table 6**) | Yes | 5044.63 | 5072.63 |
| 9 | Model without $\omega_{{A4}_{lt-bl}}\left( age \right)$ (**Equation 11 in Supplementary Table 6**) | No ($\omega_{{EFF4}_{{bl-tis}_{max}}}$) | 5268.00 | 5296.00 |
| 10 | Model without $\mu_{RTE4}\left( age \right)$ (**Equation 3 in Supplementary Table 6**) | No ($\omega_{{EM4}_{{lung-bl}_{max}}},$ $\omega_{{EFF4}_{{bl-tis}_{max}}}$, $\omega_{{CM4}_{{lt-bl}_{50}}}$) | 10035.27 | 10059.27 |
| 11 | Model without $\omega_{RTE4_{bl-lt}}\left( age \right)$ (**Equation 4 in Supplementary Table 6**) | Yes | 11723.23 | 11751.23 |
| 12 | Model without $\lambda_{A4}\left( age \right)$ (**Equation 2 in Supplementary Table 6**) | No ($\mu_{{RTE4}_{max}}$, ${age}_{50}$) | 20291.87 | 20317.87 |

## Supplementary Table 9

**Supplementary Table 9.** Model equations used in homeostatic CD4+ T-lymphocyte cellular kinetics model with incorporated age effect and cellular homeostatic feedback

| **Process description** | **Age effect** | **Cell count effect** | **Equation** | **Model structure modification** |
| --- | --- | --- | --- | --- |
| Naïve CD4+ T-lymphocyte proliferation | ↑ | ↓ | $\lambda_{N4}\left( T_{{RTE4}_{cells\_uL}}^{BL} \right)=\lambda_{N4_{BASE}}*\left( 1- \frac{\lambda_{{N4}_{max}}^{RTE4}*{T_{{RTE4}_{cells\_uL}}^{BL}}^{{Nh}_{\lambda_{N4}}}}{{T_{{RTE4}_{cells\_uL}}^{BL}}^{{Nh}_{\lambda_{N4}}}+ {\lambda_{{N4}_{50}}^{RTE4}}^{{Nh}_{\lambda_{N4}}}} \right)$  $\lambda_{N4_{BASE}}=\frac{\lambda_{N4}}{\left( 1- \frac{\lambda_{{N4}_{max}}^{RTE4}*{T_{RTE4_{{cells_{uL}}_{\left( age=0 \right)}}}^{BL}}^{{Nh}_{\lambda_{N4}}}}{{T_{RTE4_{{cells_{uL}}_{\left( age=0 \right)}}}^{BL}}^{{Nh}_{\lambda_{N4}}} + {\lambda_{{N4}_{50}}^{RTE4}}^{{Nh}_{\lambda_{N4}}}} \right)}$  $T_{{RTE4}_{{cells\_uL}_{(age=0)}}}^{BL}$ = 1494.1 cells/μL  (**Equation 1**)  where $\lambda_{N4}$ represents proliferation rate of naïve CD4+ T-cells in newborns; $T_{{RTE4}_{cells\_uL}}^{BL}$ corresponds to blood concentration of RTE CD4+ T-cells (cells/μL); $\lambda_{{N4}_{max}}^{RTE4}$ is a parameter of maximum relative decrease in naïve CD4+ T-lymphocyte proliferation rate from the maximum value of proliferation rate ($\lambda_{N4_{BASE}}$) value as a baseline; $\lambda_{{N4}_{50}}^{RTE4}$ represents the RTE CD4+ T-cell concentration in blood corresponding to a 50% maximum decrease in naïve CD4+ T-lymphocyte proliferation rate; ${Nh}_{\lambda_{N4}}$ is a Hill coefficient. | Equation 1 in **Supplementary Table 6** |
| RTE CD4+ T-lymphocyte death | ↓ | ↑ | $\mu_{RTE4}\left( T_{{RTE4}_{cells\_uL}}^{BL} \right)=\mu_{RTE4_{BASE}}*\left( 1+ \frac{\mu_{{RTE4}_{max}}^{RTE4}*{T_{{RTE4}_{cells\_uL}}^{BL}}^{{Nh}_{\mu_{RTE4}}}}{{T_{{RTE4}_{cells\_uL}}^{BL}}^{{Nh}_{\mu_{RTE4}}} + {\mu_{{RTE4}_{50}}^{RTE4}}^{{Nh}_{\mu_{RTE4}}}} \right)$  $\mu_{RTE4_{BASE}}=\frac{\mu_{RTE4}}{\left( 1 + \frac{\mu_{{RTE4}_{max}}^{RTE4}*{T_{{RTE4}_{{cells\_uL}_{(age=0)}}}^{BL}}^{{Nh}_{\mu_{RTE4}}}}{{T_{{RTE4}_{{cells\_uL}_{(age=0)}}}^{BL}}^{{Nh}_{\mu_{RTE4}}} + {\mu_{{RTE4}_{50}}^{RTE4}}^{{Nh}_{\mu_{RTE4}}}} \right)}$  $T_{{RTE4}_{{cells\_uL}_{(age=0)}}}^{BL}$ = 1494.1 cells/μL  (**Equation 2**)  where $\mu_{RTE4}$ represents death rate of RTE CD4+ T-cells in newborns; $T_{{RTE4}_{cells\_uL}}^{BL}$ corresponds to blood concentration of RTE CD4+ T-cells (cells/μL); $\mu_{{RTE4}_{max}}^{RTE4}$ is a parameter of maximum relative increase in RTE CD4+ T-lymphocyte death rate from the maximum value of death rate ($\mu_{RTE4_{BASE}}$) value as a baseline; $\mu_{{RTE4}_{50}}^{RTE4}$represents the RTE CD4+ T-cell concentration in blood corresponding to a 50% maximum increase in RTE CD4+ T-lymphocyte death rate; ${Nh}_{\mu_{RTE4}}$ is a Hill coefficient. | Equation 3 in **Supplementary Table 6** |

## Supplementary Table 10

**Supplementary Table 10.** Homeostatic CD4+ T-lymphocyte cellular kinetics model with incorporated age effect and cellular homeostatic feedback calibration results

| **Parameter** | **Description** | **Parameter Value** | **RSE, %** |
| --- | --- | --- | --- |
| $\lambda_{{A4}_{max}}$, - | Maximum relative increase in the activated CD4+ T-lymphocyte proliferation rate from newborns $\lambda_{A4}$ value as a baseline | 0.023 | 0.70 |
| $\lambda_{{A4}_{50}}$, years | Age corresponding to a 50% maximum increase in activated CD4+ T-lymphocyte proliferation rate | 4.00 | 5.25 |
| $\lambda_{{N4}_{max}}^{RTE4}$, - | Maximum relative decrease in the naïve CD4+ T-lymphocyte proliferation rate from the maximum value of proliferation rate ($\lambda_{N4_{BASE}}$) value as a baseline | 0.43 | 2.66 |
| $\lambda_{{N4}_{50}}^{RTE4}$, cells/μL | RTE CD4+ T-cell concentration in blood corresponding to a 50% maximum decrease in naïve CD4+ T-lymphocyte proliferation rate | 201.54 | 2.95 |
| ${Nh}_{\lambda_{N4}}$, - | Hill coefficient in the naïve CD4+ T-lymphocyte proliferation rate cell count dependent function | 10 | Fixed |
| $\mu_{{RTE4}_{max}}^{RTE4}$, - | Maximum relative increase in the RTE CD4+ T-lymphocyte death rate from the maximum value of death rate ($\mu_{RTE4_{BASE}}$) value as a baseline | 5.06 | 8.38 |
| $\mu_{{RTE4}_{50}}^{RTE4}$, cells/μL | RTE CD4+ T-cell concentration in the blood corresponding to a 50% maximum increase in RTE CD4+ T-lymphocyte death rate | 363.38 | 2.45 |
| ${Nh}_{\mu_{RTE4}}$, - | Hill coefficient in the RTE CD4+ T-lymphocyte death rate cell concentration dependent function | 10 | Fixed |
| $\omega_{{RTE4}_{{bl-lt}_{max}}}$, - | Maximum relative decrease in the RTE CD4+ T-lymphocyte blood – lymphoid tissue transfer rate from newborns $\omega_{{RTE4}_{bl-lt}}$ value as a baseline | 1 | Fixed |
| $\omega_{{RTE4}_{{bl-lt}_{50}}}$, years | Age corresponding to a 50% maximum decrease in RTE CD4+ T-lymphocyte blood – lymphoid tissue transfer rate | 23.44 | 4.00 |
| $\varphi_{CM4_{max}}$, - | Maximum relative decrease in the central-memory CD4+ T-lymphocyte differentiation rate from newborns $\varphi_{CM4}$ value as a baseline | 1 | Fixed |
| $\varphi_{CM4_{50}}$, years | Age corresponding to a 50% maximum increase in central-memory CD4+ T-lymphocyte differentiation rate | 62.88 | 3.71 |
| ${Nh}_{\varphi_{CM4}}$, - | Hill coefficient in the central-memory CD4+ T-lymphocyte differentiation rate age-dependent function | 3 | Fixed |
| $\varphi_{EM4_{max}}$, - | Maximum relative decrease in the effector-memory CD4+ T-lymphocyte differentiation rate from newborns $\varphi_{EM4}$ value as a baseline | 1 | Fixed |
| $age_{50}$, years | Age corresponding to a 50% maximum increase in the: 1) effector-memory CD4+ T-lymphocyte differentiation rate; 2) effector-memory CD4+ T-lymphocyte transfer rate from lungs to blood; 3) effector CD4+ T-lymphocyte transfer rates from blood to gastro-intestinal tract, lungs and other peripheral tissues | 4.55 | 34.15 |
| $\omega_{{EM4}_{{lung-bl}_{max}}}$, - | Maximum relative increase in the effector-memory CD4+ T-lymphocyte lungs – blood transfer rate from newborns $\omega_{{EM4}_{lung-bl}}$ value as a baseline | 9.81 | 14.90 |
| $\omega_{{EFF4}_{{bl-tis}_{max}}}$, - | Maximum relative increase in the effector CD4+ T-lymphocyte blood transfer rates from blood to gastro-intestinal tract, lungs and other peripheral tissues from newborns $\omega_{{EFF4}_{bl-git}}= \omega_{{EFF4}_{bl-lung}}=\omega_{{EFF4}_{bl-tis}}$ value as a baseline | 1.47 | 16.25 |
| $\omega_{{A4}_{{lt-bl}_{max}}}$, - | Maximum relative decrease in the activated CD4+ T-lymphocyte lymphoid tissue – blood transfer rate from newborns $\omega_{{A4}_{lt-bl}}$ value as a baseline | 1 | Fixed |
| $\omega_{{A4}_{{lt-bl}_{50}}}$, years | Age corresponding to a 50% maximum increase in activated CD4+ T-lymphocyte lymphoid tissue – blood transfer rate | 3.85 | 5.12 |
| $\omega_{{CM4}_{{lt-bl}_{max}}}$, - | Maximum relative decrease in the central-memory CD4+ T-lymphocyte lymphoid tissue – blood transfer rate from newborns $\omega_{{CM4}_{lt-bl}}$ value as a baseline | 1 | Fixed |
| $\omega_{{CM4}_{{lt-bl}_{50}}}$, years | Age corresponding to a 50% maximum increase in central-memory CD4+ T-lymphocyte lymphoid tissue – blood transfer rate | 126.48 | 15.38 |
| $\omega_{{EFF4}_{{lt-bl}_{max}}}$, - | Maximum relative decrease in the effector CD4+ T-lymphocyte lymphoid tissue – blood transfer rate from newborns $\omega_{{EFF4}_{lt-bl}}$ value as a baseline | 1 | Fixed |
| $\omega_{{EFF4}_{{lt-bl}_{50}}}$, years | Age corresponding to a 50% maximum increase in effector CD4+ T-lymphocyte lymphoid tissue – blood transfer rate | 9.33 | 6.71 |

## Supplementary Table 11

**Supplementary Table 11.** Age-dependent functions used for each step in the homeostatic model development

| **Homeostatic CD4+ T-lymphocyte cellular kinetics model** | | |
| --- | --- | --- |
| **-** | **with age effect** | **with age effect and cell count feedback** |
| ***Thymus involution*** | | |
| $T_{cort}^{max}\left( age \right)= \frac{T_{0}CM\left( age \right)}{CM\left( age \right)+1}*\frac{WW\left( age \right)*TES\left( age \right)}{WW\left( 0 \right)*TES\left( 0 \right)}$ (for details see **Supplementary Table 4**, Equation 4)  $T_{med}^{max}\left( age \right)= \frac{T_{0}}{CM\left( age \right)+1}*\frac{WW\left( age \right)*TES\left( age \right)}{WW\left( 0 \right)*TES\left( 0 \right)}$ (for details see **Supplementary Table 4**, Equation 5) | | |
| ***Blood volume*** | | |
| $BV\left( age \right)=BV_{BL}+k_{BV}*WT(age)$ (for details see **Supplementary Table 4**, Equation 15) | | |
|  | ***Clonal expansion*** | |
|  | $\lambda_{A4}\left( age \right)=\lambda_{A4}*\left( 1+ \frac{\lambda_{{A4}_{max}}*age}{age+ \lambda_{{A4}_{50}}} \right)$  (for details see **Supplementary Table 6**, Equation 1) | |
|  | ***Memory subsets differentiation*** | |
|  | $\varphi_{CM4}\left( age \right)=\varphi_{CM4}*\left( 1- \frac{\varphi_{CM4_{max}}*{age}^{{Nh}_{\varphi_{CM4}}}}{{age}^{{Nh}_{\varphi_{CM4}}}+ {\varphi_{CM4_{50}}}^{{Nh}_{\varphi_{CM4}}}} \right)$  (for details see **Supplementary Table 6**, Equation 5)  $\varphi_{EM4}\left( age \right)=\varphi_{EM4}*\left( 1- \frac{\varphi_{EM4_{max}}*age}{age+ age_{50}} \right)$  (for details see **Supplementary Table 6**, Equation 6) | |
|  | ***Transitions from/to lymphoid tissue*** | |
|  | $\omega_{{RTE4}_{bl-lt}}\left( age \right)=\omega_{RTE4_{bl-lt}}*\left( 1- \frac{\omega_{{RTE4}_{{bl-lt}_{max}}}*age}{age+\omega_{{RTE4}_{{bl-lt}_{50}}}} \right)$  (for details see **Supplementary Table 6**, Equation 4)  $\omega_{{A4}_{lt-bl}}\left( age \right)=\omega_{{A4}_{lt-bl}}*\left( 1- \frac{\omega_{{A4}_{{lt-bl}_{max}}}*age}{age+\omega_{{A4}_{{lt-bl}_{50}}}} \right)$  (for details see **Supplementary Table 6**, Equation 11)  $\omega_{{CM4}_{lt-bl}}\left( age \right)=\omega_{{CM4}_{lt-bl}}*\left( 1- \frac{\omega_{{CM4}_{{lt-bl}_{max}}}*age}{age+\omega_{{CM4}_{{lt-bl}_{50}}}} \right)$  (for details see **Supplementary Table 6**, Equation 12)  $\omega_{{EFF4}_{lt-bl}}\left( age \right)=\omega_{{EFF4}_{lt-bl}}*\left( 1- \frac{\omega_{{EFF4}_{{lt-bl}_{max}}}*age}{age+\omega_{{EFF4}_{{lt-bl}_{50}}}} \right)$  (for details see **Supplementary Table 6**, Equation 13) | |
|  | ***Transitions from/to peripheral organs*** | |
|  | $\omega_{{EM4}_{lung-bl}}\left( age \right)=\omega_{E{M4}_{lung-bl}}*\left( 1+ \frac{\omega_{{EM4}_{{lung-bl}_{max}}}*age}{age+age_{50}} \right)$  (for details see **Supplementary Table 6**, Equation 7)  $\omega_{{EFF4}_{bl-git}}\left( age \right)=\omega_{{EFF4}_{bl-git}}*\left( 1+ \frac{\omega_{{EFF4}_{{bl-tis}_{max}}}*age}{age+age_{50}} \right)$  (for details see **Supplementary Table 6**, Equation 8)  $\omega_{{EFF4}_{bl-lung}}\left( age \right)=\omega_{{EFF4}_{bl-lung}}*\left( 1+ \frac{\omega_{{EFF4}_{{bl-tis}_{max}}}*age}{age+age_{50}} \right)$  (for details see **Supplementary Table 6**, Equation 9)  $\omega_{{EFF4}_{bl-tis}}\left( age \right)=\omega_{{EFF4}_{bl-tis}}*\left( 1+ \frac{\omega_{{EFF4}_{{bl-tis}_{max}}}*age}{age+age_{50}} \right)$  (for details see **Supplementary Table 6**, Equation 10) | |
|  | ***Naïve CD4+ T-cell proliferation*** | ***Compensatory changes in naïve CD4+ T-cell proliferation*** |
|  | $\lambda_{N4}\left( age \right)=\lambda_{N4}*\left( 1+ \frac{\lambda_{{N4}_{max}}*{age}^{{Nh}_{\lambda_{N4}}}}{{age}^{{Nh}_{\lambda_{N4}}}+ {\lambda_{{N4}_{50}}}^{{Nh}_{\lambda_{N4}}}} \right)$  (for details see **Supplementary Table 6**, Equation 2) | $\lambda_{N4}\left( T_{{RTE4}_{cells\_uL}}^{BL} \right)=\lambda_{N4_{BASE}}*\left( 1- \frac{\lambda_{{N4}_{max}}^{RTE4}*{T_{{RTE4}_{cells\_uL}}^{BL}}^{{Nh}_{\lambda_{N4}}}}{{T_{{RTE4}_{cells\_uL}}^{BL}}^{{Nh}_{\lambda_{N4}}}+ {\lambda_{{N4}_{50}}^{RTE4}}^{{Nh}_{\lambda_{N4}}}} \right)$  (for details see **Supplementary Table 9**, Equation 1) |
|  | ***RTE CD4+ T-cell survival*** | ***Compensatory changes in RTE CD4+ T-cell survival*** |
|  | $\mu_{RTE4}\left( age \right)=\mu_{RTE4}*\left( 1- \frac{\mu_{{RTE4}_{max}}*{age}^{{Nh}_{\mu_{RTE4}}}}{{age}^{{Nh}_{\mu_{RTE4}}}+ {\mu_{{RTE4}_{50}}}^{{Nh}_{\mu_{RTE4}}}} \right)$  (for details see **Supplementary Table 6**, Equation 3) | $\mu_{RTE4}\left( T_{{RTE4}_{cells\_uL}}^{BL} \right)=\mu_{RTE4_{BASE}}*\left( 1+ \frac{\mu_{{RTE4}_{max}}^{RTE4}*{T_{{RTE4}_{cells\_uL}}^{BL}}^{{Nh}_{\mu_{RTE4}}}}{{T_{{RTE4}_{cells\_uL}}^{BL}}^{{Nh}_{\mu_{RTE4}}} + {\mu_{{RTE4}_{50}}^{RTE4}}^{{Nh}_{\mu_{RTE4}}}} \right)$  (for details see **Supplementary Table 9**, Equation 2) |

# Supplementary Figures

## Supplementary Figure 1


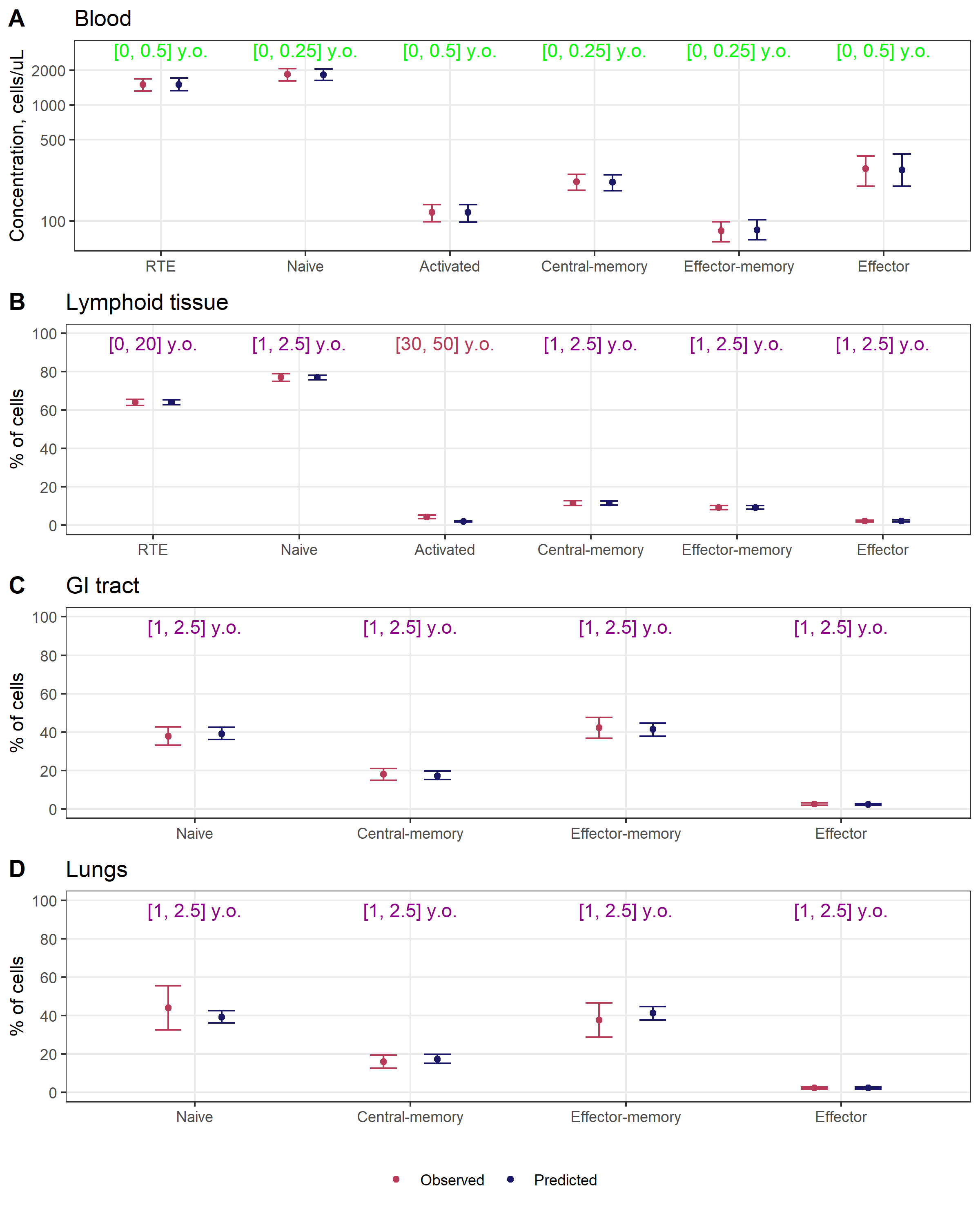
 **Supplementary Figure 1.** Observed vs. predicted values for homeostatic CD4+ T-lymphocyte cellular kinetic model for specific CD4+ T-lymphocyte subpopulations in blood (**A**), lymphoid tissue (**B**), gastro-intestinal tract (**C**) and lungs (**D**) (red and blue dots with error bars represent observed and predicted means with 95% CIs, respectively; values in parentheses represent the age ranges of the observed data used to calculate meta-analytical weighted averages which were taken as estimates for newborns when calibrating the model).

## Supplementary Figure 2


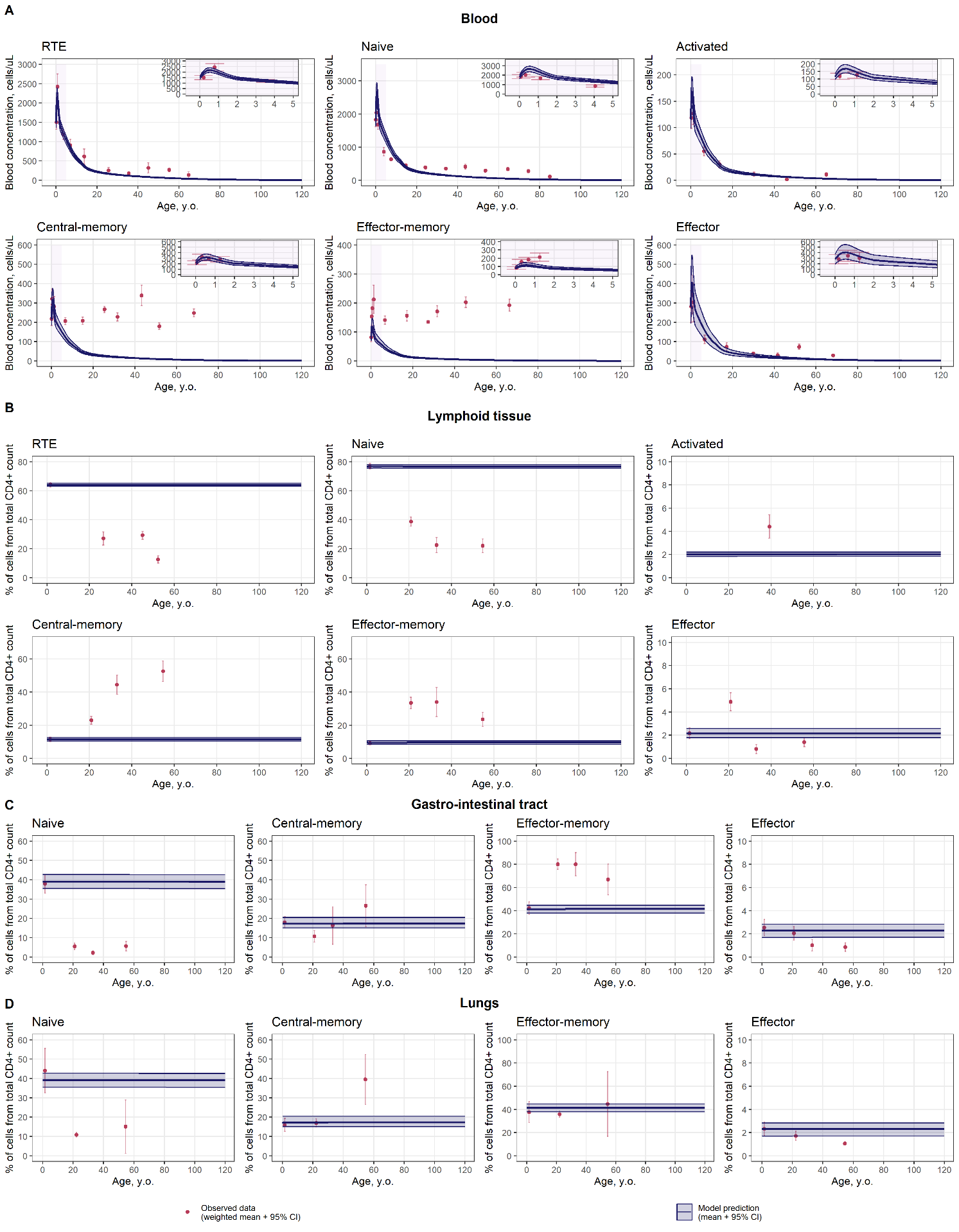


**Supplementary Figure 2**. Description of age-related cell dynamics by homeostatic CD4+ T-lymphocyte cellular kinetics model in blood (**A**), lymphoid tissue (**B**), gastro-intestinal tract (**C**) and lungs (**D**) (red dots represent observed data as meta-analytical weighted averages with 95% CIs; blue solid lines with shaded area represent predicted means with 95% CIs; purple shaded areas represent data description for neonates, infants and toddlers (0 to 5 years of age)).

## Supplementary Figure 3


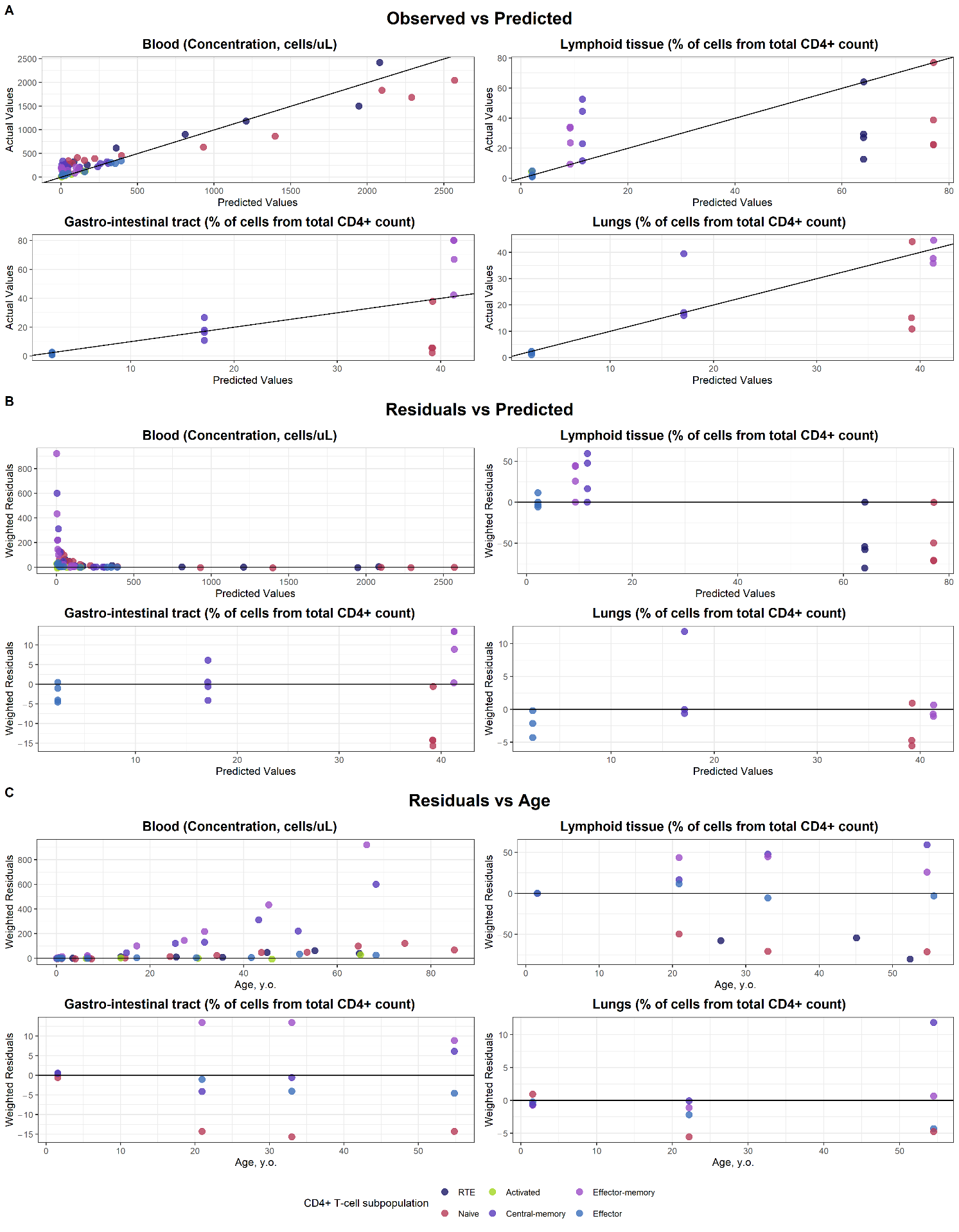


**Supplementary Figure 3.** Diagnostics of age-related change representation by homeostatic CD4+ T-lymphocyte cellular kinetics model (**A** – Observed data vs. Predicted values plot (data points are expected to scatter around the identity y=x line); **B** and **C** – Weighted residuals vs. Predicted values and Age, respectively (data points are expected to scatter around the horizontal zero-line); color of points represents specific subpopulation of CD4+ T-lymphocytes)

## Supplementary Figure 4


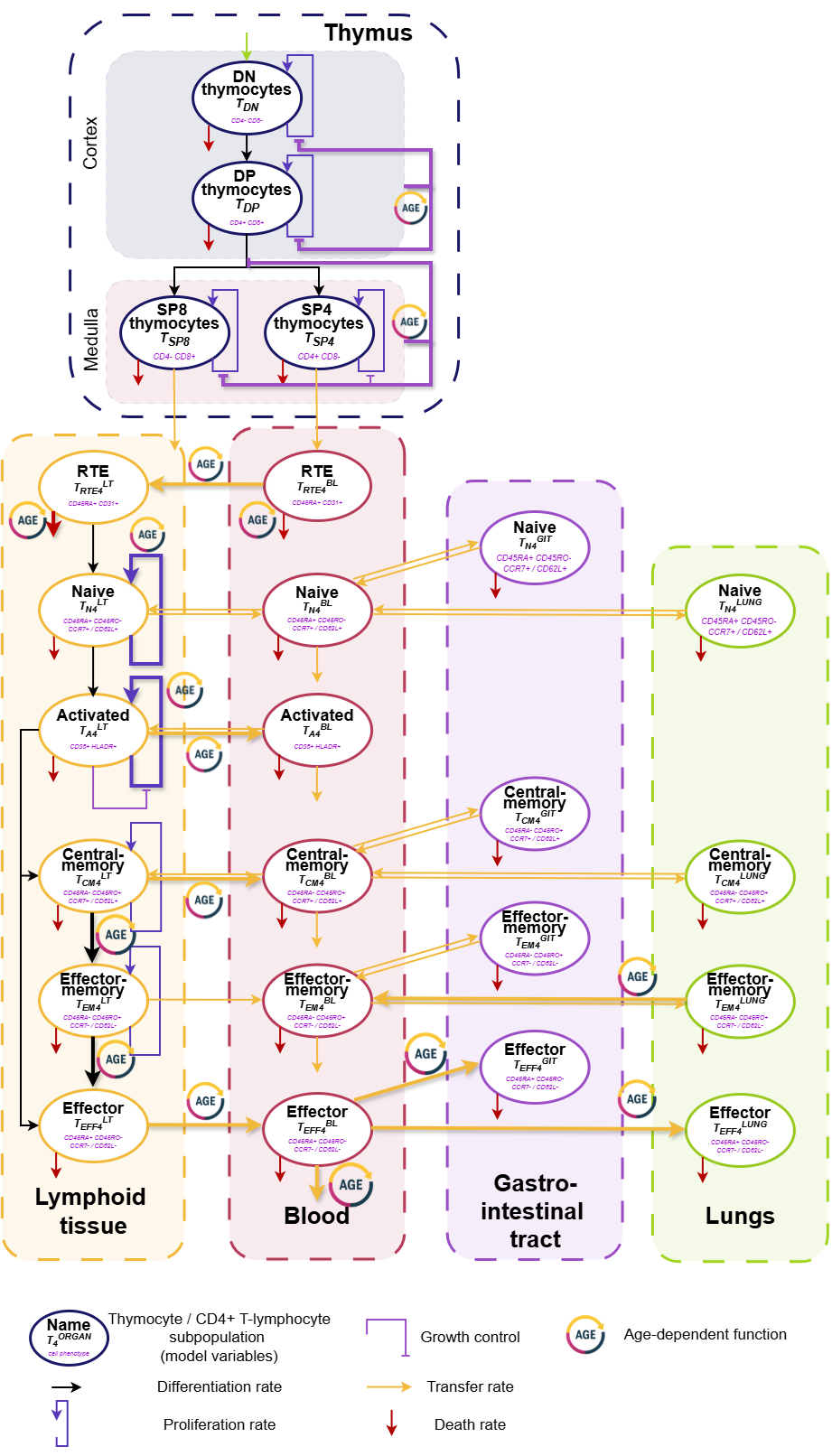


**Supplementary Figure 4.** Scheme of the homeostatic CD4+ T-lymphocyte cellular kinetics model with incorporation of age effect

## Supplementary Figure 5


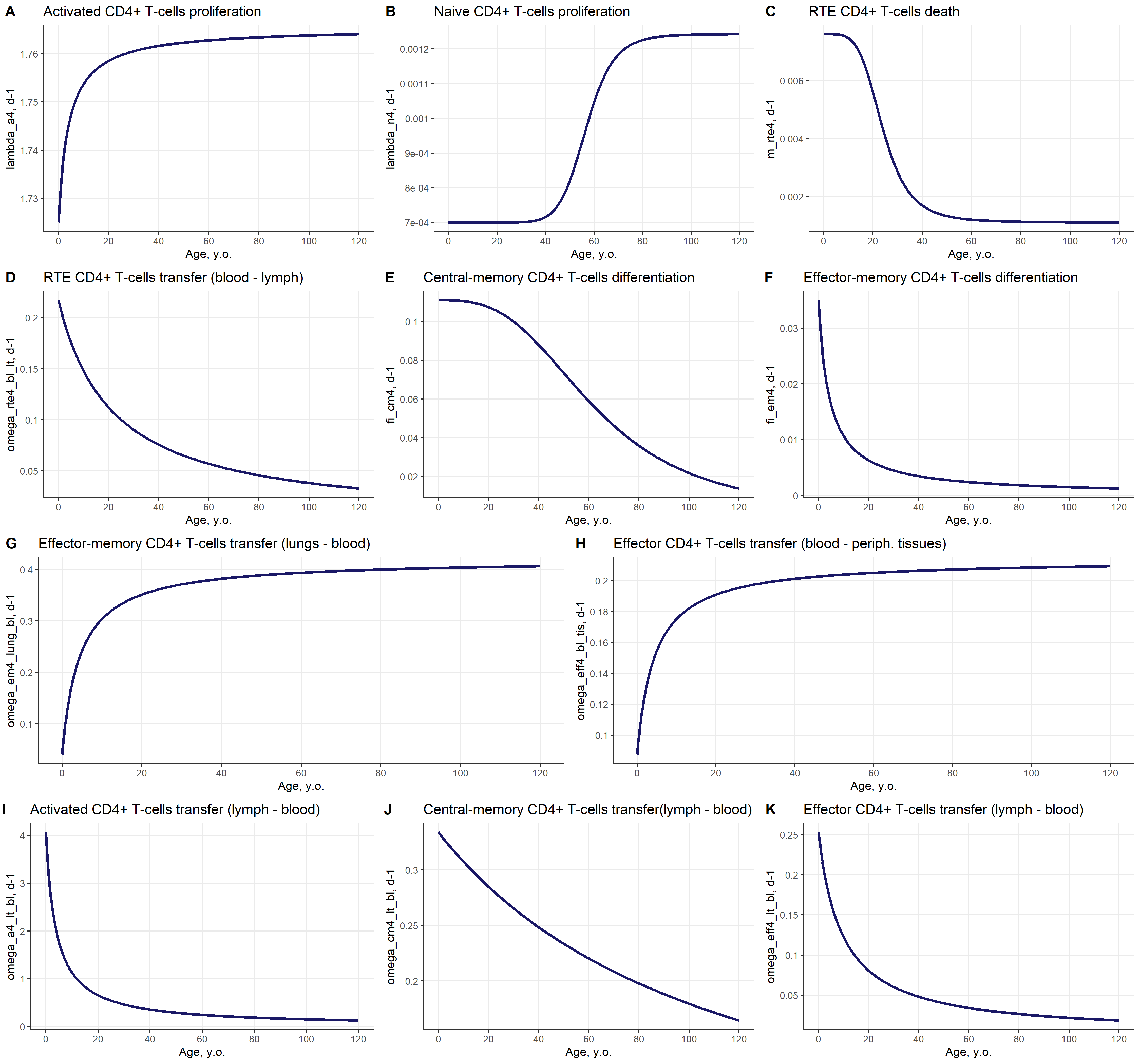


**Supplementary Figure 5.** Age dependencies of CD4+ T-lymphocyte homeostatic processes, incorporated in the model (**A** – activated CD4+ T-lymphocyte proliferation rate vs. age ($\lambda_{A4}\left( age \right)$); **B** – naïve CD4+ T-lymphocyte proliferation rate vs. age ($\lambda_{N4}\left( age \right)$); **C** – RTE CD4+ T-lymphocyte death rate vs. age ($\mu_{RTE4}\left( age \right)$); **D** – RTE CD4+ T-lymphocyte transfer rate from blood to lymphoid tissue vs. age ($\omega_{RTE4_{bl-lt}}\left( age \right)$); **E** – central-memory CD4+ T-lymphocyte differentiation rate vs. age ($\varphi_{CM4}\left( age \right)$); **F** – effector-memory CD4+ T-lymphocyte differentiation rate vs. age ($\varphi_{EM4}\left( age \right)$); **G** – effector-memory CD4+ T-lymphocyte transfer rate from lungs to blood vs. age ($\omega_{EM4_{lung-bl}}\left( age \right)$); **H** – effector CD4+ T-lymphocyte transfer rate from blood to gastro-intestinal tract ($\omega_{EFF4_{bl-git}}\left( age \right)$), lungs ($\omega_{EFF4_{bl-lung}}\left( age \right)$) and other peripheral tissues ($\omega_{EFF4_{bl-tis}}\left( age \right)$) vs. age; **I** – activated CD4+ T-lymphocyte transfer rate from lymphoid tissue to blood vs. age ($\omega_{A4_{lt-bl}}\left( age \right)$); **J** – central-memory CD4+ T-lymphocyte transfer rate from lymphoid tissue to blood vs. age ($\omega_{CM4_{lt-bl}}\left( age \right)$); **K** – effector CD4+ T-lymphocyte transfer rate from lymphoid tissue to blood vs. age ($\omega_{EFF4_{lt-bl}}\left( age \right)$).

## Supplementary Figure 6


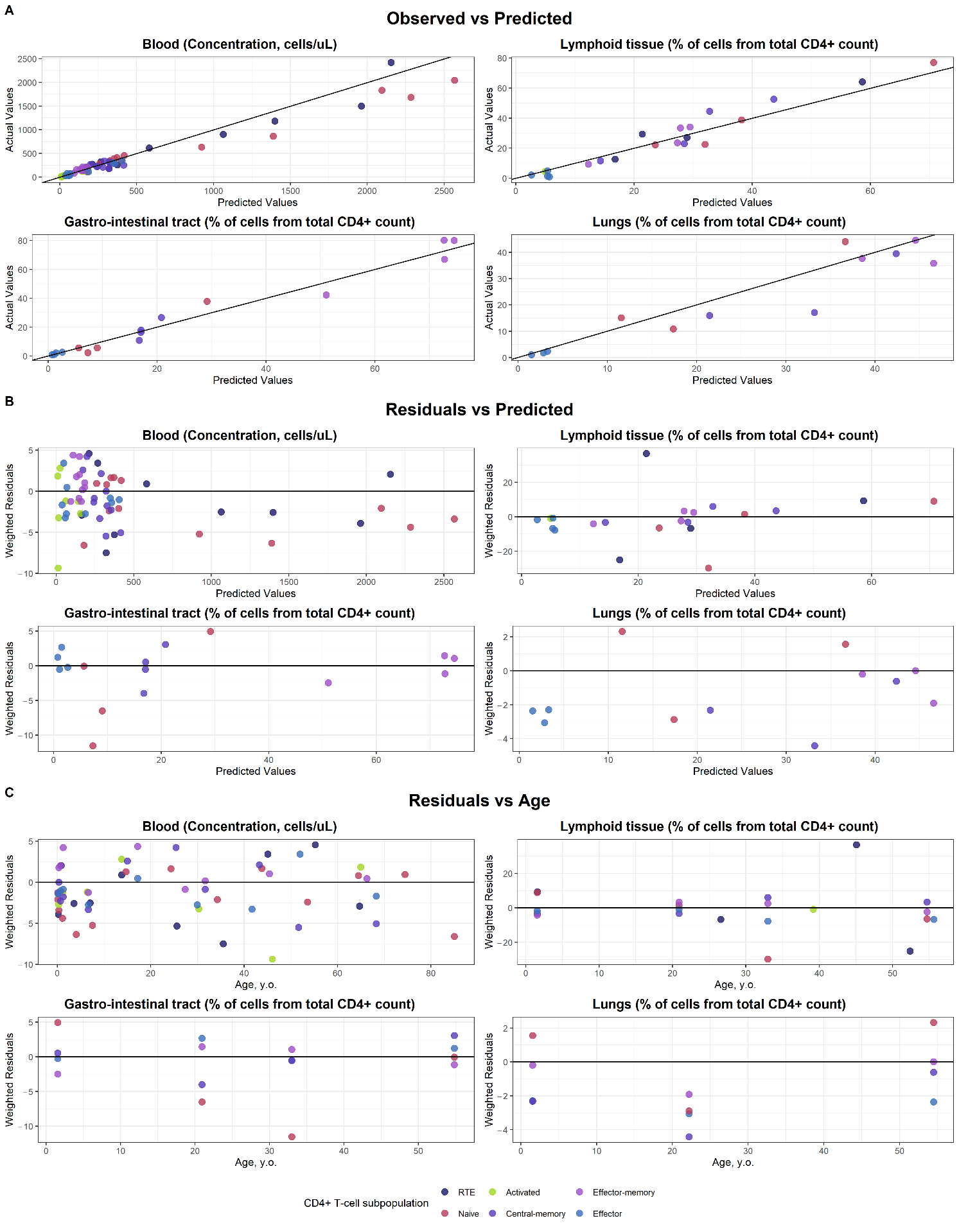


**Supplementary Figure 6.** Diagnostics of age-related change representation by homeostatic CD4+ T-lymphocyte cellular kinetics model, with incorporation of age effect (**A** – Observed data vs. Predicted values plot (data points are expected to scatter around the identity y=x line); **B** and **C** – Weighted residuals vs. Predicted values and Age, respectively (data points are expected to scatter around the horizontal zero-line); color of points represents specific subpopulation of CD4+ T-lymphocytes).

## Supplementary Figure 7


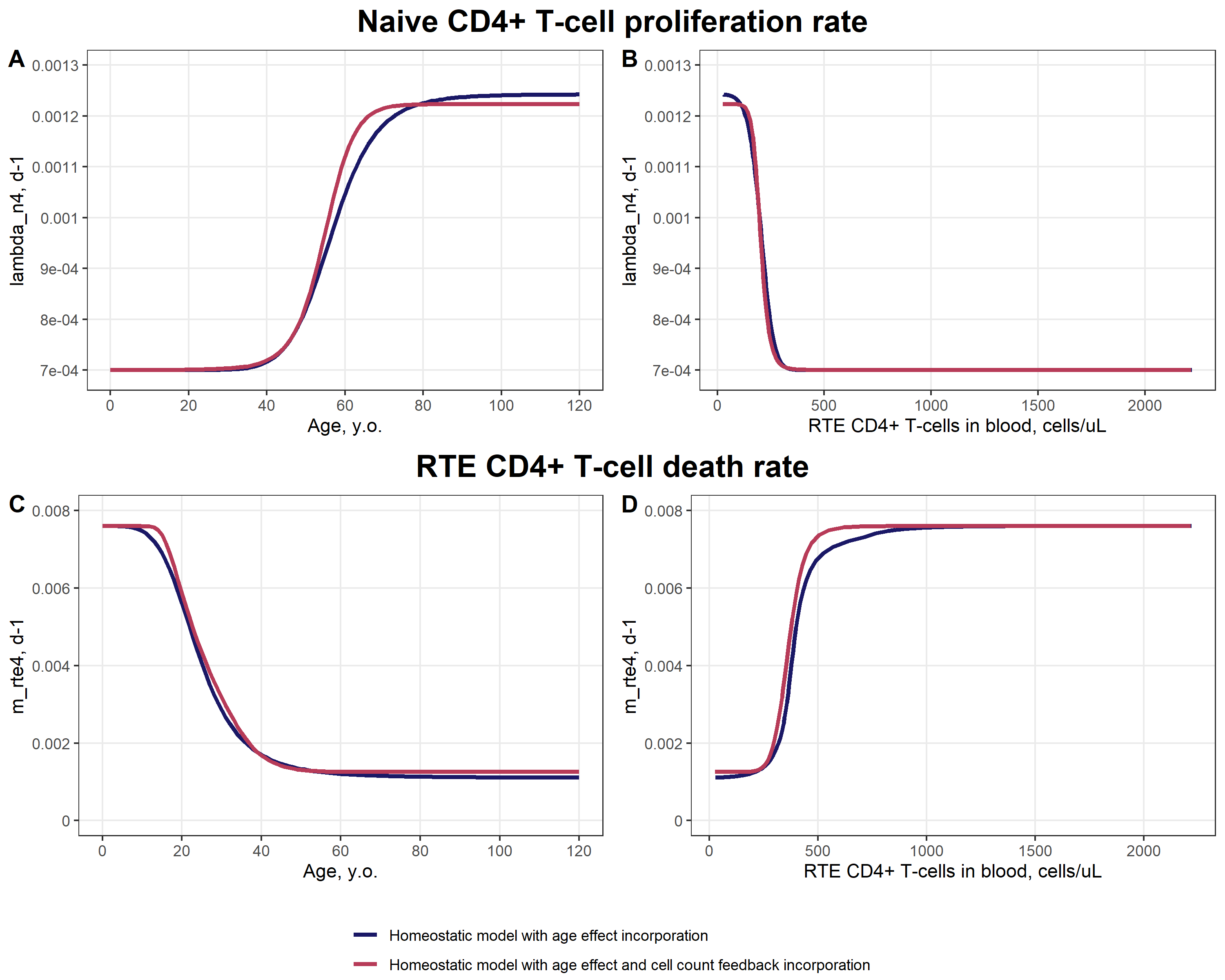


**Supplementary Figure 7**. Model predictions for naïve CD4+ T-lymphocyte proliferation rate and RTE CD4+ T-lymphocyte death rate dependencies with age (**A** and **C**) and with RTE CD4+ T-lymphocyte blood concentration (**B** and **D**); blue and red curves represents typical predictions of homeostatic CD4+ T-lymphocyte cellular kinetics model with age effect incorporation only and with both age effect and cellular feedback incorporation, respectively.

## Supplementary Figure 8


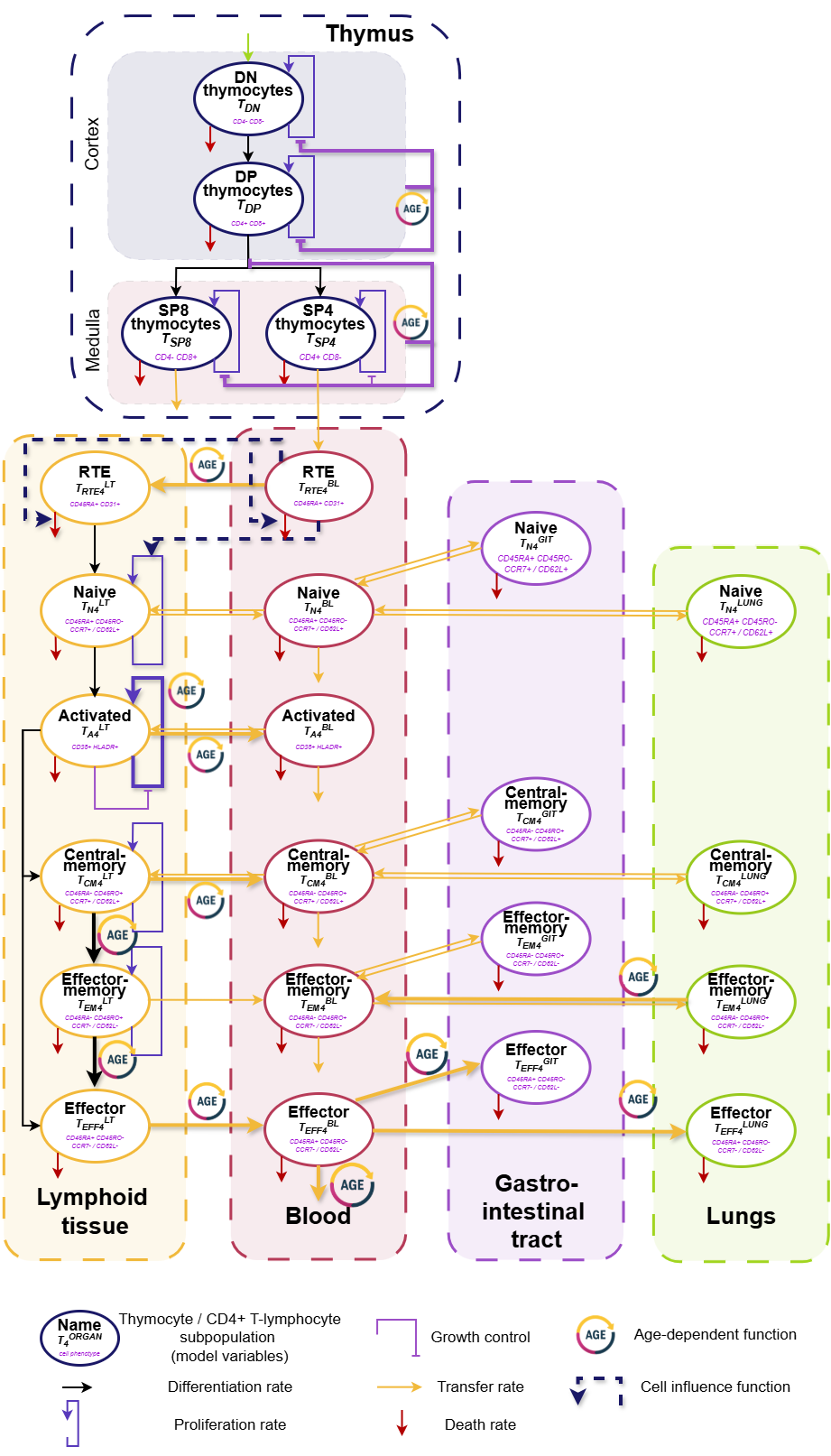


**Supplementary Figure 8.** Scheme of the homeostatic CD4+ T-lymphocyte cellular kinetics model with incorporation of age effect and cellular homeostatic feedback

## Supplementary Figure 9


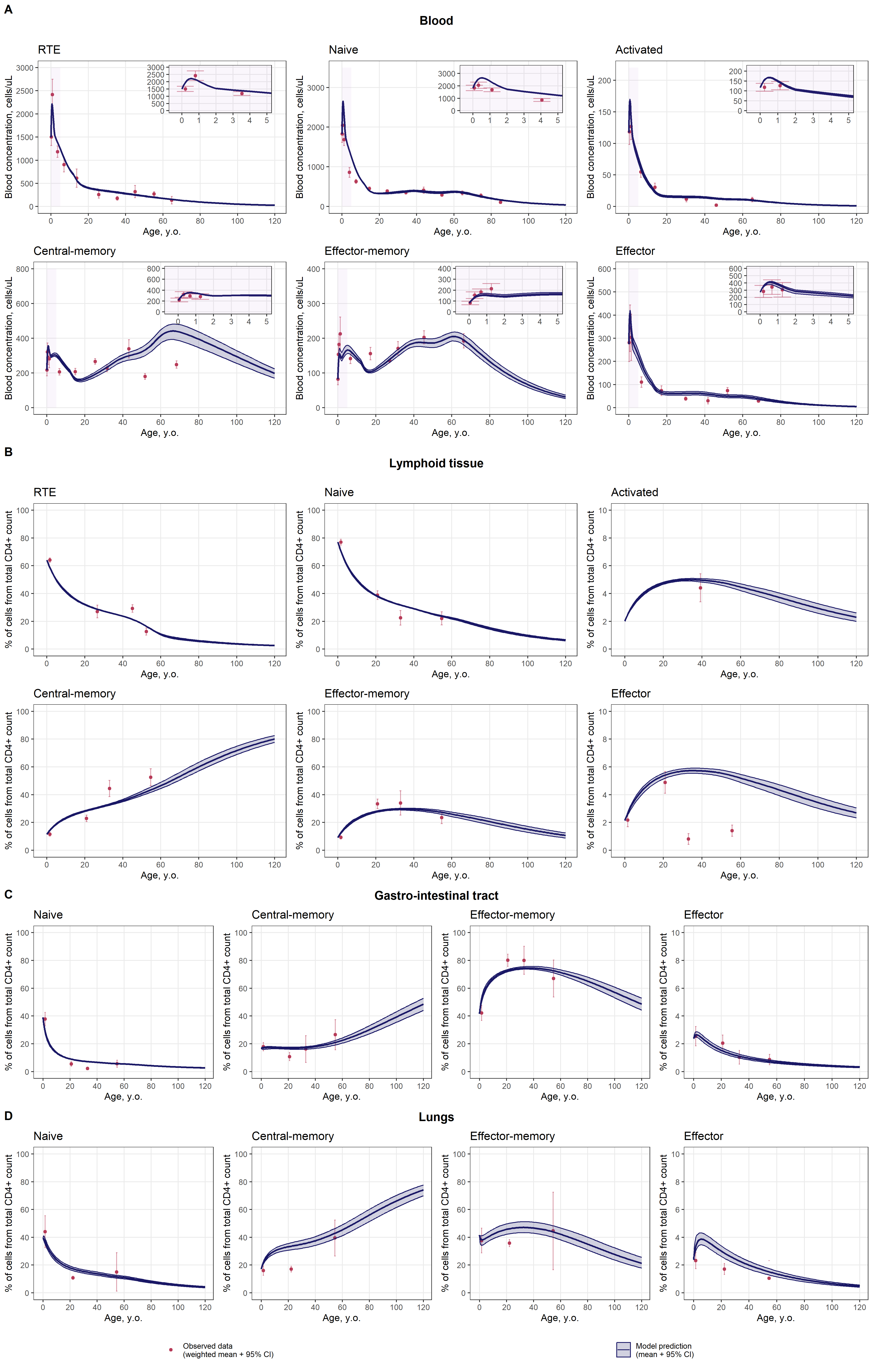


**Supplementary Figure 9.** Description of age-related cell dynamics by homeostatic CD4+ T-lymphocyte cellular kinetics model, with incorporation of age effect and cell count feedback in blood (**A**), lymphoid tissue (**B**), gastro-intestinal tract (**C**) and lungs (**D**) (red dots represent observed data as meta-analytical weighted averages with 95% CIs; blue solid lines with shaded area represent predicted means with 95% CIs; purple shaded areas represent data description for neonates, infants and toddlers (0 to 5 years of age); for better visualization, the percentage of effector cells is presented for the range 0 - 10%, while for other subpopulations - for 0 - 100%).

## Supplementary Figure 10


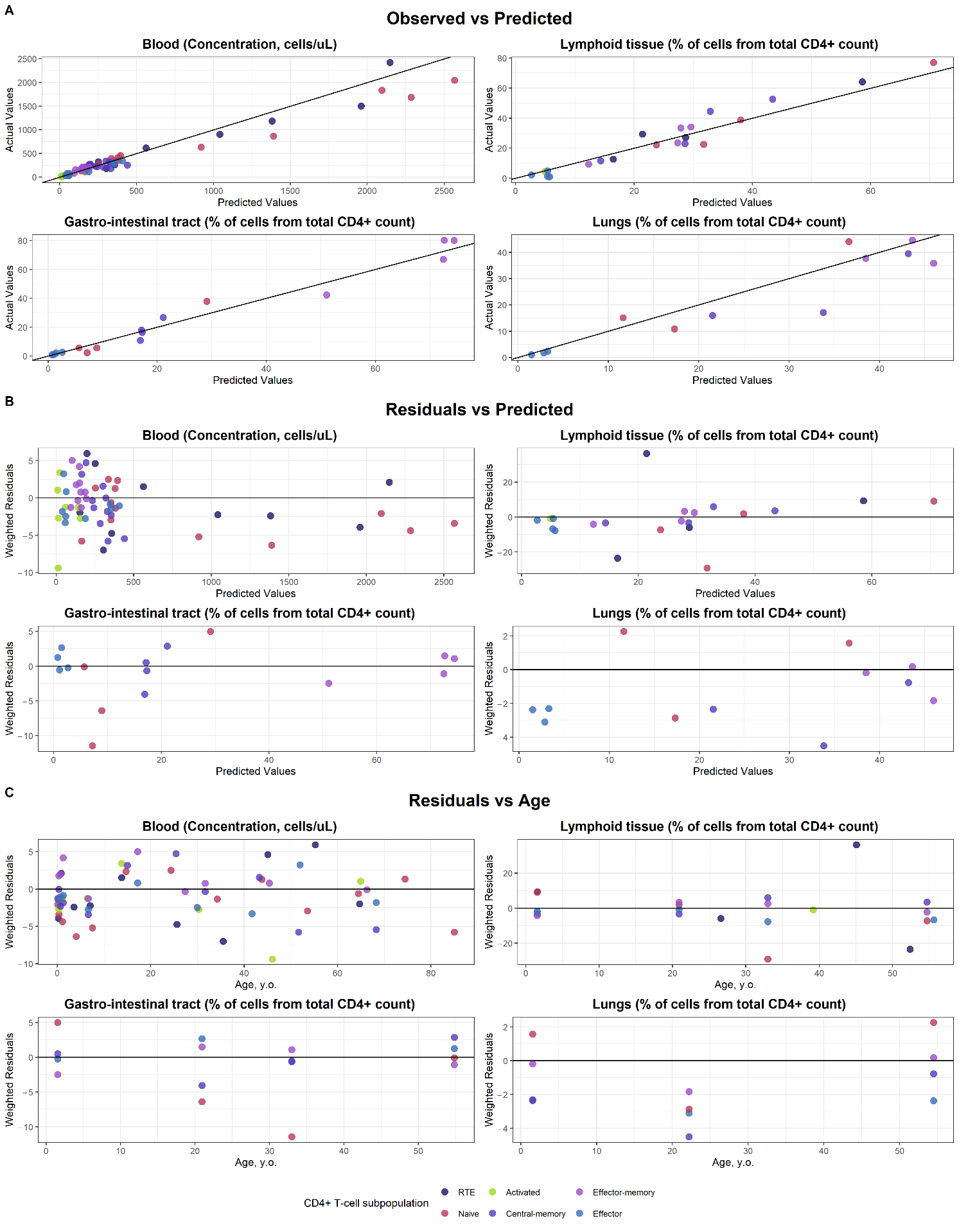


**Supplementary Figure 10.** Diagnostics of age-related changes representation by homeostatic CD4+ T-lymphocyte cellular kinetics model, with incorporation of age effect and cell count feedback (**A** – Observed data vs. Predicted values plot (data points are expected to scatter around the identity y=x line); **B** and **C** – Weighted residuals vs. Predicted values and Age, respectively (data points are expected to scatter around the horizontal zero-line); color of points represents specific subpopulation of CD4+ T-lymphocytes).

## Supplementary Figure 11


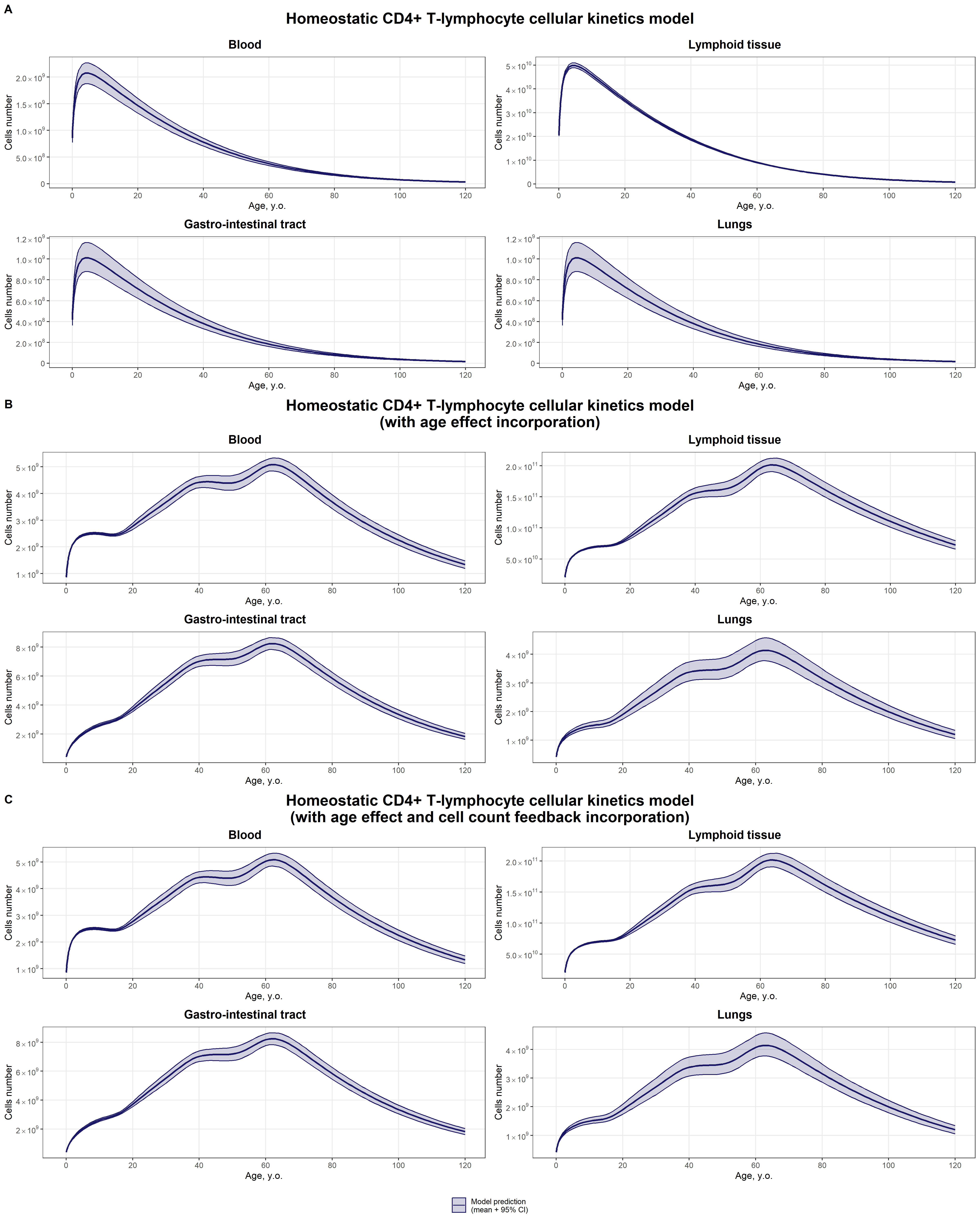


**Supplementary Figure 11.** Description of age-related cell dynamics for total CD4+ T-lymphocyte cell count in blood, lymphoid tissue, gastro-intestinal tract and lungs by homeostatic CD4+ T-lymphocyte cellular kinetics model (**A** – basic model calibrated on newborns data, **B** – with age-dependent function inclusion; **C** – with age- and cell count-dependent function inclusion) (blue solid lines with shaded area represent predicted means with 95% CIs).

## Supplementary Figure 12


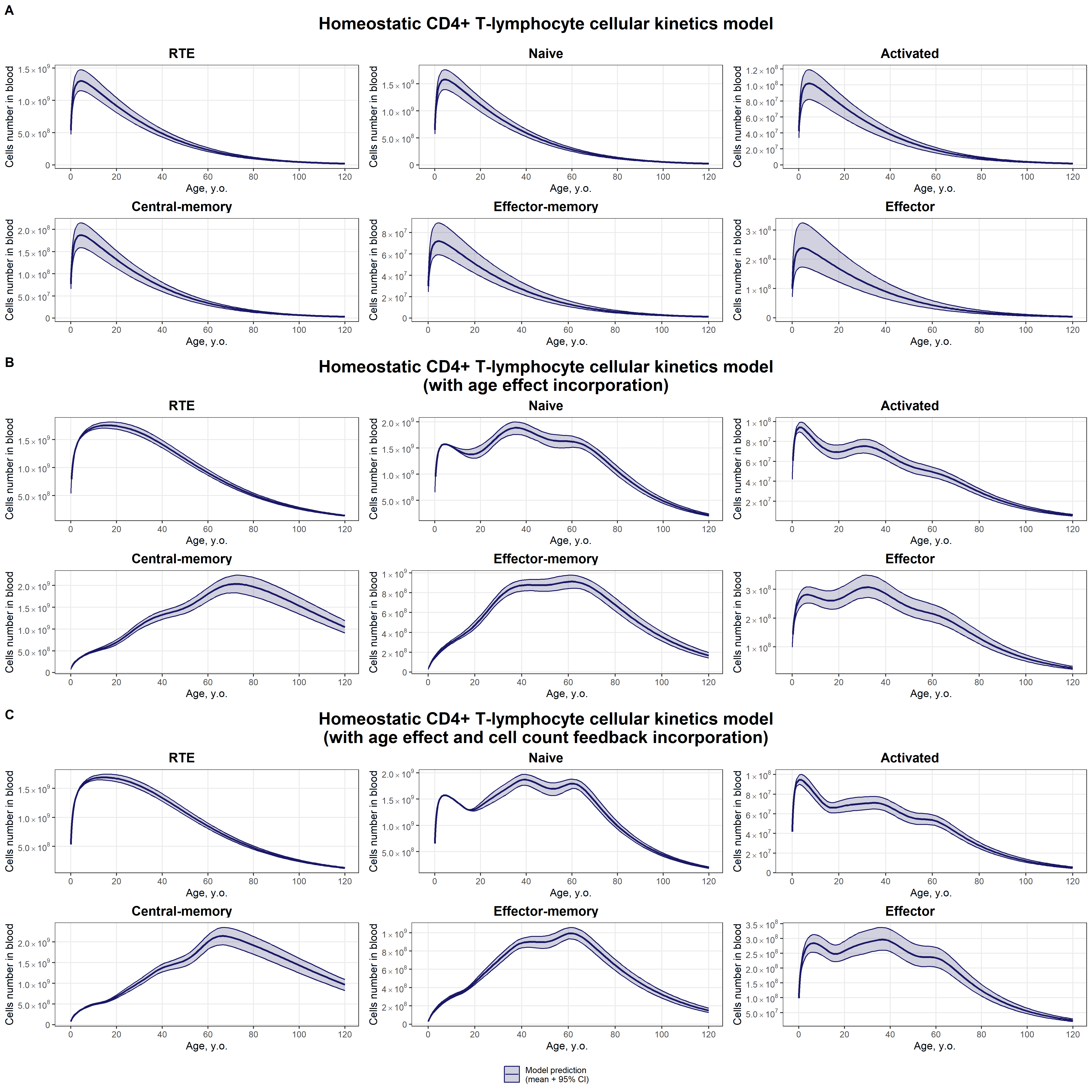


**Supplementary Figure 12.** Description of age-related cell dynamics for specific CD4+ T-lymphocyte subpopulations cell count in blood by homeostatic CD4+ T-lymphocyte cellular kinetics model (**A** – basic model calibrated on newborns data, **B** – with age-dependent function inclusion; **C** – with age- and cell count-dependent function inclusion) (blue solid lines with shaded area represent predicted means with 95% CIs).

## Supplementary Figure 13


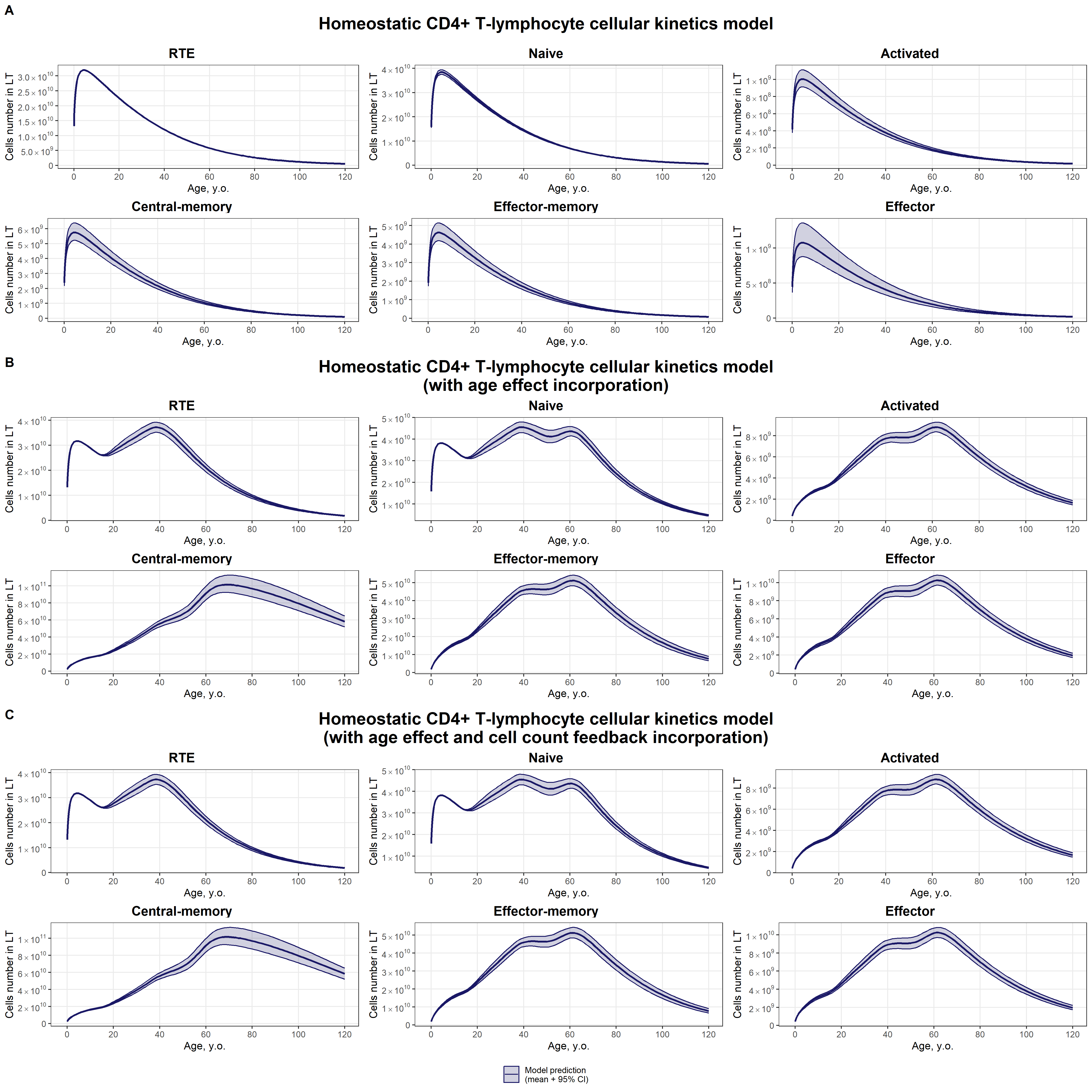


**Supplementary Figure 13.** Description of age-related cell dynamics for specific CD4+ T-lymphocyte subpopulations cell count in lymphoid tissue by homeostatic CD4+ T-lymphocyte cellular kinetics model (**A** – basic model calibrated on newborns data, **B** – with age-dependent function inclusion; **C** – with age- and cell count-dependent function inclusion) (blue solid lines with shaded area represent predicted means with 95% CIs),

## Supplementary Figure 14


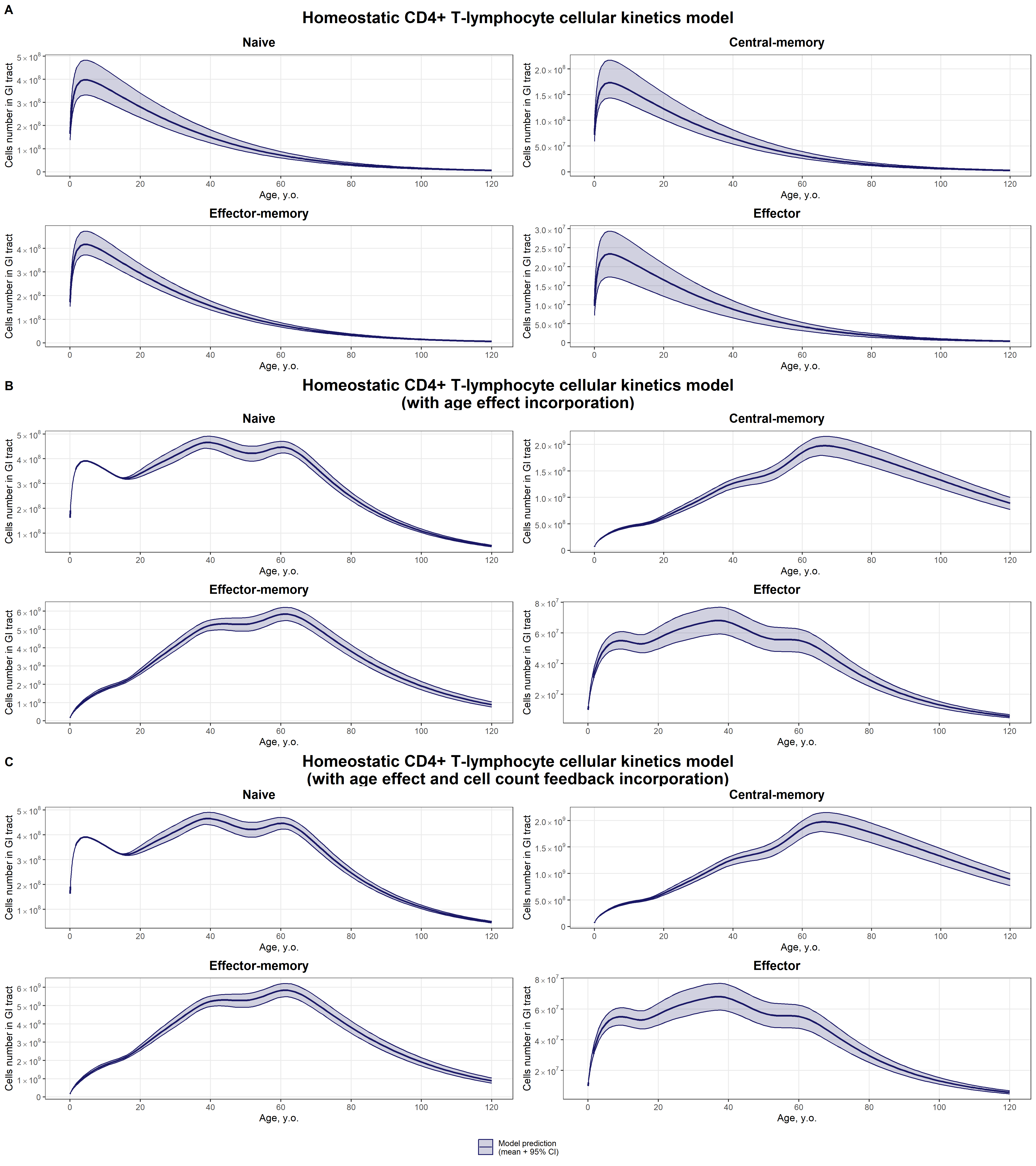


**Supplementary Figure 14.** Description of age-related cell dynamics for specific CD4+ T-lymphocyte subpopulations cell count in gastro-intestinal tract by homeostatic CD4+ T-lymphocyte cellular kinetics model (**A** – basic model calibrated on newborns data, **B** – with age-dependent function inclusion; **C** – with age- and cell count-dependent function inclusion) (blue solid lines with shaded area represent predicted means with 95% CIs).

## Supplementary Figure 15


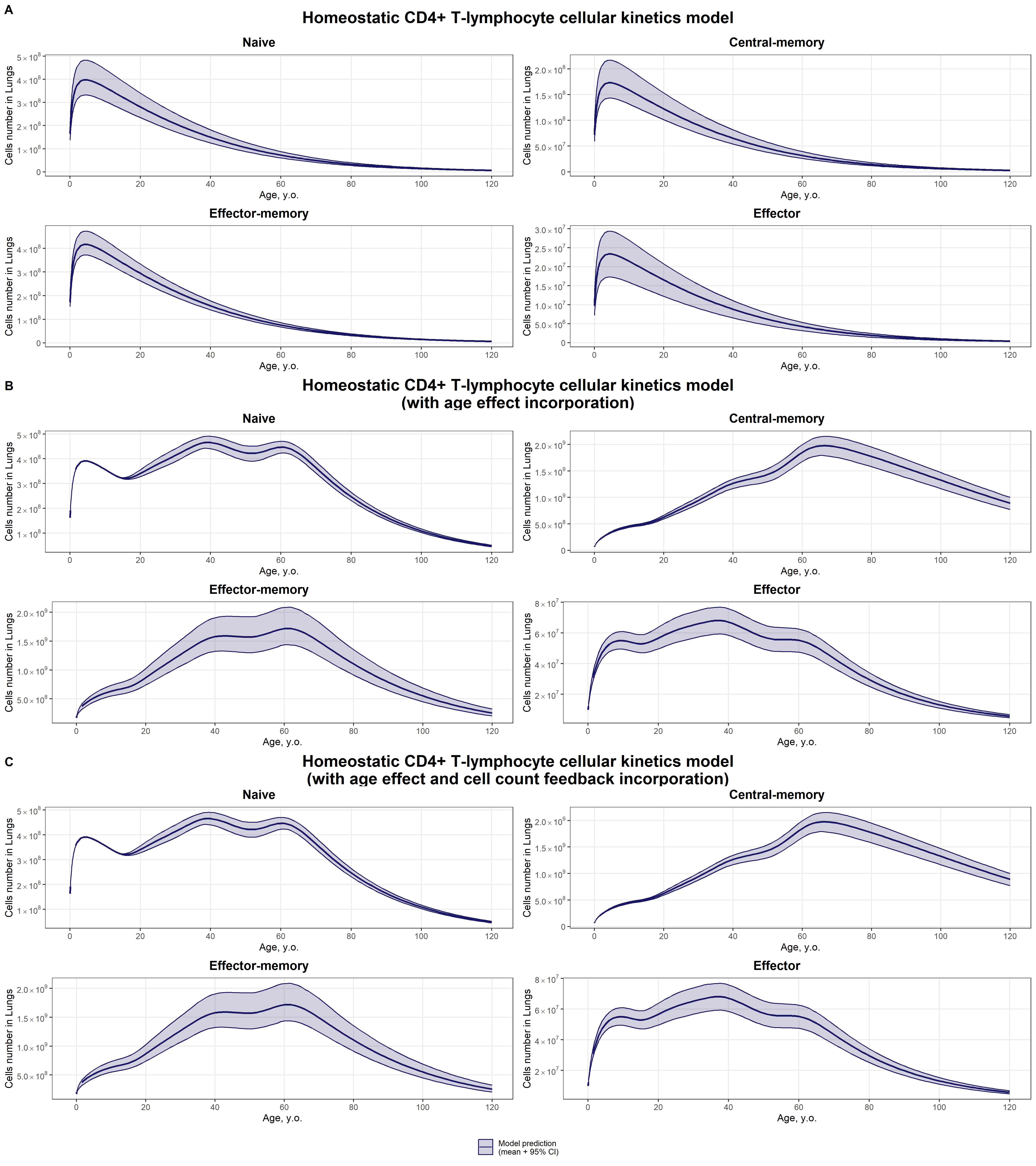


**Supplementary Figure 15.** Description of age-related cell dynamics for specific CD4+ T-lymphocyte subpopulations cell count in lungs by homeostatic CD4+ T-lymphocyte cellular kinetics model (**A** – basic model calibrated on newborns data, **B** – with age-dependent function inclusion; **C** – with age- and cell count-dependent function inclusion) (blue solid lines with shaded area represent predicted means with 95% CIs).

## Supplementary Figure 16


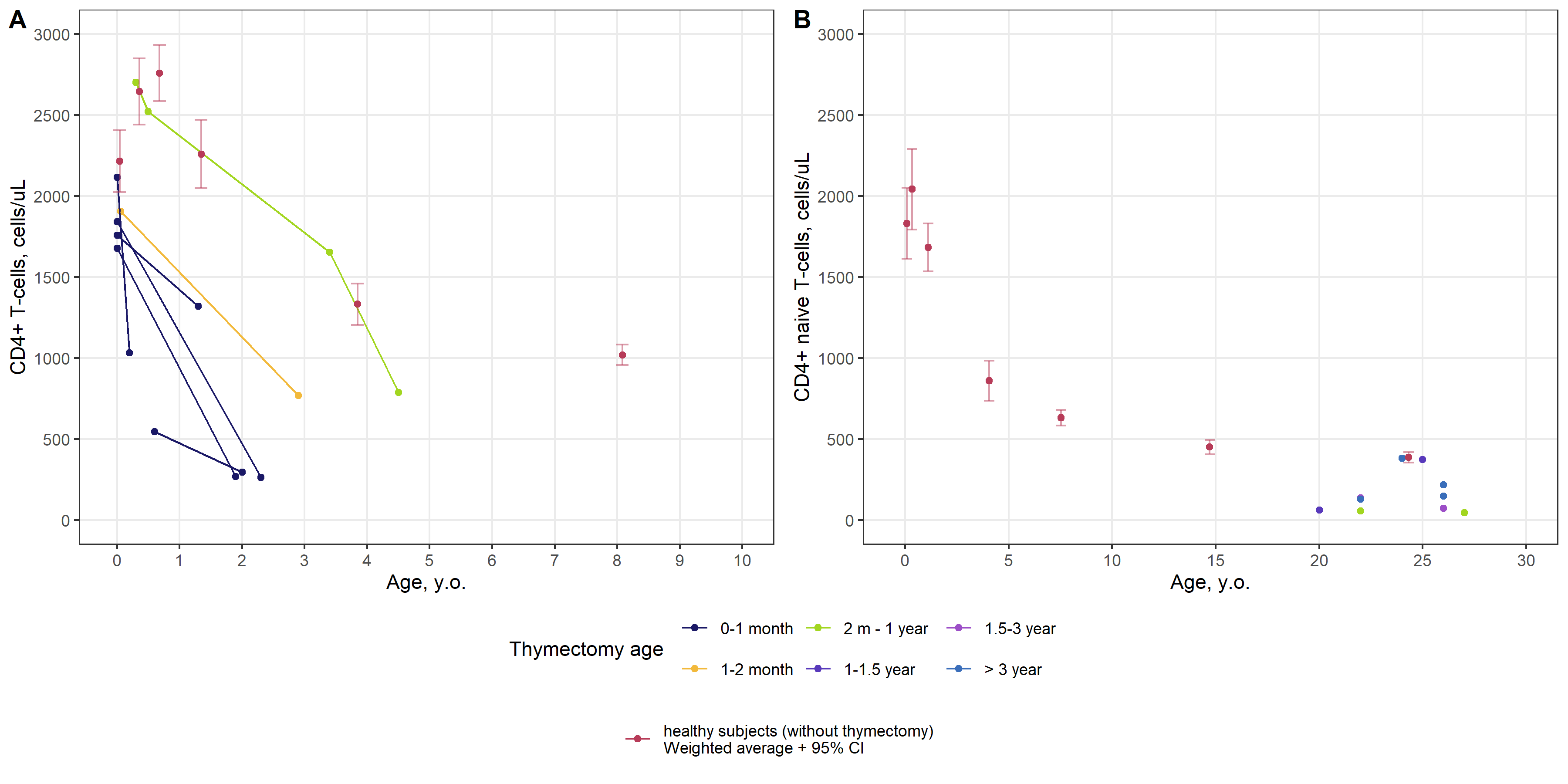


**Supplementary Figure 16**. CD4+ T-lymphocytes (A) and CD4+ naïve T-lymphocytes (B) blood concentration individual data from patients who underwent complete thymectomy (color represents the group of subjects depending on the age of thymus gland removal; red dots depict the corresponding concentrations for healthy non-thymectomized subjects).

## Supplementary Figure 17


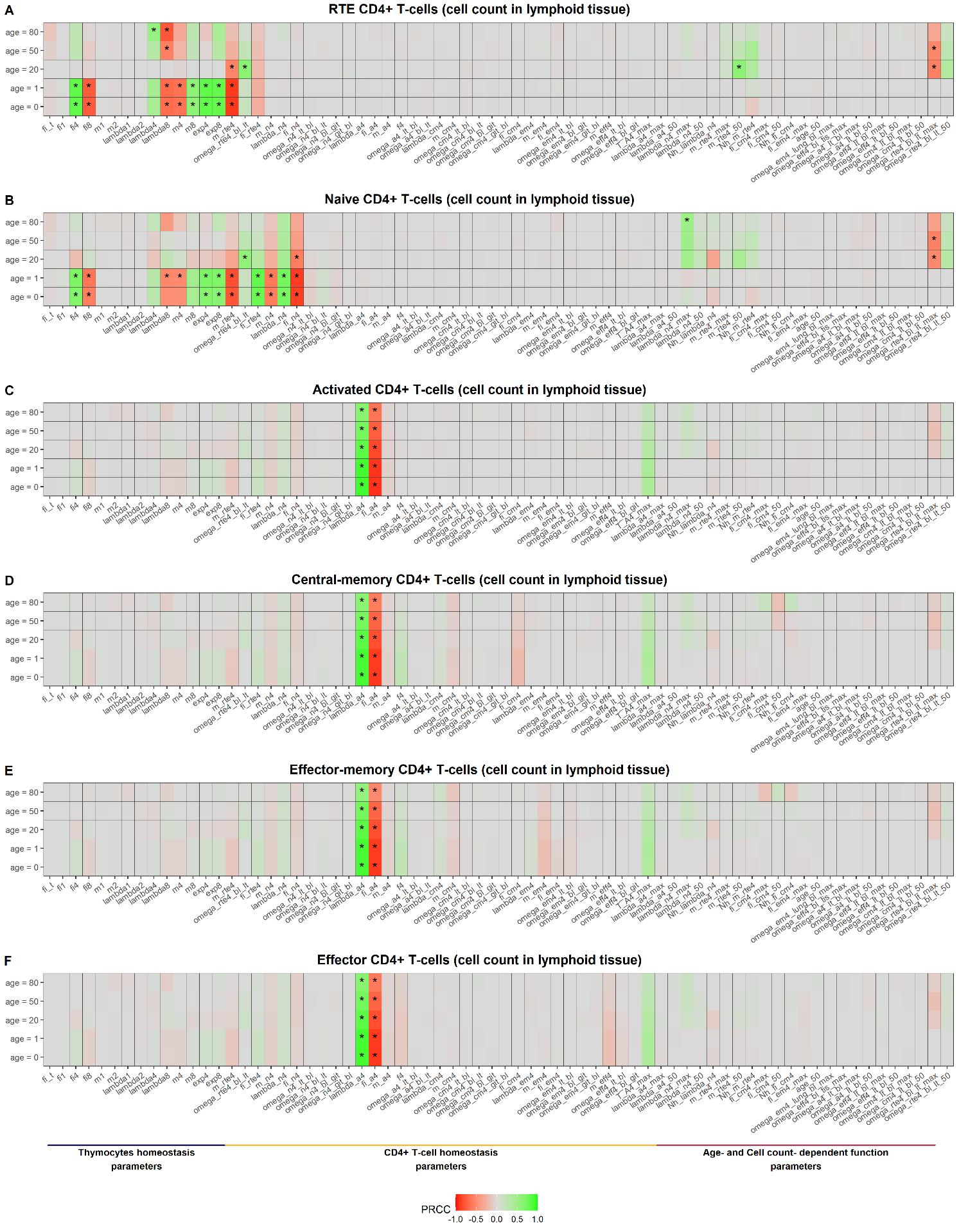


**Supplementary Figure 17.** Global sensitivity analysis using a PRCC-based method: cell count of RTE (A), naïve (B), activated (C), central-memory (D), effector-memory (E) and effector (F) CD4+ T-lymphocytes in lymphoid tissue for 5 levels of age (0, 1, 20, 50, 80 years old) (asterisk (*) indicates |PRCC| ≥ 0.5).

## Supplementary Figure 18


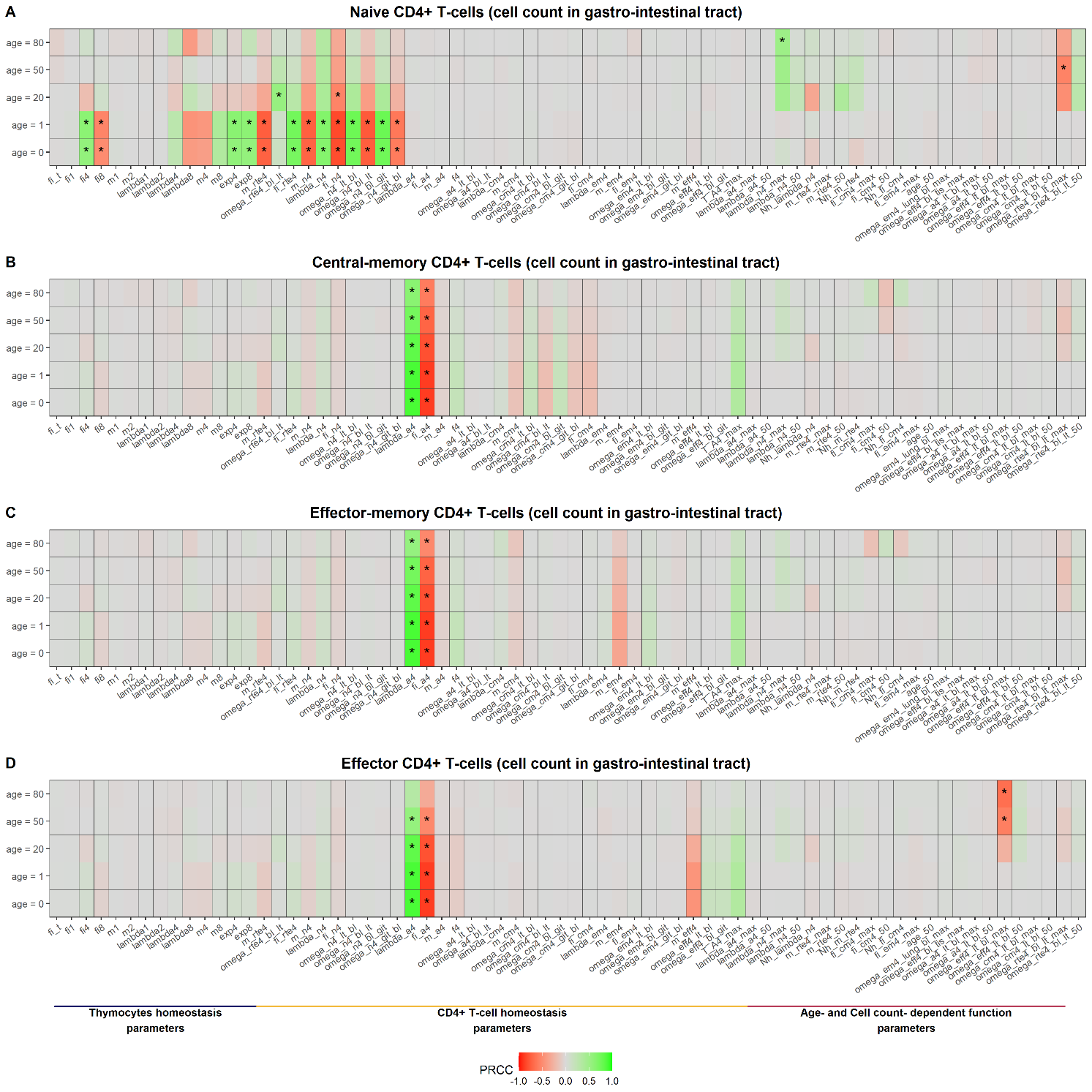


**Supplementary Figure 18.** Global sensitivity analysis using a PRCC-based method: cell count of naïve (A), central-memory (B), effector-memory (C) and effector (D) CD4+ T-lymphocytes in the gastro-intestinal tract for 5 levels of age (0, 1, 20, 50, 80 years old) (asterisk (*) indicates |PRCC| ≥ 0.5).

## Supplementary Figure 19


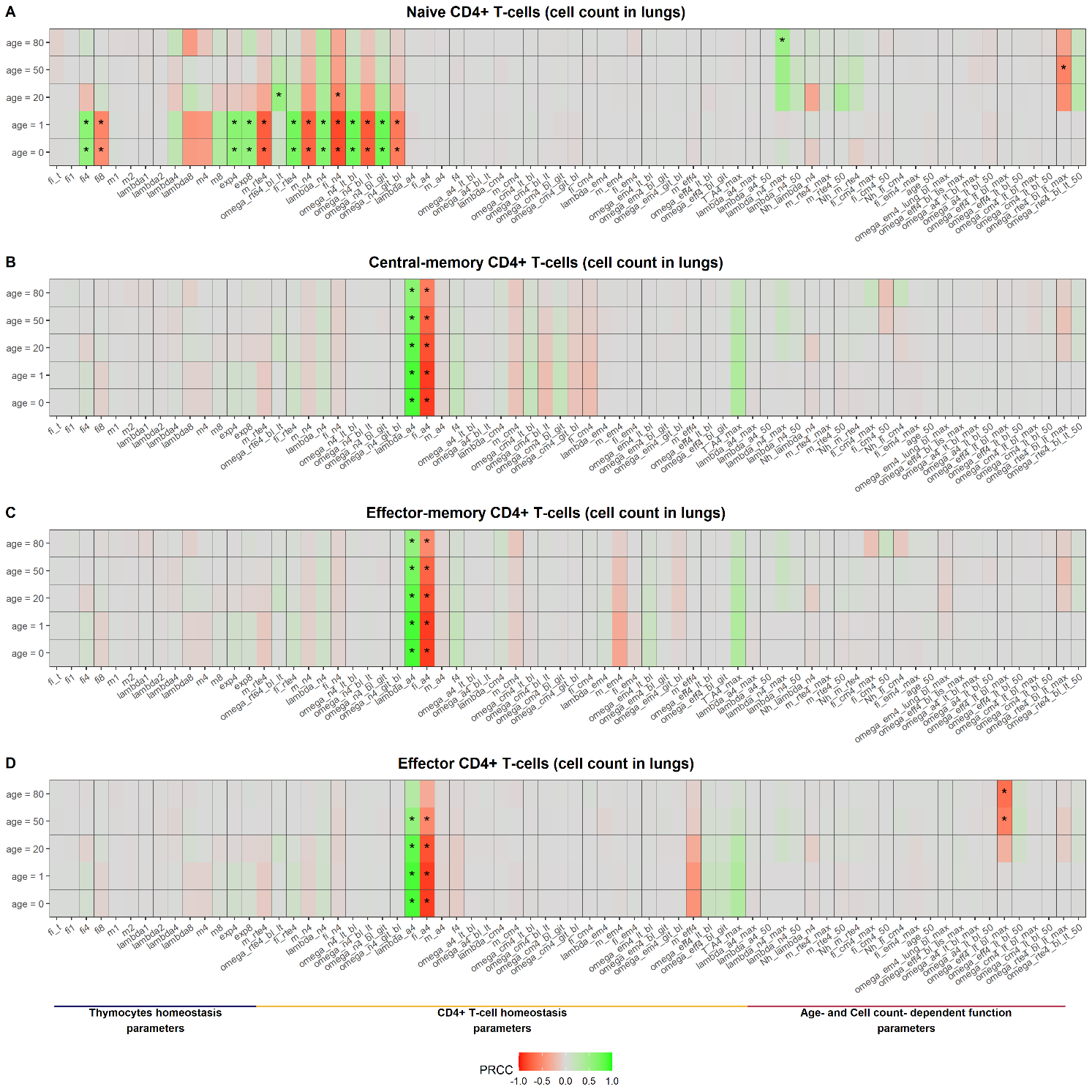


**Supplementary Figure 19.** Global sensitivity analysis using a PRCC-based method: cell count of naïve (A), central-memory (B), effector-memory (C) and effector (D) CD4+ T-lymphocytes in lungs for 5 levels of age (0, 1, 20, 50, 80 years old) (asterisk (*) indicates |PRCC| ≥ 0.5)

## Supplementary Figure 20


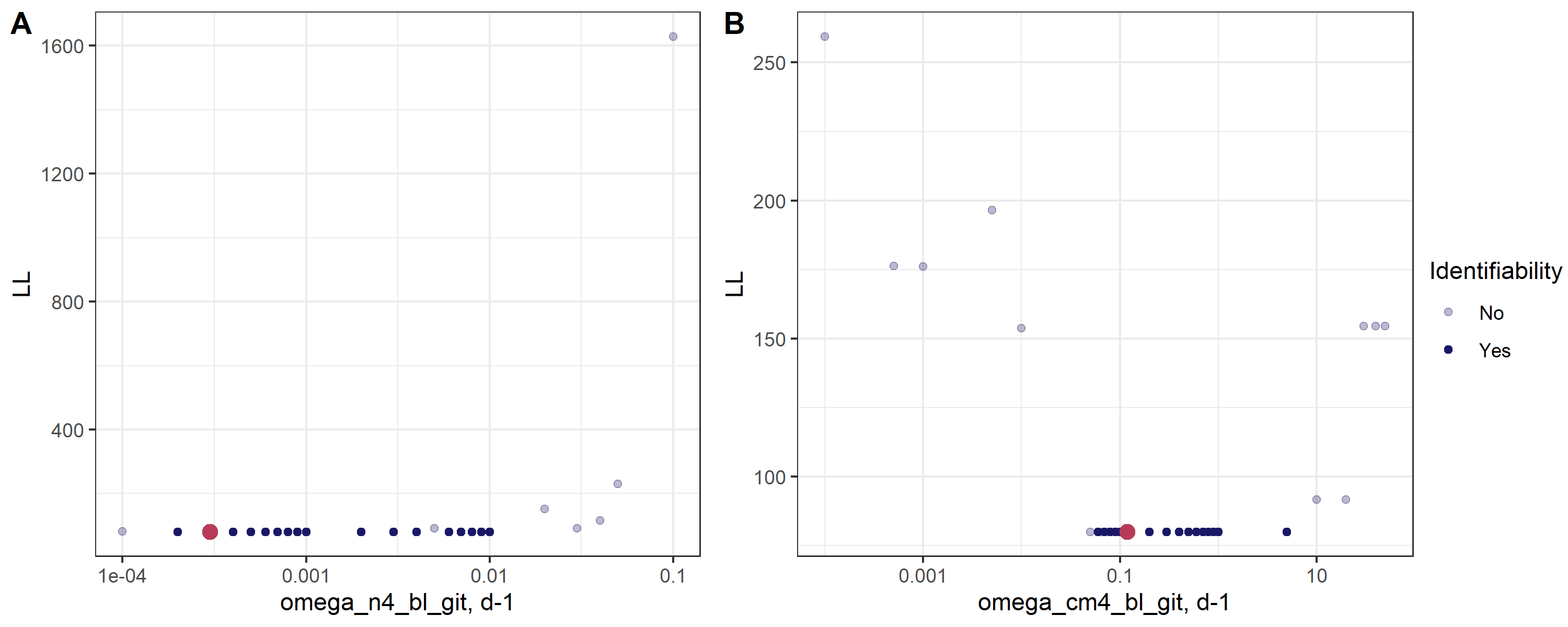


**Supplementary Figure 20.** Likelihood function profiling results for $\omega_{N4_{bl-git}}$ (A) and $\omega_{CM4_{bl-git}}$ (B) parameters (blue dots represent the model runs; red dot – homeostatic model calibration results)

## Supplementary Figure 21


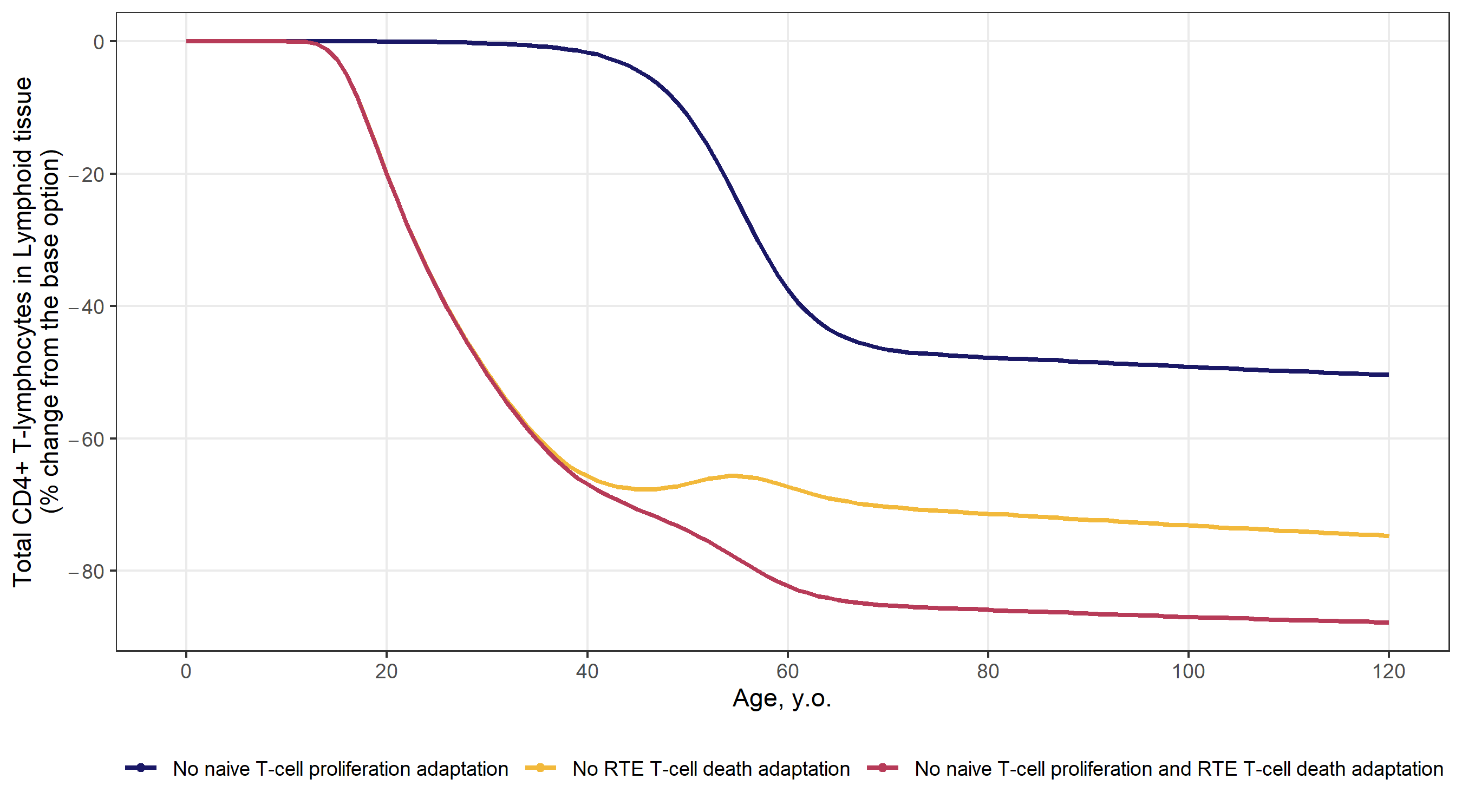
**Supplementary Figure 21.** Evaluation of the effect of removing homeostatic adaptations on CD4+ T-cell numbers in lymphoid organs across the lifespan. Three scenarios are shown: no adaptation of naïve T-cell proliferation (blue curve), no adaptation of the RTE death rate (yellow curve), and no adaptations (red curve). Scenarios were implemented by setting $\lambda_{{N4}_{max}}^{RTE4}$= 0 and/or $\mu_{{RTE4}_{max}}^{RTE4}$= 0, depending on the condition.

## Supplementary Figure 22





**Supplementary Figure 22.** **Pairwise parameter interaction maps for CD4+ T-cell homeostasis in lymphoid tissue across age**: (A) Naïve CD4+ T-cell proliferation rate ($\lambda_{N4}$) vs. Central-memory CD4+ T-cell differentiation rate ($\varphi_{CM4}$); (B) Naïve CD4+ T-cell proliferation rate ($\lambda_{N4}$) vs. Effector-memory CD4+ T-cell differentiation rate ($\varphi_{EM4}$); (C) Central-memory CD4+ T-cell differentiation rate ($\varphi_{CM4}$) vs. Effector-memory CD4+ T-cell differentiation rate ($\varphi_{EM4}$); (D) Activated CD4+ T-cell proliferation rate ($\lambda_{A4}$) vs. Central-memory CD4+ T-cell differentiation rate ($\varphi_{CM4}$); (E) Activated CD4+ T-cell proliferation rate ($\lambda_{A4}$) vs. Effector-memory CD4+ T-cell differentiation rate ($\varphi_{EM4}$); (F) Activated CD4+ T-cell proliferation rate ($\lambda_{A4}$) vs. Activated CD4+ T-cell differentiation rate ($\varphi_{A4}$). Black contour lines indicate equally predicted CD4+ T-cell numbers.

# Model code

DESCRIPTION:

CD4+ T-lymphocyte cellular kinetics model (stage A)

[LONGITUDINAL]

input = {m_rte4, omega_rte4_bl_lt, fi_rte4, m_n4, lambda_n4, fi_n4, omega_n4_lt_bl, omega_n4_bl_lt, omega_n4_bl_git, omega_n4_git_bl, lambda_a4, fi_a4, m_a4, f4, omega_a4_lt_bl, omega_a4_bl_lt, lambda_cm4, m_cm4, omega_cm4_lt_bl, omega_cm4_bl_lt, omega_cm4_bl_git, omega_cm4_git_bl, fi_cm4, lambda_em4, m_em4, fi_em4, omega_em4_lt_bl, omega_em4_bl_git, omega_em4_git_bl, m_eff4, omega_eff4_lt_bl, omega_eff4_bl_git, T_A4_max, age}

age = {use = regressor}

EQUATION:

odeType = stiff

; Thymocytes homeostasis submodel parameters

fi_t = 480000; ;cells/day

fi1 = 0.21; ;1/day

fi4 = 0.8; ;1/day

fi8 = 0.3; ;1/day

m1 = 0.056; ;1/day

m2 = 0.5; ;1/day

lambda1 = 1.67; ;1/day

lambda2 = 3.125; ;1/day

lambda4 = 0.22; ;1/day

lambda8 = 0.22; ;1/day

m4 = 0.005; ;1/day

m8 = 0.005; ;1/day

exp4 = 0.06; ;1/day

exp8 = 0.06; ;1/day

; Thymus involution submodel parameters

; thymus wet weight vs age parameters

WW_BL = 11.0948; ;g

WW_EC50_1 = 1.0909; ;years

WW_max = 24.4468; ;g

WW_EC50_2 = 52.2717; ;years

; thymic epithelial space vs age parameters

b_TES = 93.344; ;%

k_TES = -0.035; ;%/years

; cortico-medullary ratio vs age parameters

b_CM = 2.77; ; -

k_CM = -0.035; ; 1/years

; thymic density vs age parameters

b_Dens = 1.014; ;g/cm3

k_Dens = -0.001; ;g/(cm3*year)

; thymic volume vs age parameters

b_Vol = 25.18; ;cm3

k_Vol = -0.056; ;cm3/year

; Blood volume vs age parameters

TM50_WT1 = 38.5; ;years

WTmax1 = 2.76; ;kg

hill1_WT1 = 12.9; ;-

hill2_WT1 = 2.74; ;-

TM50_WT2 = 2.1; ;years

WTmax2 = 16.4; ;kg

hill1_WT2 = 2.04; ;-

hill2_WT2 = 1; ;-

TM50_WT3 = 12.4; ;years

WTmax3 = 40.2; ;kg

hill1_WT3 = 2.87; ;-

hill2_WT3 = 0; ;-

WTmax4 = 33.6; ;kg

THALF_WT4 = 3.61; ;years

k_BV = 62.73817; ;mL/kg

BV_BL = 357.30836; ;mL

; CD4+ T-lymphocyte cellular kinetics explicit functions

omega_n4_bl_lung = omega_n4_bl_git; ;1/day

omega_n4_bl_tis = omega_n4_bl_git; ;1/day

omega_n4_lung_bl = omega_n4_git_bl; ;1/day

omega_a4_bl_tis = omega_n4_bl_git;

omega_cm4_bl_lung = omega_cm4_bl_git; ;1/day

omega_cm4_bl_tis = omega_cm4_bl_git; ;1/day

omega_cm4_lung_bl = omega_cm4_git_bl; ;1/day

omega_em4_bl_lung = omega_em4_bl_git; ;1/day

omega_em4_bl_tis = omega_em4_bl_git; ;1/day

omega_em4_lung_bl = omega_em4_git_bl; ;1/day

omega_eff4_bl_lung = omega_eff4_bl_git; ;1/day

omega_eff4_bl_tis = omega_eff4_bl_git; ;1/day

; Thymus involution submodel equations

; thymus wet weight vs age

WW_g = (WW_BL + WW_max*age/(age + WW_EC50_1)) * (1 - age/(age + WW_EC50_2));

WW = WW_g / WW_BL;

; TES and CM vs age

TES = b_TES * exp(k_TES*age) / 100;

CM = b_CM * exp(k_CM*age);

; Maximal capacity of thymic cortex and medulla (Tcort and Tmed) vs age

T0 = 1026.05 * 10^6 * WW_BL; ; calculated according to cell density in thymus in newborns (Bertho et al., 1997)

Tcort = CM / (CM + 1) * WW * TES * T0;

Tmed = 1 / (CM + 1) * WW * TES * T0;

; Thymic density and volume vs age

Dens = (b_Dens + k_Dens * age)

Vol = (b_Vol + k_Vol * age)

; Relative volume of thymic cortex and medulla vs age

Tcort_relvol = (CM / (CM + 1)) * (WW_g * TES / Dens) / Vol * 100

Tmed_relvol = (1 / (CM + 1)) * (WW_g * TES / Dens) / Vol * 100

; Thymocyte percentages

T_thym_all = TDN + TDP + TSP4 + TSP8;

TSP = TSP4 + TSP8;

TDN_perc = TDN/T_thym_all * 100;

TDP_perc = TDP/T_thym_all * 100;

TSP_perc = TSP/T_thym_all * 100;

TSP4_perc = TSP4/T_thym_all * 100;

TSP8_perc = TSP8/T_thym_all * 100;

T_cell_dens = T_thym_all * 10^-6 / WW_g

; Blood volume vs age equations

if age < TM50_WT1

hill1 = hill1_WT1;

else

hill1 = hill2_WT1;

end

if age < TM50_WT2

hill2 = hill1_WT2;

else

hill2 = hill2_WT2;

end

if age < TM50_WT3

hill3 = hill1_WT3;

else

hill3 = hill2_WT3;

end

if age < TM50_WT3

WT4 = 0;

else

WT4 = WTmax4*(1 - exp(-log(2)/THALF_WT4*(age-TM50_WT3)));

end

WT1 = WTmax1/(1+(TM50_WT1/age)^hill1);

WT2 = WTmax2/(1+(TM50_WT2/age)^hill2);

WT3 = WTmax3/(1+(TM50_WT3/age)^hill3);

WT = WT1 + WT2 + WT3 + WT4;

BV = BV_BL + k_BV * WT;

; CD4+ T-lymphocyte homeostasis submodel equations

; Explicit functions

T4_lt = T_N4_lt + T_CM4_lt + T_EM4_lt + T_EFF4_lt;

T4_mem_lt = T_CM4_lt + T_EM4_lt;

T4_git = T_EM4_git + T_EFF4_git + T_N4_git + T_CM4_git;

T4_mem_git = T_EM4_git + T_CM4_git;

T4_lung = T_EM4_lung + T_EFF4_lung + T_N4_lung + T_CM4_lung;

T4_mem_lung = T_EM4_lung + T_CM4_lung;

; Relative percentages of CD4+ T-lymphocyte subpopulations in organs

T_RTE4_lt_perc = T_RTE4_lt * 100 / T4_lt;

T_N4_lt_perc = T_N4_lt * 100 / T4_lt;

T_A4_lt_perc = T_A4_lt * 100 / T4_lt;

T_CM4_lt_perc = T_CM4_lt * 100 / T4_lt;

T_EM4_lt_perc = T_EM4_lt * 100 / T4_lt;

T_EFF4_lt_perc = T_EFF4_lt * 100 / T4_lt;

T_EM4_git_perc = T_EM4_git * 100 / T4_git;

T_EFF4_git_perc = T_EFF4_git * 100 / T4_git;

T_N4_git_perc = T_N4_git * 100 / T4_git;

T_CM4_git_perc = T_CM4_git * 100 / T4_git;

T_EM4_lung_perc = T_EM4_lung * 100 / T4_lung;

T_EFF4_lung_perc = T_EFF4_lung * 100 / T4_lung;

T_N4_lung_perc = T_N4_lung * 100 / T4_lung;

T_CM4_lung_perc = T_CM4_lung * 100 / T4_lung;

T_MEM4_lt_perc = T4_mem_lt * 100 / T4_lt;

T_MEM4_git_perc = T4_mem_git * 100 / T4_git;

T_MEM4_lung_perc = T4_mem_lung * 100 / T4_lung;

; Blood concentration (cells/uL) of CD4+ T-lymphocyte subpopulations

T_RTE4_bl_cells_uL = T_RTE4_bl / (BV * 10^3);

T_N4_bl_cells_uL = T_N4_bl / (BV * 10^3);

T_A4_bl_cells_uL = T_A4_bl / (BV * 10^3);

T_CM4_bl_cells_uL = T_CM4_bl / (BV * 10^3);

T_EM4_bl_cells_uL = T_EM4_bl / (BV * 10^3);

T_EFF4_bl_cells_uL = T_EFF4_bl / (BV * 10^3);

T4_bl_cells_uL = T_N4_bl_cells_uL + T_CM4_bl_cells_uL + T_EM4_bl_cells_uL + T_EFF4_bl_cells_uL;

T_MEM4_bl_cells_uL = T_CM4_bl_cells_uL + T_EM4_bl_cells_uL;

; Initial conditions

t_0 = 0

TDN_0 = 0;

TDP_0 = 0;

TSP4_0 = 0;

TSP8_0 = 0;

T_RTE4_bl_0 = 0;

T_RTE4_lt_0 = 0;

T_N4_lt_0 = 0;

T_N4_bl_0 = 0;

T_N4_git_0 = 0;

T_N4_lung_0 = 0;

T_A4_lt_0 = 0;

T_A4_bl_0 = 0;

T_CM4_lt_0 = 0;

T_CM4_bl_0 = 0;

T_CM4_git_0 = 0;

T_CM4_lung_0 = 0;

T_EM4_lt_0 = 0;

T_EM4_bl_0 = 0;

T_EM4_git_0 = 0;

T_EM4_lung_0 = 0;

T_EFF4_lt_0 = 0;

T_EFF4_bl_0 = 0;

T_EFF4_git_0 = 0;

T_EFF4_lung_0 = 0;

; Differential equations

; thymus submodel

ddt_TDN = fi_t*(1 - (TDN+TDP)/Tcort) - fi1*TDN + lambda1*(1 - (TDN+TDP)/Tcort)*TDN - m1*TDN;

ddt_TDP = fi1*TDN + lambda2*(1 - (TDN+TDP)/Tcort)*TDP - (fi4 + fi8)*TDP*(1 - (TSP4 + TSP8) / Tmed) - m2*TDP;

ddt_TSP4 = fi4*(1 - (TSP4 + TSP8) / Tmed)*TDP - exp4*TSP4 + lambda4*(1 - (TSP4 + TSP8) / Tmed)*TSP4 - m4*TSP4;

ddt_TSP8 = fi8*(1 - (TSP4 + TSP8) / Tmed)*TDP - exp8*TSP8 + lambda8*(1 - (TSP4 + TSP8) / Tmed)*TSP8 - m8*TSP8;

; RTE CD4+ T-lymphocytes

ddt_T_RTE4_bl = exp4*TSP4 - m_rte4*T_RTE4_bl - omega_rte4_bl_lt*T_RTE4_bl;

ddt_T_RTE4_lt = omega_rte4_bl_lt*T_RTE4_bl - fi_rte4*T_RTE4_lt - m_rte4*T_RTE4_lt;

; Naïve CD4+ T-lymphocytes

ddt_T_N4_lt = fi_rte4*T_RTE4_lt - m_n4*T_N4_lt - omega_n4_lt_bl*T_N4_lt + omega_n4_bl_lt*T_N4_bl - fi_n4*T_N4_lt + lambda_n4*T_N4_lt;

ddt_T_N4_bl = omega_n4_lt_bl*T_N4_lt - omega_n4_bl_lt*T_N4_bl + omega_n4_git_bl*T_N4_git - omega_n4_bl_git*T_N4_bl + omega_n4_lung_bl*T_N4_lung - omega_n4_bl_lung*T_N4_bl - omega_n4_bl_tis*T_N4_bl - m_n4*T_N4_bl;

ddt_T_N4_git = omega_n4_bl_git*T_N4_bl - omega_n4_git_bl*T_N4_git - m_n4*T_N4_git;

ddt_T_N4_lung = omega_n4_bl_lung*T_N4_bl - omega_n4_lung_bl*T_N4_lung - m_n4*T_N4_lung;

; Activated CD4+ T-lymphocytes

ddt_T_A4_lt = fi_n4*T_N4_lt - m_a4*T_A4_lt + lambda_a4*T_A4_lt*(1 - T_A4_lt/T_A4_max) - omega_a4_lt_bl*T_A4_lt + omega_a4_bl_lt*T_A4_bl - fi_a4*T_A4_lt;

ddt_T_A4_bl = omega_a4_lt_bl*T_A4_lt - omega_a4_bl_lt*T_A4_bl - omega_a4_bl_tis*T_A4_bl - m_a4*T_A4_bl;

; Central-memory CD4+ T-lymphocytes

ddt_T_CM4_lt = f4*fi_a4*T_A4_lt - m_cm4*T_CM4_lt + lambda_cm4*T_CM4_lt - omega_cm4_lt_bl*T_CM4_lt + omega_cm4_bl_lt*T_CM4_bl - fi_cm4*T_CM4_lt;

ddt_T_CM4_bl = omega_cm4_lt_bl*T_CM4_lt - omega_cm4_bl_lt*T_CM4_bl + omega_cm4_git_bl*T_CM4_git - omega_cm4_bl_git*T_CM4_bl + omega_cm4_lung_bl*T_CM4_lung - omega_cm4_bl_lung*T_CM4_bl - omega_cm4_bl_tis*T_CM4_bl - m_cm4*T_CM4_bl;

ddt_T_CM4_git = omega_cm4_bl_git*T_CM4_bl - omega_cm4_git_bl*T_CM4_git - m_cm4*T_CM4_git;

ddt_T_CM4_lung = omega_cm4_bl_lung*T_CM4_bl - omega_cm4_lung_bl*T_CM4_lung - m_cm4*T_CM4_lung;

; Effector-memory CD4+ T-lymphocytes

ddt_T_EM4_lt = fi_cm4*T_CM4_lt - m_em4*T_EM4_lt + lambda_em4*T_EM4_lt - omega_em4_lt_bl*T_EM4_lt - fi_em4*T_EM4_lt;

ddt_T_EM4_bl = omega_em4_lt_bl*T_EM4_lt - omega_em4_bl_git*T_EM4_bl + omega_em4_git_bl*T_EM4_git - omega_em4_bl_lung*T_EM4_bl + omega_em4_lung_bl*T_EM4_lung - omega_em4_bl_tis*T_EM4_bl - m_em4*T_EM4_bl;

ddt_T_EM4_git = omega_em4_bl_git*T_EM4_bl - omega_em4_git_bl*T_EM4_git - m_em4*T_EM4_git;

ddt_T_EM4_lung = omega_em4_bl_lung*T_EM4_bl - omega_em4_lung_bl*T_EM4_lung - m_em4*T_EM4_lung;

; Effector CD4+ T-lymphocytes

ddt_T_EFF4_lt = fi_em4*T_EM4_lt + (1-f4)*fi_a4*T_A4_lt - omega_eff4_lt_bl*T_EFF4_lt - m_eff4*T_EFF4_lt;

ddt_T_EFF4_bl = omega_eff4_lt_bl*T_EFF4_lt - omega_eff4_bl_git*T_EFF4_bl - omega_eff4_bl_lung*T_EFF4_bl - omega_eff4_bl_tis*T_EFF4_bl - m_eff4*T_EFF4_bl;

ddt_T_EFF4_git = omega_eff4_bl_git*T_EFF4_bl - m_eff4*T_EFF4_git;

ddt_T_EFF4_lung = omega_eff4_bl_lung*T_EFF4_bl - m_eff4*T_EFF4_lung;

OUTPUT:

output = {T_RTE4_bl_cells_uL, T_N4_bl_cells_uL, T_A4_bl_cells_uL, T_CM4_bl_cells_uL, T_EM4_bl_cells_uL, T_EFF4_bl_cells_uL, T_RTE4_lt_perc, T_N4_lt_perc, T_A4_lt_perc, T_CM4_lt_perc, T_EM4_lt_perc, T_EFF4_lt_perc, T_EM4_git_perc, T_EFF4_git_perc, T_N4_git_perc, T_CM4_git_perc, T_EM4_lung_perc, T_EFF4_lung_perc, T_N4_lung_perc, T_CM4_lung_perc}

# References

1. Kulesh V, Peskov K, Helmlinger G, Bocharov G. Systematic review and quantitative meta-analysis of age-dependent human T-lymphocyte homeostasis. Front Immunol [Internet]. 2025 Jan 27 [cited 2025 July 4];16. Available from: https://www.frontiersin.org/journals/immunology/articles/10.3389/fimmu.2025.1475871/full

2. Brines JK, Gibson JG, Kunkel P. The blood volume in normal infants and children. J Pediatr. 1941 Apr 1;18(4):447–57.

3. Carrick-Ranson G, Hastings JL, Bhella PS, Shibata S, Fujimoto N, Palmer D, et al. The Effect of Age-related Differences in Body Size and Composition on Cardiovascular Determinants of VO2max. J Gerontol Ser A. 2013 May 1;68(5):608–16.

4. Sisson TRC, Whalen LE. The blood volume of infants: III. Alterations in the first hours after birth. J Pediatr. 1960 Jan 1;56(1):43–7.

5. Davy KP, Seals DR. Total blood volume in healthy young and older men. J Appl Physiol. 1994 May 1;76(5):2059–62.

6. Wakeham DJ, Hearon CM, Levine BD. The effect of chronic habitual exercise on oxygen carrying capacity and blood compartment volumes in older adults. J Appl Physiol. 2024 Apr 1;136(4):984–93.

7. Zisowsky J, Krause A, Dingemanse J. Drug Development for Pediatric Populations: Regulatory Aspects. Pharmaceutics. 2010 Nov 29;2(4):364–88.

8. Kulesh V, Peskov K, Helmlinger G, Bocharov G. An integrative mechanistic model of thymocyte dynamics. Front Immunol [Internet]. 2024 Feb 26 [cited 2025 July 4];15. Available from: https://www.frontiersin.org/journals/immunology/articles/10.3389/fimmu.2024.1321309/full

9. Kendall MD, Johnson HR, Singh J. The weight of the human thymus gland at necropsy. J Anat. 1980;131(3):483–97.

10. BOYD E. THE WEIGHT OF THE THYMUS GLAND IN HEALTH AND IN DISEASE. Am J Dis Child. 1932 May 1;43(5_PART_I):1162–214.

11. van Gent R, Schadenberg AWL, Otto SA, Nievelstein RAJ, Sieswerda GT, Haas F, et al. Long-term restoration of the human T-cell compartment after thymectomy during infancy: a role for thymic regeneration? Blood. 2011 July 21;118(3):627–34.

12. Silva SL, Albuquerque AS, Matoso P, Charmeteau-de-Muylder B, Cheynier R, Ligeiro D, et al. IL-7-Induced Proliferation of Human Naive CD4 T-Cells Relies on Continued Thymic Activity. Front Immunol [Internet]. 2017 Jan 19 [cited 2025 July 8];8. Available from: https://www.frontiersin.org/journals/immunology/articles/10.3389/fimmu.2017.00020/full

13. van Hoeven V, Drylewicz J, Westera L, den Braber I, Mugwagwa T, Tesselaar K, et al. Dynamics of Recent Thymic Emigrants in Young Adult Mice. Front Immunol [Internet]. 2017 Aug 7 [cited 2025 July 8];8. Available from: https://www.frontiersin.org/journals/immunology/articles/10.3389/fimmu.2017.00933/full

14. Haines CJ, Giffon TD, Lu LS, Lu X, Tessier-Lavigne M, Ross DT, et al. Human CD4+ T cell recent thymic emigrants are identified by protein tyrosine kinase 7 and have reduced immune function. J Exp Med. 2009 Feb 16;206(2):275–85.

15. Vrisekoop N, den Braber I, de Boer AB, Ruiter AFC, Ackermans MT, van der Crabben SN, et al. Sparse production but preferential incorporation of recently produced naïve T cells in the human peripheral pool. Proc Natl Acad Sci. 2008 Apr 22;105(16):6115–20.

16. Richman DD. Normal physiology and HIV pathophysiology of human T-cell dynamics. J Clin Invest. 2000 Mar 1;105(5):565–6.

17. Michie CA, McLean A, Alcock C, Beverley PCL. Lifespan of human lymphocyte subsets defined by CD45 isoforms. Nature. 1992 Nov 1;360(6401):264–5.

18. Asquith B, Debacq C, Macallan DC, Willems L, Bangham CRM. Lymphocyte kinetics: the interpretation of labelling data. Trends Immunol. 2002 Dec 1;23(12):596–601.

19. Macallan DC, Asquith B, Irvine AJ, Wallace DL, Worth A, Ghattas H, et al. Measurement and modeling of human T cell kinetics. Eur J Immunol. 2003 Aug 1;33(8):2316–26.

20. Macallan DC, Wallace D, Zhang Y, de Lara C, Worth AT, Ghattas H, et al. Rapid Turnover of Effector–Memory CD4+ T Cells in Healthy Humans. J Exp Med. 2004 July 19;200(2):255–60.

21. Wallace DL, Zhang Y, Ghattas H, Worth A, Irvine A, Bennett AR, et al. Direct Measurement of T Cell Subset Kinetics In Vivo in Elderly Men and Women1. J Immunol. 2004 Aug 1;173(3):1787–94.

22. Mueller-Schoell A, Puebla-Osorio N, Michelet R, Green MR, Künkele A, Huisinga W, et al. Early Survival Prediction Framework in CD19-Specific CAR-T Cell Immunotherapy Using a Quantitative Systems Pharmacology Model. Cancers. 2021;13(11).

23. Schubert R, Reichenbach J, Royer N, Pichler M, Zielen S. Spontaneous and oxidative stress-induced programmed cell death in lymphocytes from patients with ataxia telangiectasia (AT). Clin Exp Immunol. 2000 Jan;119(1):140–7.

24. Mclean AR, Michie CA. In vivo estimates of division and death rates of human T lymphocytes. Proc Natl Acad Sci U S A. 1995 Apr 25;92(9):3707–11.

25. Macallan DC, Busch R, Asquith B. Current estimates of T cell kinetics in humans. Curr Opin Syst Biol. 2019 Dec;18:77–86.

26. Kaech SM, Wherry EJ, Ahmed R. Effector and memory T-cell differentiation: implications for vaccine development. Nat Rev Immunol. 2002 Apr 1;2(4):251–62.

27. Ganusov VV, Tomura M. Experimental and Mathematical Approaches to Quantify Recirculation Kinetics of Lymphocytes. In: Molina-París C, Lythe G, editors. Mathematical, Computational and Experimental T Cell Immunology [Internet]. Cham: Springer International Publishing; 2021. p. 151–69. Available from: https://doi.org/10.1007/978-3-030-57204-4_10

28. Bajaria SH, Webb G, Cloyd M, Kirschner D. Dynamics of Naive and Memory CD4+ T Lymphocytes in HIV-1 Disease Progression. JAIDS J Acquir Immune Defic Syndr [Internet]. 2002;30(1). Available from: https://journals.lww.com/jaids/fulltext/2002/05010/dynamics_of_naive_and_memory_cd4__t_lymphocytes_in.6.aspx

29. Sprent J. Circulating T and B lymphocytes of the mouse: I. Migratory properties. Cell Immunol. 1973 Apr 1;7(1):10–39.

30. Sprent J, Basten A. Circulating T and B lymphocytes of the mouse: II. Lifespan. Cell Immunol. 1973 Apr 1;7(1):40–59.

31. Mandl JN, Liou R, Klauschen F, Vrisekoop N, Monteiro JP, Yates AJ, et al. Quantification of lymph node transit times reveals differences in antigen surveillance strategies of naïve CD4+ and CD8+ T cells. Proc Natl Acad Sci. 2012 Oct 30;109(44):18036–41.

32. Malhotra D, Burrack KS, Jenkins MK, Frosch AE. Antigen-Specific CD4+ T Cells Exhibit Distinct Kinetic and Phenotypic Patterns During Primary and Secondary Responses to Infection. Front Immunol [Internet]. 2020;Volume 11-2020. Available from: https://www.frontiersin.org/journals/immunology/articles/10.3389/fimmu.2020.02125

33. Gossel G, Hogan T, Cownden D, Seddon B, Yates AJ. Memory CD4 T cell subsets are kinetically heterogeneous and replenished from naive T cells at high levels. de Boer R, editor. eLife. 2017 Mar 10;6:e23013.

34. Ribeiro RM, Mohri H, Ho DD, Perelson AS. In vivo dynamics of T cell activation, proliferation, and death in HIV-1 infection: Why are CD4+ but not CD8+ T cells depleted? Proc Natl Acad Sci. 2002 Nov 26;99(24):15572–7.

35. Biancotto A, Iglehart SJ, Vanpouille C, Condack CE, Lisco A, Ruecker E, et al. HIV-1–induced activation of CD4+ T cells creates new targets for HIV-1 infection in human lymphoid tissue ex vivo. Blood. 2008 Jan 15;111(2):699–704.

36. Sprent J, Miller JFAP. Fate of H2-activated T lymphocytes in syngeneic hosts: II. Residence in recirculating lymphocyte pool and capacity to migrate to allografts. Cell Immunol. 1976 Feb 1;21(2):303–13.

37. Sender R, Weiss Y, Navon Y, Milo I, Azulay N, Keren L, et al. The total mass, number, and distribution of immune cells in the human body. Proc Natl Acad Sci. 2023 Oct 31;120(44):e2308511120.

38. Chandler J, Bullock ME, Swain AC, Williams C, van Dorp CH, Seddon B, et al. Tissue-resident memory CD4+ T cells are sustained by site-specific levels of self-renewal and continuous replacement. Belz GT, Taniguchi T, editors. eLife. 2025 June 25;14:RP104278.

39. Rangarajan A, Weinberg RA. Comparative biology of mouse versus human cells: modelling human cancer in mice. Nat Rev Cancer. 2003 Dec 1;3(12):952–9.

40. Perelson AS, Wiegel FW. Scaling Aspects of Lymphocyte Trafficking. J Theor Biol. 2009 Mar 7;257(1):9–16.

41. Sumpter AL, Holford NHG. Predicting weight using postmenstrual age – neonates to adults. Pediatr Anesth. 2011 Mar 1;21(3):309–15.
